# Supplementary material for: The onset of circulation triggers a metabolic switch required for endothelial to hematopoietic transition
Source: Cell Rep. 2021 Dec 14;37(11):110103. doi: 10.1016/j.celrep.2021.110103 (PMC8692754; doi:10.1016/j.celrep.2021.110103)
Supplement: Document S1. Figures S1–S7 [file mmc1.pdf]

Cell Reports, Volume *37*

Supplemental information

The onset of circulation triggers a metabolic switch required for endothelial to hematopoietic transition

Emanuele Azzoni, Vincent Frontera, Giorgio Anselmi, Christina Rode, Chela James, Elitza M. Deltcheva, Atanasiu S. Demian, John Brown, Cristiana Barone, Arianna Patelli, Joe R. Harman, Matthew Nicholls, Simon J. Conway, Edward Morrissey, Sten Eirik W. Jacobsen, Duncan B. Sparrow, Adrian L. Harris, Tariq Enver, and Marella F.T.R. de Bruijn

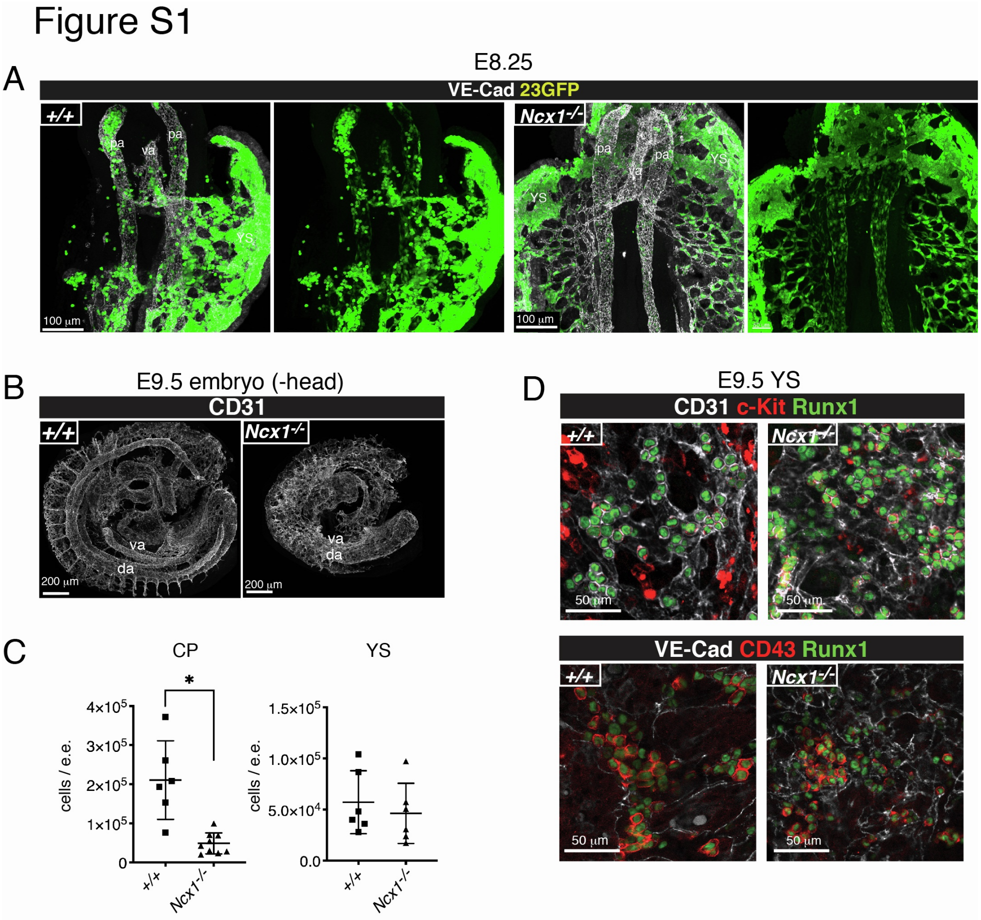

Figure S1. Additional phenotypic analysis of E9.5 *Ncx1*$^{-/-}$ embryos and yolk sacs, Related to Figure 1

A. Confocal whole mount immunofluorescence (WM-IF) analysis of E8.25 (4-8sp) wild type (+/+) and *Ncx1*$^{-/-}$ concepti (embryo and yolk sac). Paired aortae (pa); vitelline artery (va). Abundant 23GFP expression can be seen in the yolk sac (YS) of both genotypes, reflecting expression in primitive erythroid cells and a subset of endothelium; in the paired aortae 23GFP marks pre-HE (Swiers et al., 2013). Note the absence of 23GFP^{+} primitive erythrocytes in the paired aortae of the *Ncx1*$^{-/-}$ embryo. N=2 (+/+), N=2 (*Ncx1*$^{-/-}$) embryos analyzed. Scale bars: 100µm.

B. Confocal WM-IF analysis of E9.5 (23-24sp) wild type (+/+) and *Ncx1*$^{-/-}$ embryos. 3D maximum intensity projection is shown. va: vitelline artery; da: dorsal aorta. Scale bars: 200□m. N≥8 (+/+), N≥8 (*Ncx1*$^{-/-}$).

C. Total live cell numbers of wild type (+/+) and *Ncx1*$^{-/-}$ E9.5 (21-27sp) caudal parts (CP) and yolk sac (YS). N=6 (+/+), N=9 (*Ncx1*$^{-/-}$) CP; N=6 (+/+), N=6 (*Ncx1*$^{-/-}$) YS. Data are represented as mean±SD.

D. Confocal WM-IF analysis of E9.5 (21-26sp) wild type (+/+) and *Ncx1*$^{-/-}$ YS, showing a single 2.5µm-thick optical slice. N=4 (+/+), N=3 (*Ncx1*$^{-/-}$) (top); N=2 (+/+), N=2 (*Ncx1*$^{-/-}$) (bottom) embryos analyzed. Scale bars: 50µm.

Figure S2

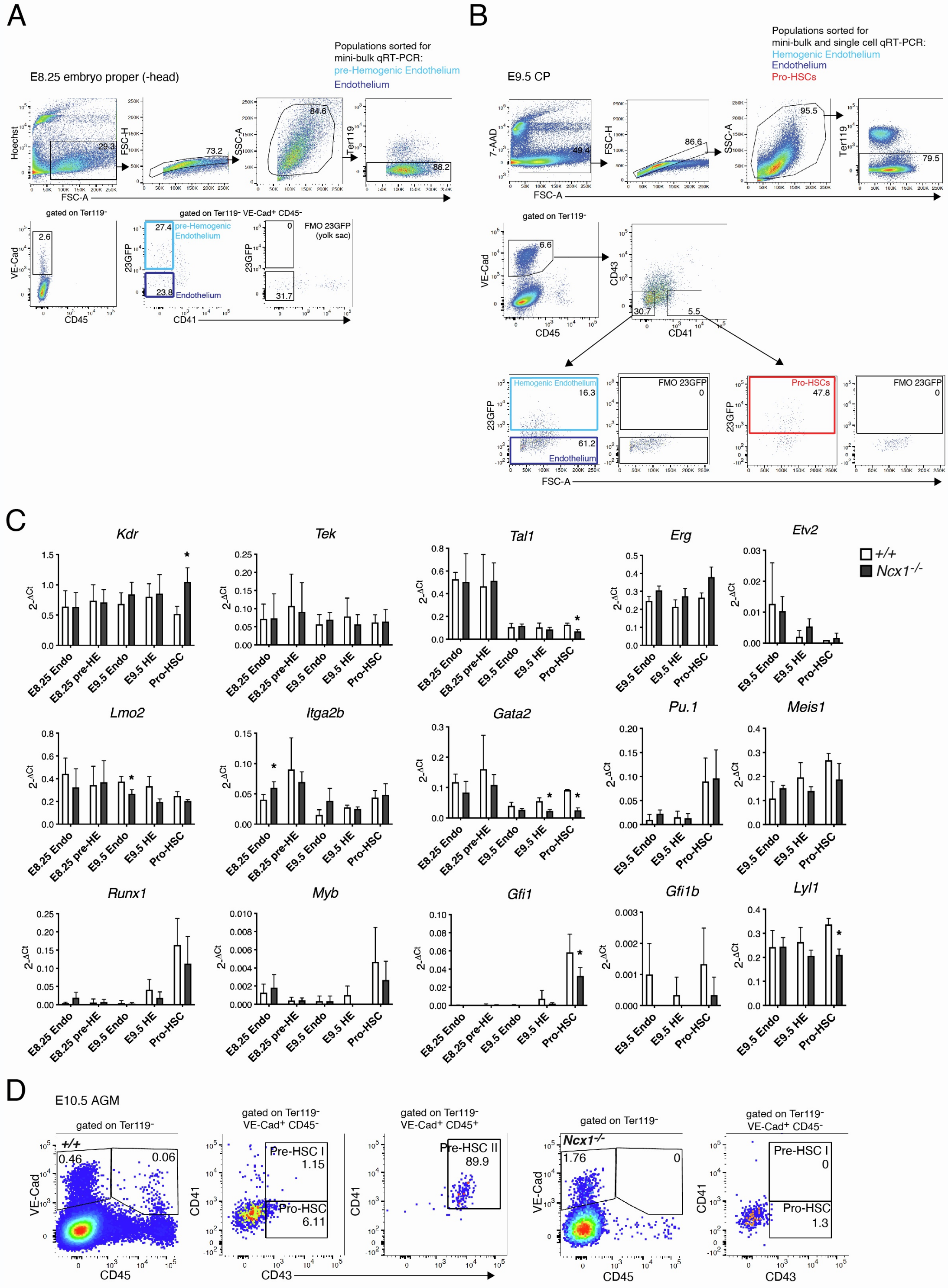

A
E8.25 embryo proper (-head)
Populations sorted for mini-bulk qRT-PCR:
pre-Hemogenic Endothelium
Endothelium
gated on Ter119⁻
gated on Ter119⁻ VE-Cad⁺ CD45⁻
FMO 23GFP (yolk sac)
B
E9.5 CP
Populations sorted for mini-bulk and single cell qRT-PCR:
Hemogenic Endothelium
Endothelium
Pro-HSCs
gated on Ter119⁻
FMO 23GFP
C
Kdr
Tek
Tal1
Erg
Etv2
+/+
Ncx1-/-
Lmo2
Itga2b
Gata2
Pu.1
Meis1
Runx1
Myb
Gfi1
Gfi1b
Lyl1
E8.25 Endo
E8.25 pre-HE
E9.5 Endo
E9.5 HE
Pro-HSC
D
E10.5 AGM
gated on Ter119⁻
gated on Ter119⁻ VE-Cad⁺ CD45⁻
gated on Ter119⁻ VE-Cad⁺ CD45⁺
Pre-HSC I
Pro-HSC
Pre-HSC II

Figure S2. Gating strategy and additional qRT-PCR and flow cytometry analysis of wild type and *Ncx1*$^{-/-}$ embryos, Related to Figure 2

A. Example of FACS gating strategy used for isolation of cells for downstream mini-bulk qRT-PCR from E8.25 embryos. A *Ncx1*$^{+/+}$ 23GFP-transgenic sample is shown. FMO 23GFP is a full staining performed on wild type or *Ncx1*$^{+/-}$ samples not carrying the 23GFP transgene. Endothelium (Ter119$^-$ VE-Cad$^+$ CD45$^-$ CD41$^-$ 23GFP$^-$); pre-hemogenic endothelium (Ter119$^-$ VE-Cad$^+$ CD45$^-$ CD41$^-$ 23GFP$^+$).

B. Example of FACS gating strategy used for isolation of cells for downstream single cell or mini-bulk bulk qRT-PCR from E9.5 embryos. Endothelium (Ter119$^-$ VE-Cad$^+$ CD45$^-$ CD41$^-$ CD43$^-$ 23GFP$^-$), Hemogenic endothelium (Ter119$^-$ VE-Cad$^+$ CD45$^-$ CD41$^-$ CD43$^-$ 23GFP$^+$), Pro-HSCs (Ter119$^-$ VE-Cad$^+$ CD45$^-$ CD41^{low} CD43$^-$ 23GFP$^+$). A *Ncx1*$^{+/+}$ 23GFP-transgenic sample is shown. FMO 23GFP is a full staining performed on wild type or *Ncx1*$^{+/-}$ samples not carrying the 23GFP transgene. Refer to individual experiments for replicate and embryo numbers.

C. Multiplexed mini-bulk qRT-PCR analysis performed on pools of 25 cells. E8.25 pre-hemogenic endothelium (pre-HE; Ter119$^-$ VE-Cad$^+$ CD45$^-$ CD41$^-$ 23GFP$^+$); E8.25 endothelium (Endo; Ter119$^-$ VE-Cad$^+$ CD45$^-$ CD41$^-$ 23GFP$^-$); E9.5 endothelium (Endo; Ter119$^-$ VE-Cad$^+$ CD45$^-$ CD41$^-$ CD43$^-$ 23GFP$^-$); E9.5 hemogenic endothelium (HE; Ter119$^-$ VE-Cad$^+$ CD45$^-$ CD41$^-$ CD43$^-$ 23GFP$^+$); E9.5 pro-HSCs (Ter119$^-$ VE-Cad$^+$ CD45$^-$ CD41^{low} CD43$^-$ 23GFP$^+$). Number of biological replicates and embryos as follows:

E8.25: N=3 (+/+, 3-10sp), N=4 (*Ncx1*$^{-/-}$, 6-11sp) biological replicates over 3 independent sorting experiments. 2 to 5 embryos of the same genotype were pooled. Total number of embryos used: 11 (+/+), 12 (*Ncx1*$^{-/-}$);

E9.5 except *Runx1*: N=3 (+/+, 21-26sp), N=3 (*Ncx1*$^{-/-}$, 22-25sp) biological replicates over 2 independent sorting experiments. 1 to 3 embryos of the same genotype were pooled. Total number of embryos used: 7 (+/+), 5 (*Ncx1*$^{-/-}$);

E9.5 *Runx1*: N=7 (+/+, 21-26sp), N=7 (*Ncx1*$^{-/-}$, 22-25sp) biological replicates over 5 independent sorting experiments. 1 to 3 embryos of the same genotype were pooled. Total number of embryos used: 13 (+/+), 15 (*Ncx1*$^{-/-}$). Data are shown as mean ±SD.

D. Flow cytometric analysis of E10.5 control (*Ncx1*$^{+/+}$ or *Ncx1*$^{+/-}$) or *Ncx1*$^{-/-}$ AGM. Wild type embryos had 35-39sp. It was not possible to count somite pairs in littermate E10.5 *Ncx1*$^{-/-}$ embryos as they were not visible. N=7 (+/+ or *Ncx1*$^{+/-}$), N=2 (*Ncx1*$^{-/-}$) over 2 independent experiments.

Figure S3

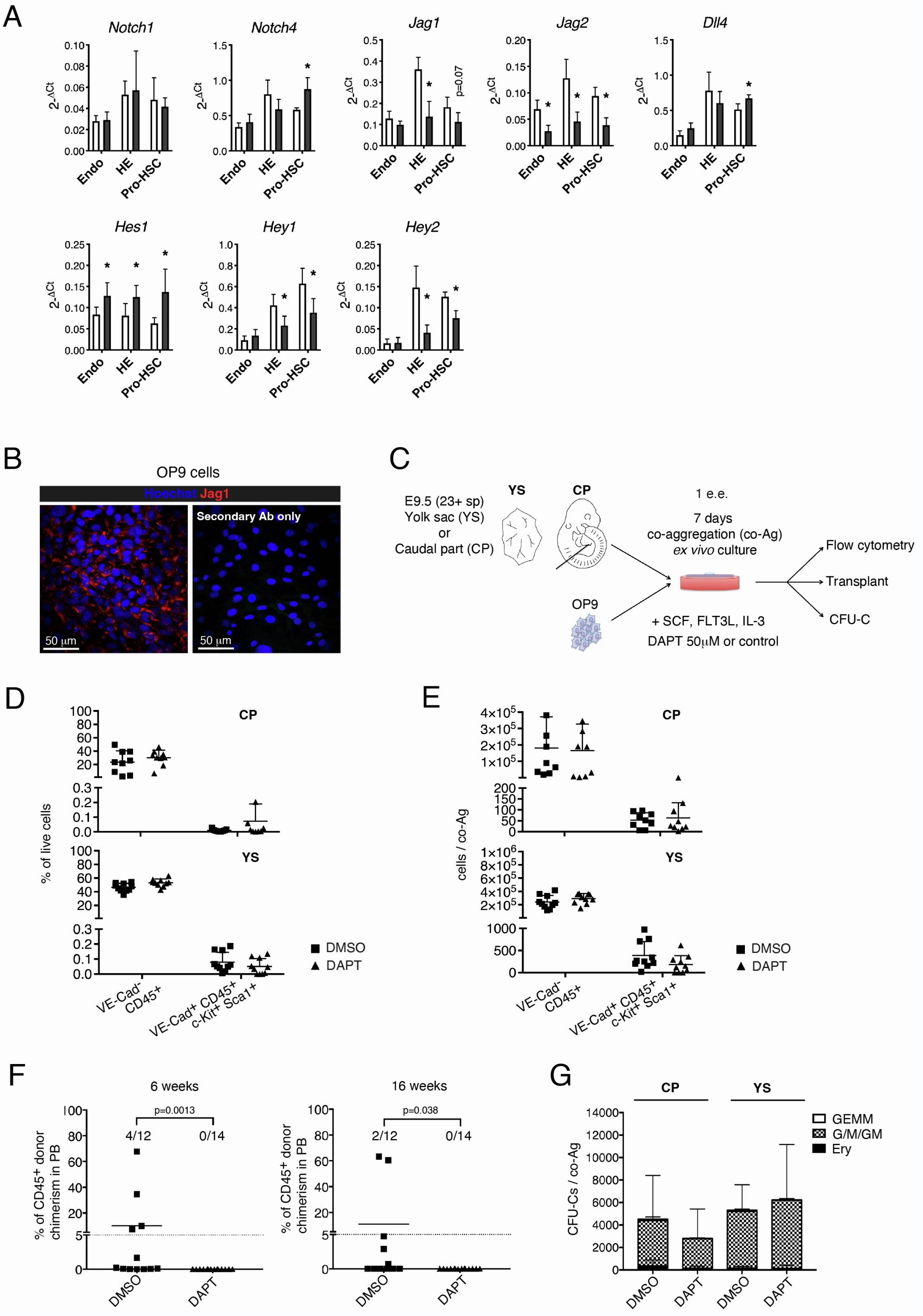
A
Notch1
Notch4
Jag1
Jag2
Dll4
Hes1
Hey1
Hey2
2-ΔCt
Endo
HE
Pro-HSC
p=0.07
B
OP9 cells
Hoechst Jag1
Secondary Ab only
50 μm
C
E9.5 (23+ sp)
Yolk sac (YS)
or
Caudal part (CP)
YS
CP
OP9
1 e.e.
7 days
co-aggregation (co-Ag)
ex vivo culture
+ SCF, FLT3L, IL-3
DAPT 50μM or control
Flow cytometry
Transplant
CFU-C
D
% of live cells
CP
YS
DMSO
DAPT
VE-Cad- CD45+
VE-Cad+ CD45+ c-Kit+ Sca1+
E
cells / co-Ag
CP
YS
DMSO
DAPT
VE-Cad- CD45+
VE-Cad+ CD45+ c-Kit+ Sca1+
F
6 weeks
16 weeks
% of CD45+ donor chimerism in PB
p=0.0013
p=0.038
4/12
0/14
2/12
0/14
DMSO
DAPT
G
CP
YS
CFU-Cs / co-Ag
GEMM
G/M/GM
Ery
DMSO
DAPT

Figure S3. The Notch pathway is required along the pro-HSC to HSC maturation route, but it is dispensable for hematopoietic progenitors, Related to Figure 2.

A. Multiplexed qRT-PCR analysis performed on pools of 25 cells. E9.5 endothelium (Endo; $Ter119^-$ $VE\text{-}Cad^+$ $CD45^-$ $CD41^-$ $CD43^-$ $23GFP^-$); E9.5 hemogenic endothelium (HE; $Ter119^-$ $VE\text{-}Cad^+$ $CD45^-$ $CD41^-$ $CD43^-$ $23GFP^+$); E9.5 pro-HSCs ($Ter119^-$ $VE\text{-}Cad^+$ $CD45^-$ $CD41^{low}$ $CD43^-$ $23GFP^+$). Sort gates as in Figure S2. N=4 (+/+, 22-26sp), N=4 (*Ncx1*$^{-/-}$, 22-25sp) biological replicates over 3 independent sorting experiments. 1 to 3 embryos of the same genotype were pooled. Total number of embryos used: 6 (+/+), 10 (*Ncx1*$^{-/-}$). Data are shown as mean±SD.

B. Immunofluorescence analysis of OP9 cells using a Jag1-specific antibody. A secondary only control (donkey anti-goat AF555) is shown. Scale bar: 50μm.

C. Schematic of co-aggregate cultures in wild type embryos with or without addition of DAPT.

D-E. Flow cytometric analysis of OP9 co-aggregates from caudal part (CP) and yolk sac (YS) of E9.5 (23-26sp) wild type embryos, treated with 50μM DAPT or DMSO. Cells were gated as $7AAD^-$ $Ter119^-$; the gating strategy was the same as in Figure 4B. Graphs show percentages of live cells (D) or absolute numbers (E) of selected populations. OP9 co-aggregates were made with 1 e.e. of caudal part or YS cells and were analyzed individually. N=9 (DMSO), N=9 (DAPT) for CP, N=10 (DMSO), N=10 (DAPT) for YS over 3 independent experiments. Statistical tests showed no significant differences between control and DAPT-treated samples. Error bars represent mean±SD.

F. Repopulation analysis of irradiated CD45.1 syngeneic mice transplanted with 1 embryo equivalent (e.e.) of E9.5 (23-28sp) wild type $CD45.2^+$ caudal part cells after co-aggregation culture, treated with 50μM DAPT or DMSO. Graphs shows peripheral blood (PB) chimerism represented as the percentage of donor $CD45.2^+$ cells among total $CD45^+$ cells, 6 (left) and 16 weeks (right) after transplant. Numbers of transplanted mice as shown. Data from 3 independent transplant experiments. Lines represent the mean.

G. CFU-C obtained from OP9 co-aggregates with caudal part and yolk sac of E9.5 (23-26sp) wild type embryos, treated with 50μM DAPT or DMSO. N=9 (DMSO), N=9 (DAPT) for CP, N=10 (DMSO), N=10 (DAPT) for YS over 3 independent experiments. GEMM: granulocyte, erythroid, monocyte/macrophage, megakaryocyte; G/M/GM: granulocyte, monocyte/macrophage; Ery: erythroid. Statistical tests showed no significant differences between control and DAPT-treated samples. Data are represented as mean± SD.

Figure S4

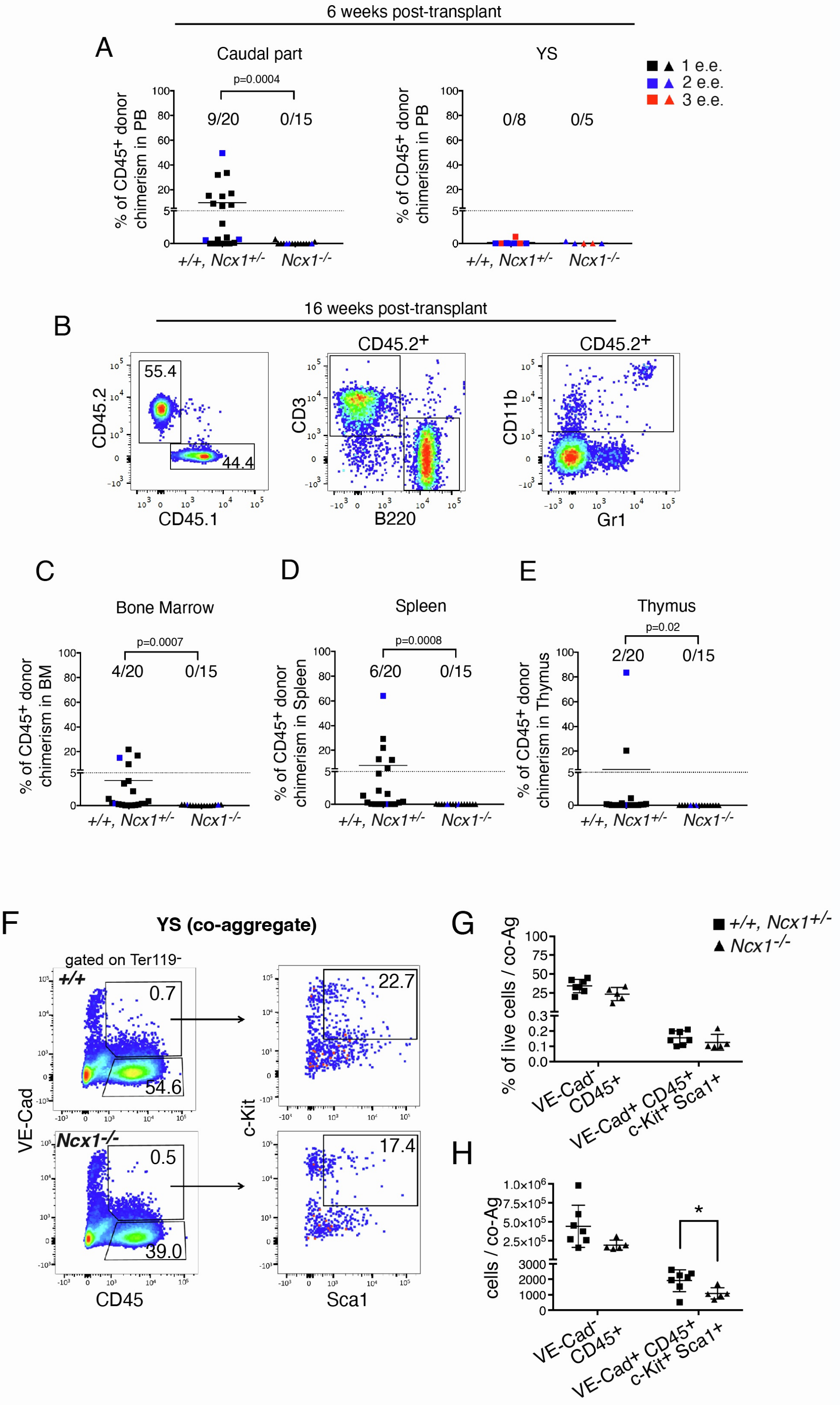
6 weeks post-transplant
A
Caudal part
YS
p=0.0004
9/20
0/15
0/8
0/5
% of CD45+ donor chimerism in PB
+/+, Ncx1+/-
Ncx1-/-
1 e.e.
2 e.e.
3 e.e.
16 weeks post-transplant
B
CD45.2+
55.4
44.4
CD45.2
CD45.1
CD3
B220
CD11b
Gr1
C
D
E
Bone Marrow
Spleen
Thymus
p=0.0007
p=0.0008
p=0.02
4/20
0/15
6/20
2/20
% of CD45+ donor chimerism in BM
% of CD45+ donor chimerism in Spleen
% of CD45+ donor chimerism in Thymus
F
YS (co-aggregate)
gated on Ter119-
+/+
Ncx1-/-
0.7
54.6
22.7
0.5
39.0
17.4
VE-Cad
CD45
c-Kit
Sca1
G
H
% of live cells / co-Ag
cells / co-Ag
VE-Cad- CD45+
VE-Cad+ CD45+ c-Kit+ Sca1+
*

Figure S4. Additional analysis of co-aggregates, Related to Figure 4

A. Repopulation analysis of irradiated CD45.1 syngeneic mice transplanted with 1-3 e.e. of E9.5 (21-27sp) control (*Ncx1*$^{+/+}$ or *Ncx1*$^{+/-}$) or *Ncx1*$^{-/-}$ CD45.2^{+} caudal part or yolk sac cells after co-aggregate culture. Graphs shows peripheral blood (PB) chimerism represented as the percentage of donor CD45.2^{+} cells on total CD45^{+} cells, 6 weeks after transplant. Number of transplanted mice as shown. Lines represent the mean.

B. Flow cytometry analysis of peripheral blood at 16 weeks post-transplant showing a representative example of a recipient reconstituted with wild type (+/+) cells.

C-E. Repopulation analysis as in (A), showing percentage of chimerism in bone marrow (C), spleen (D) and thymus (E), 16 weeks after transplant. Black squares or triangles: recipient transplanted with 1 e.e. of donor cells; Blue squares or triangles: recipient transplanted with 2 e.e. of donor cells. Lines represent the mean.

F. Flow cytometric analysis of OP9 co-aggregates from E9.5 (24-28sp) control (*Ncx1*$^{+/+}$ or *Ncx1*$^{+/-}$) and *Ncx1*$^{-/-}$ yolk sacs. Percentages of individual gates are shown in dot plots. Cells were gated as 7AAD^{-} Ter119^{-}. OP9 co-aggregates were made with 1 e.e. YS cells and were analyzed individually. N=7 (+/+ or *Ncx1*$^{+/-}$), N=5 (*Ncx1*$^{-/-}$) over 3 independent experiments.

G-H. Graphs of flow cytometric analysis in (F) showing percentage within live cells (G) and absolute numbers (H) of VE-Cad^{-} CD45^{+} and VE-Cad^{+} CD45^{+} c-Kit^{+} Sca1^{+} cells. Error bars in the graphs represent mean±SD.

Figure S5

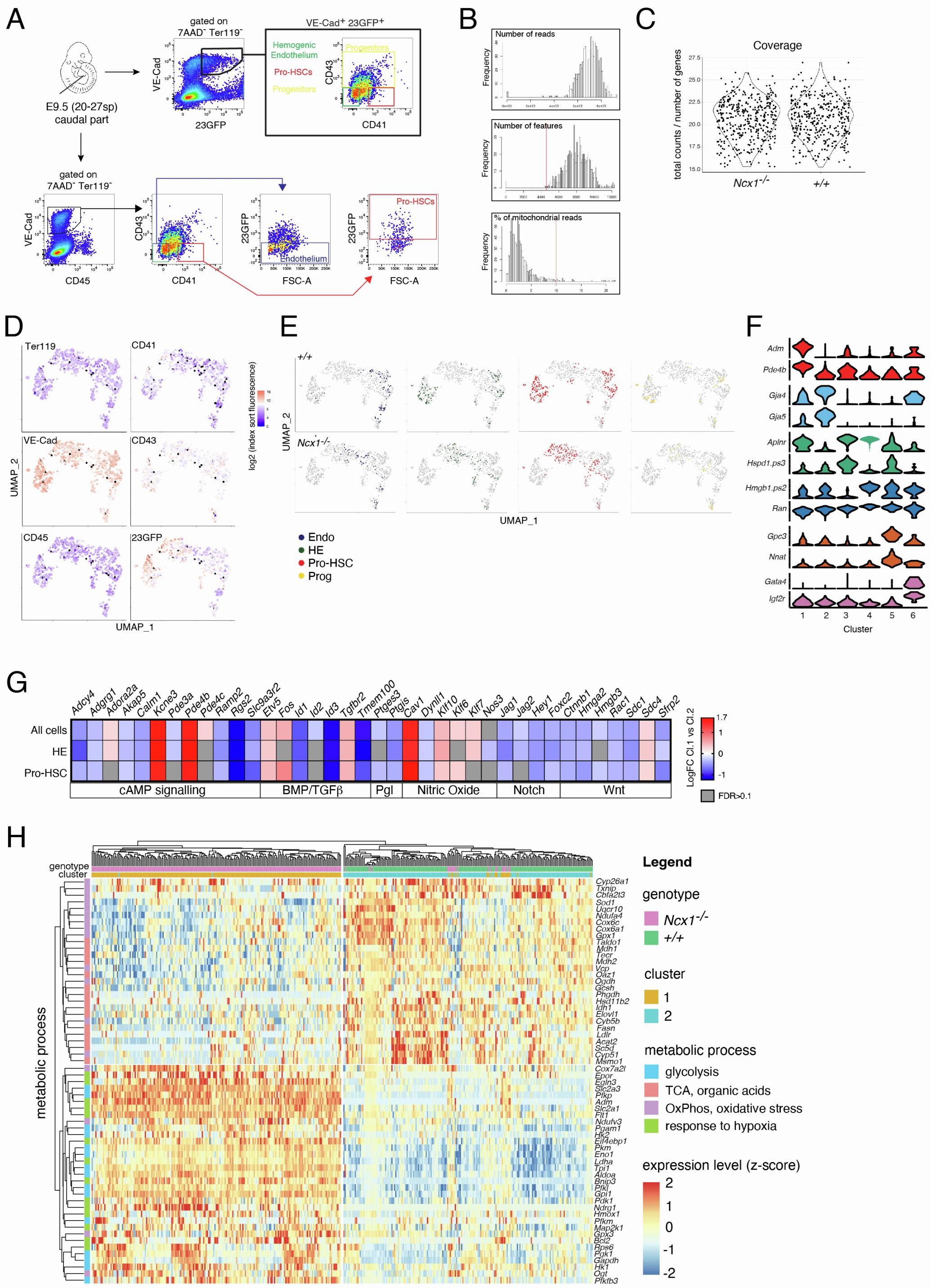

A
E9.5 (20-27sp) caudal part
gated on 7AAD- Ter119-
VE-Cad
23GFP
VE-Cad+ 23GFP+
Hemogenic Endothelium
Pro-HSCs
Progenitors
CD43
CD41
CD45
FSC-A
Endothelium
B
Number of reads
Number of features
% of mitochondrial reads
Frequency
C
Coverage
total counts / number of genes
Ncx1-/-
+/+
D
Ter119
CD41
VE-Cad
CD43
CD45
23GFP
UMAP_2
UMAP_1
log2 (index sort fluorescence)
E
+/+
Ncx1-/-
Endo
HE
Pro-HSC
Prog
F
Adm
Pde4b
Gja4
Gja5
Aplnr
Hspd1.ps3
Hmgb1.ps2
Ran
Gpc3
Nnat
Gata4
Igf2r
Cluster
G
All cells
HE
Pro-HSC
cAMP signalling
BMP/TGFβ
Pgl
Nitric Oxide
Notch
Wnt
LogFC Cl.1 vs Cl.2
FDR>0.1
H
genotype
cluster
metabolic process
Legend
glycolysis
TCA, organic acids
OxPhos, oxidative stress
response to hypoxia
expression level (z-score)

Figure S5. Sort gates and additional Smart-Seq2 scRNA-seq analysis, Related to Figure 5

A. Schematic of the gates used for single cell FACS sorting. Endothelium (7-AAD$^-$ Ter119$^-$ VE-Cadherin$^+$ CD45$^-$ CD43$^-$ CD41$^-$ 23GFP$^-$) and pro-HSCs (7-AAD$^-$ Ter119$^-$ VE-Cadherin$^+$ CD45$^-$ CD43$^-$ CD41^{low} 23GFP$^+$) were sorted directly. 7-AAD$^-$ Ter119$^-$ VE-Cad$^+$ 23GFP$^+$ were index sorted and assigned to HE (7-AAD$^-$ Ter119$^-$ VE-Cadherin$^+$ CD45$^-$ CD43$^-$ CD41$^-$ 23GFP$^+$), pro-HSC (7-AAD$^-$ Ter119$^-$ VE-Cadherin$^+$ CD45$^-$ CD43$^-$ CD41^{low} 23GFP$^+$) and progenitor (7-AAD$^-$ Ter119$^-$ VE-Cadherin$^+$ CD45$^-$ CD43$^+$ CD41$^{+/-}$ 23GFP$^+$) gates. 26 E9.5 wild type (+/+*;* 21-27sp) and 19 mutant (*Ncx1*$^{-/-}$; 20-25sp) embryos were used. Cells were collected in 6 different sorting experiments.

B. Histograms showing number of reads, feature counts and percentage of mitochondrial reads across the Smart-Seq2 scRNA-seq dataset. Cells with less than 5.2×10^5 reads or 4600 features or more than 10% of mitochondrial reads (red line in the histograms) were excluded from the rest of the analysis.

C. Violin plot showing representative coverage values in the two genotypes (+/+ and *Ncx1*$^{-/-}$). For each cell, coverage is calculated as the total number of counts divided by the number of genes detected in that cell. This value represents how many counts are assigned to an individual gene on average.

D. UMAP projections of surface marker values obtained by index sorting, color coded on each cell. Black dots indicate 15 cells with missing index sort data (6 cells directly sorted as endothelial cells and 9 directly sorted as pro-HSCs).

E. UMAP projections highlighting the four phenotypically identified cell populations (Endo, HE, Pro-HSC and progenitors) with different colors, as indicated. Each of the four populations is shown separately for the two genotypes.

F. Violin plots showing expression levels of the top two genes driving clustering, across the six clusters. Each row of violin plots is color coded as in Figure 5A. Colors indicate the cluster in which each gene is enriched.

G. Heatmap representing selected genes differentially expressed (FDR<0.1) between cluster 1 (*Ncx1*$^{-/-}$) and 2 (+/+), divided in groups of genes involved in signalling pathways as indicated. cAMP genes were downregulated in *Ncx1*$^{-/-}$ cells, except for *Kcne3* and *Pde4b*, which are known to be induced by hypoxia in several cell types (Copple et al., 2011; Millen et al., 2006). Nitric oxide genes showed a mixed response, with *Nos3* (endothelial nitric oxide synthase) downregulated only in *Ncx1*$^{-/-}$ HE. Id genes were downregulated in *Ncx1*$^{-/-}$ cells, as well as the BMP target *Tmem100*, an arterial gene that when deleted showed vascular remodeling defects (Somekawa et al., 2012) similar to those seen in *Ncx1*$^{-/-}$ embryos. The prostaglandin and Wnt genes were downregulated in the *Ncx1*$^{-/-}$ cells, except for the Wnt negative regulator *Sdc4,* which was upregulated (Astudillo et al., 2014). Values are shown as logFC (fold change) and are calculated taking into account all cells in cluster 1 and 2, or only HE or pro-HSC in the same clusters. Grey cells indicate genes that are not differentially expressed (FDR>0.1) in that population.

H. Heatmap of selected genes differentially expressed between cluster 1 and cluster 2 (FDR<0.1). Rows represent genes; columns represent single cells. The color coding indicates genotypes (+/+ and *Ncx1*$^{-/-}$), clusters (1 or 2), and groups of metabolic processes (glycolysis, TCA and organic acids, OxPhos and oxidative stress, and response to hypoxia). Expression levels are shown as z-scores. TCA: tricarboxylic acid cycle; OxPhos: oxidative phosphorylation.

Figure S6

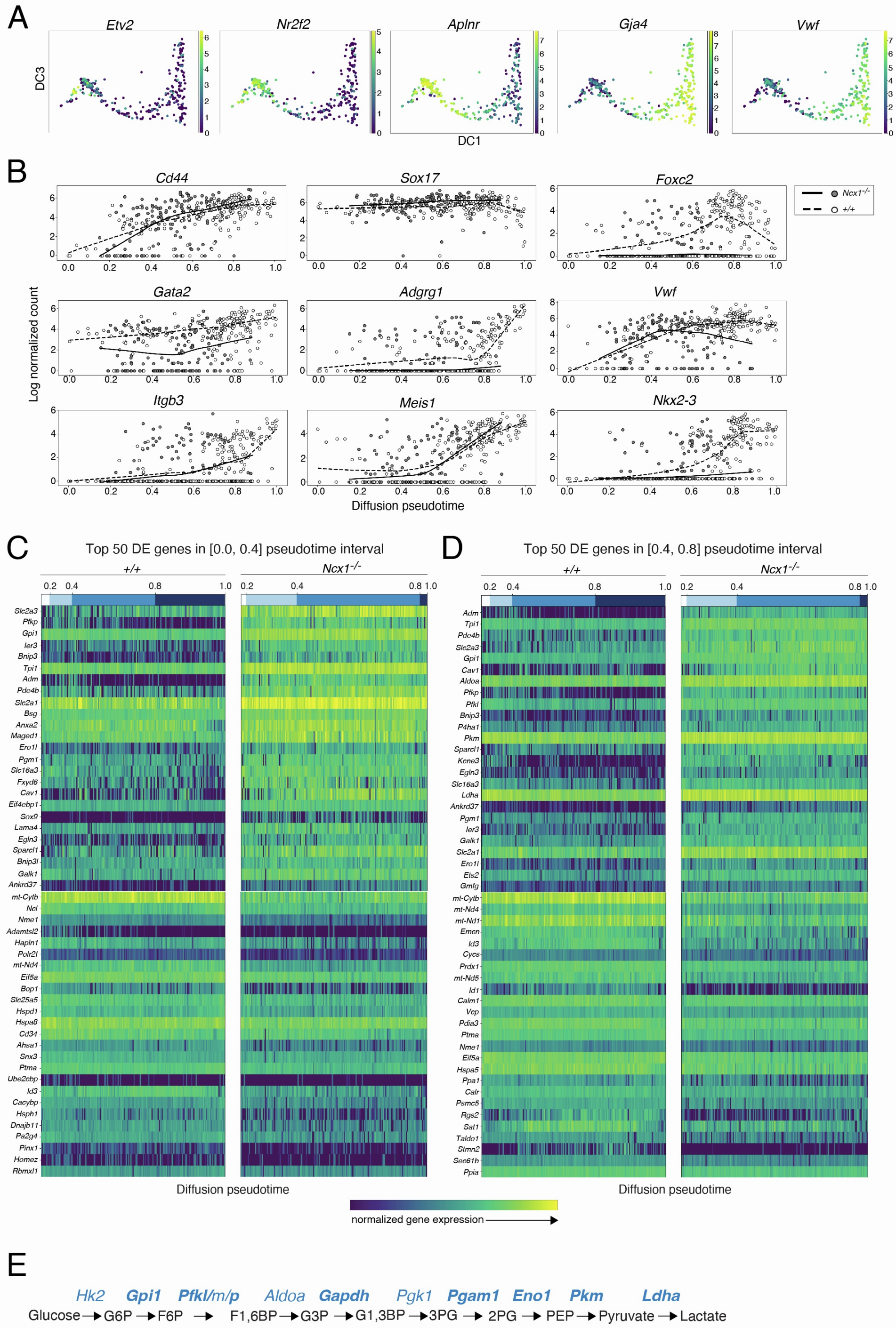
A
Etv2
Nr2f2
Aplnr
Gja4
Vwf
DC3
DC1
B
Cd44
Sox17
Foxc2
Ncx1-/-
+/+
Gata2
Adgrg1
Vwf
Itgb3
Meis1
Nkx2-3
Log normalized count
Diffusion pseudotime
C
Top 50 DE genes in [0.0, 0.4] pseudotime interval
+/+
Ncx1-/-
Slc2a3
Pfkp
Gpi1
Ier3
Bnip3
Tpi1
Adm
Pde4b
Slc2a1
Bsg
Anxa2
Maged1
Ero1l
Pgm1
Slc16a3
Fxyd6
Cav1
Eif4ebp1
Sox9
Lama4
Egln3
Sparcl1
Bnip3l
Galk1
Ankrd37
mt-Cytb
Nel
Nme1
Adamtsl2
Hapln1
Polr2l
mt-Nd4
Eif5a
Bop1
Slc25a5
Hspd1
Hspa8
Cd34
Ahsa1
Snx3
Ptma
Ube2cbp
Id3
Cacybp
Hsph1
Dnajb11
Pa2g4
Pinx1
Homez
Rbmxl1
Diffusion pseudotime
D
Top 50 DE genes in [0.4, 0.8] pseudotime interval
+/+
Ncx1-/-
Adm
Tpi1
Pde4b
Slc2a3
Gpi1
Cav1
Aldoa
Pfkp
Pfkl
Bnip3
P4ha1
Pkm
Sparcl1
Kcne3
Egln3
Slc16a3
Ldha
Ankrd37
Pgm1
Ier3
Galk1
Slc2a1
Ero1l
Ets2
Gmfg
mt-Cytb
mt-Nd4
mt-Nd1
Emcn
Id3
Cycs
Prdx1
mt-Nd5
Id1
Calm1
Vcp
Pdia3
Ptma
Nme1
Eif5a
Hspa5
Ppa1
Calr
Psmc5
Rgs2
Sat1
Taldo1
Stmn2
Sec61b
Ppia
Diffusion pseudotime
normalized gene expression
E
Hk2
Gpi1
Pfkl/m/p
Aldoa
Gapdh
Pgk1
Pgam1
Eno1
Pkm
Ldha
Glucose → G6P → F6P → F1,6BP → G3P → G1,3BP → 3PG → 2PG → PEP → Pyruvate → Lactate

Figure S6. Additional pseudotime analysis of Smart-seq2 scRNA-seq data, Related to Figure 6

A. scRNA-seq diffusion maps of wild type cells, in which expression of selected genes is super-imposed. Each dot represents an individual cell. Gene expression levels are color coded and shown as Log (normalized counts). DC: diffusion component.

B. Scatter plots showing expression of selected genes involved in hemogenic endothelium and hematopoietic development along diffusion pseudotime. Each dot represents an individual cell. Gene expression is shown in the y-axis as Log (normalized counts). Lines fitting the expression of genes over the pseudotime are obtained by locally weighted linear regression.

C-D. Heatmaps showing the top 50 genes differentially expressed between wild type and *Ncx1*$^{-/-}$ cells in the EHT trajectory (25 upregulated and 25 downregulated in *Ncx1*$^{-/-}$ versus wild type), determined separately for two regions of pseudotime: 0.0 to 0.4 (B) and 0.4 to 0.8 (C). Gene expression is color coded and shown as Log (normalized counts). Values were rescaled on a scale of 0 to 1 for visualization purpose.

E. Schematic of the glycolysis pathway, in which the genes in bold are significantly downregulated in wild type cells in the second pseudotime region (0.4 to 1.0) compared to the first (0.0 to 0.4) – see table S3.

Figure S7

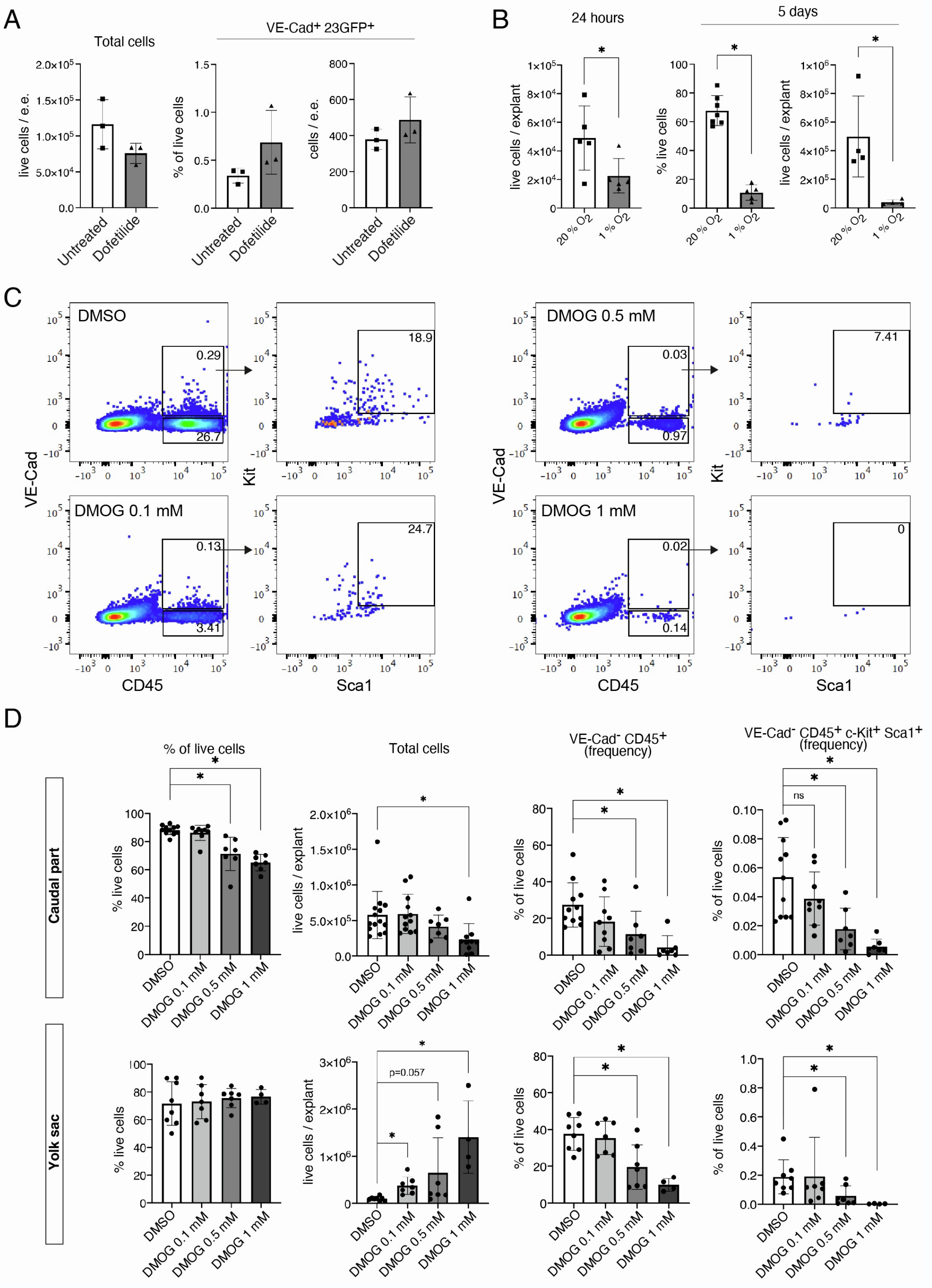

A
Total cells
VE-Cad+ 23GFP+
live cells / e.e.
% of live cells
cells / e.e.
Untreated
Dofetilide
B
24 hours
5 days
live cells / explant
% live cells
20 % O2
1 % O2
C
DMSO
DMOG 0.1 mM
DMOG 0.5 mM
DMOG 1 mM
VE-Cad
Kit
CD45
Sca1
D
% of live cells
Total cells
VE-Cad- CD45+ (frequency)
VE-Cad- CD45+ c-Kit+ Sca1+ (frequency)
Caudal part
Yolk sac
% live cells
live cells / explant
% of live cells
ns
p=0.057
DMSO
DMOG 0.1 mM
DMOG 0.5 mM
DMOG 1 mM

Figure S7. Additional analyses of dofetilide-treated embryos and hypoxia and DMOG-treated explant cultures, Related to Figure 7.

A. Cell counts and flow cytometric analysis of freshly isolated E9.5 (21-27sp) embryos from control and dofetilide-treated females. Cells were gated as live Ter119$^-$ VE-Cad$^+$ 23GFP$^+$. Numbers of replicates as shown. Data from 2 independent experiments. Statistical tests showed no significant differences between control and treated samples. Data are mean±SD.

B. Live cell counts of hypoxia explant cultures. N=5 (20% O_2), N=5 (1% O_2), 4 independent experiments for 24-hour cultures. Number of replicates as shown, 4 independent experiments for 5-day cultures. Data are mean±SD.

C. Representative dot plots of explant cultures of wild type E9.5 (19-26sp) embryos cultured in presence of a range of concentrations of DMOG. Cells were gated as Live Ter119$^-$. All dot plots are taken from the same experiment.

D. Graphs summarizing cell counts and flow cytometry analysis of DMOG explant cultures, 7 days after culture. Numbers of replicates as shown. Data from 4 independent experiments (CP and YS). Data are mean±SD.
