# Supplementary material for: The onset of circulation triggers a metabolic switch required for endothelial to hematopoietic transition
Source: Cell Rep. 2021 Dec 14;37(11):110103. doi: 10.1016/j.celrep.2021.110103 (PMC8692754; doi:10.1016/j.celrep.2021.110103)
Supplement: Document S2. Article plus supplemental information [file mmc5.pdf]

Cell Reports

Article

The onset of circulation triggers a metabolic switch required for endothelial to hematopoietic transition

Graphical abstract

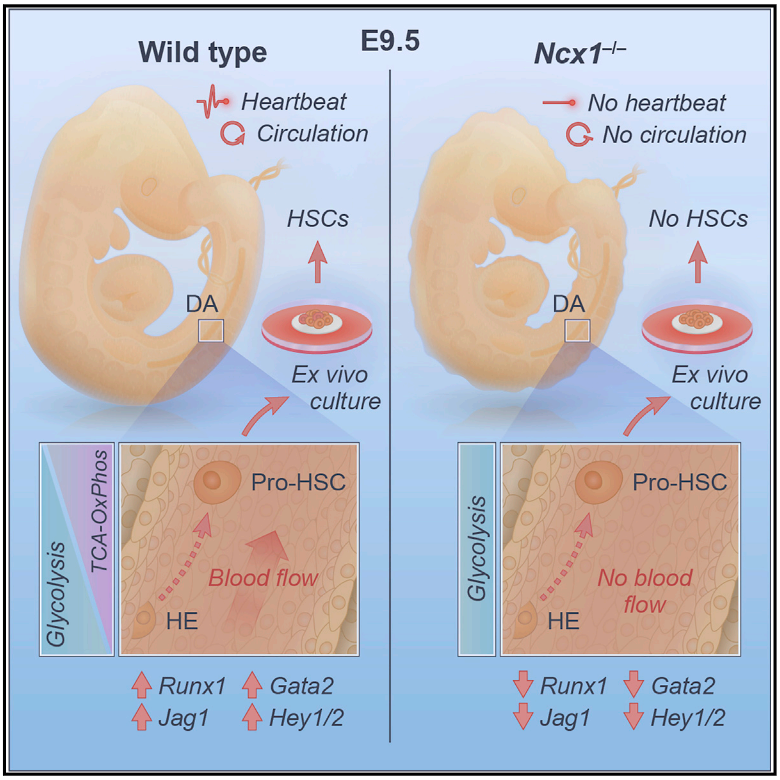

Authors

Emanuele Azzoni, Vincent Frontera, Giorgio Anselmi, ..., Adrian L. Harris, Tariq Enver, Marella F.T.R. de Bruijn

Correspondence

emanuele.azzoni@unimib.it (E.A.),
marella.debruijn@imm.ox.ac.uk (M.F.T.R.d.B.)

In brief

Azzoni et al. report that hematopoietic stem cell precursors (pro-HSC) still emerge in the absence of circulation, but are functionally impaired and do not activate the hematopoietic program. Single-cell transcriptomics indicate that the onset of circulation induces a metabolic switch that is required for hematopoietic development.

Highlights

- Hemogenic endothelium (HE) and pro-HSCs emerge in *Ncx1*$^{-/-}$ embryos lacking circulation
- *Ncx1*$^{-/-}$ pro-HSCs are transcriptionally and functionally impaired
- The HE to pro-HSC transition involves a glycolysis to OxPhos metabolic shift
- Activation of glycolysis reduces hematopoietic output of E9.5 intraembryonic precursors

Azzoni et al., 2021, Cell Reports *37*, 110103
December 14, 2021 © 2021 The Author(s).
https://doi.org/10.1016/j.celrep.2021.110103

CellPress

Cell Reports

CellPress
OPEN ACCESS

Article

The onset of circulation triggers a metabolic switch required for endothelial to hematopoietic transition

Emanuele Azzoni,[1,11,*] Vincent Frontera,[1] Giorgio Anselmi,[1] Christina Rode,[1] Chela James,[2] Elitza M. Deltcheva,[2] Atanasiu S. Demian,[3] John Brown,[2] Cristiana Barone,[4] Arianna Patelli,[4] Joe R. Harman,[1] Matthew Nicholls,[1] Simon J. Conway,[5] Edward Morrissey,[3] Sten Eirik W. Jacobsen,[1,6,7] Duncan B. Sparrow,[8] Adrian L. Harris,[9] Tariq Enver,[2,10] and Marella F.T.R. de Bruijn[1,12,*]

[1]MRC Molecular Hematology Unit, MRC Weatherall Institute of Molecular Medicine, Radcliffe Department of Medicine, University of Oxford, Oxford, OX3 9DS, UK
[2]Department of Cancer Biology, UCL Cancer Institute, University College London, London, WC1E 6DD, UK
[3]MRC WIMM Centre for Computational Biology, MRC Weatherall Institute of Molecular Medicine, Radcliffe Department of Medicine, University of Oxford, Oxford, OX3 9DS, UK
[4]School of Medicine and Surgery, University of Milano-Bicocca, Monza, 20900, Italy
[5]HB Wells Center for Pediatric Research, Indiana University School of Medicine, Indianapolis, Indiana, IN 46033, USA
[6]Hematopoietic Stem Cell Laboratory, MRC Weatherall Institute of Molecular Medicine, Radcliffe Department of Medicine, University of Oxford, Oxford, OX3 9DS, UK
[7]Department of Cell and Molecular Biology, Wallenberg Institute for Regenerative Medicine and Department of Medicine, Center for Hematology and Regenerative Medicine, Karolinska Institutet and Karolinska University Hospital, 171 77 Stockholm, Sweden
[8]Department of Physiology, Anatomy and Genetics, BHF Centre of Research Excellence, University of Oxford, Oxford, OX1 3PT, UK
[9]Department of Oncology, Molecular Oncology Laboratories, MRC Weatherall Institute of Molecular Medicine, University of Oxford, John Radcliffe Hospital, Oxford, OX3 9DS, UK
[10]Division of Molecular Medicine and Gene Therapy, Lund University, Lund, 22184, Sweden
[11]Present address: School of Medicine and Surgery, University of Milano-Bicocca, Monza, 20900, Italy
[12]Lead contact
*Correspondence: emanuele.azzoni@unimib.it (E.A.), marella.debruijn@imm.ox.ac.uk (M.F.T.R.d.B.)
https://doi.org/10.1016/j.celrep.2021.110103

SUMMARY

Hematopoietic stem cells (HSCs) emerge during development from the vascular wall of the main embryonic arteries. The onset of circulation triggers several processes that provide critical external factors for HSC generation. Nevertheless, it is not fully understood how and when the onset of circulation affects HSC emergence. Here we show that in *Ncx1*$^{-/-}$ mouse embryos devoid of circulation the HSC lineage develops until the phenotypic pro-HSC stage. However, these cells reside in an abnormal microenvironment, fail to activate the hematopoietic program downstream of Runx1, and are functionally impaired. Single-cell transcriptomics shows that during the endothelial-to-hematopoietic transition, *Ncx1*$^{-/-}$ cells fail to undergo a glycolysis to oxidative phosphorylation metabolic switch present in wild-type cells. Interestingly, experimental activation of glycolysis results in decreased intraembryonic hematopoiesis. Our results suggest that the onset of circulation triggers metabolic changes that allow HSC generation to proceed.

INTRODUCTION

Hematopoietic stem cells (HSCs) have the capacity to maintain all blood cell lineages during adult life. In the vertebrate embryo they are first and autonomously generated from a specialized subset of arterial endothelial cells, the hemogenic endothelium (HE), through a endothelial-to-hematopoietic transition (EHT) (de Bruijn et al., 2000; Ivanovs et al., 2011; Medvinsky and Dzierzak, 1996; Bertrand et al., 2010; Boisset et al., 2010; Kissa and Herbomel, 2010). The transcription factor *Runx1* is critically required for, and expressed during EHT starting from the HE (reviewed in (de Bruijn and Dzierzak, 2017). The HSC lineage diverges from the HE with the emergence of pro-HSC at E9.5, which develop via pre-HSC type I and II into fully functional definitive HSCs at E11.5 (Rybtsov et al., 2014; Rybtsov et al., 2011; Taoudi et al., 2008). This places the specification of the HSC lineage around E9.5 (Rybtsov et al., 2014; Swiers et al., 2013), a time point that coincides with major changes in the developing mouse embryo, including the establishment of unidirectional blood flow (McGrath et al., 2003). Indeed, the onset of circulation is an important extrinsic regulator of blood cell generation *in vitro* and *in vivo* (Adamo et al., 2009; Lundin et al., 2020; North et al., 2009).

Circulation can affect HSC development in multiple ways. Shear stress sensing in the endothelium increases extracellular adenosine levels, which through the cAMP-PKA-CREB axis

This is an open access article under the CC BY license (http://creativecommons.org/licenses/by/4.0/).

modulate the expression of several hematopoietic related genes, such as CXCL8- and BMP-related genes (Jing et al., 2015) (Kim et al., 2015). The cAMP-PKA-CREB axis can alternatively be stimulated by prostaglandin E2, also controlled by blood flow (Diaz et al., 2015). Blood flow is also associated with nitric oxide signaling (Adamo et al., 2009; North et al., 2009; Wang et al., 2011) and the modulation of several signaling pathways important for HSCs. Indeed, *ex vivo* induction of shear stress in aorta-gonad-mesonephros (AGM)-derived cells upregulates hematopoietic genes along with prostaglandin, Wnt, and Notch genes (Diaz et al., 2015). Finally, cyclic stretch associated with blood flow activates Rho-Yap mechanotransduction, promoting hematopoietic stem and progenitor cell (HSPC) production *in vitro* and *in vivo* (Lundin et al., 2020). It is unclear whether circulation induces the initial specification of the mammalian HSC lineage from HE, or acts at later stages on specified HSC precursors to induce their maturation into functional HSCs and/or downstream progeny (Adamo et al., 2009; North et al., 2009; Wang et al., 2011). Indeed, direct analysis of emerging HSCs in mice devoid of circulation has been hampered by their lethality prior to the emergence of transplantable definitive HSCs (Jones et al., 2008; Wakimoto et al., 2001; Wakimoto et al., 2000; Xu et al., 2011).

Here, we investigated the earliest steps of HSC lineage specification in the *Ncx1*$^{-/-}$ embryo that lacks the heart-specific Na^{2+}/Ca^{2+} exchanger gene *Slc8a1* and fails to initiate a heartbeat and circulation (Koushik et al., 2001; Wakimoto et al., 2001; Wakimoto et al., 2000). Crossing this mouse line with our *Runx1* +23 enhancer-reporter line (23GFP) (Bee et al., 2010; Swiers et al., 2013) allowed assessment and isolation of cells undergoing EHT in the absence of circulation. We found that in *Ncx1*$^{-/-}$ embryos, phenotypic HE and pro-HSC did emerge, but failed to upregulate key hematopoietic genes and did not mature into functional HSCs upon *ex vivo* culture. Single-cell transcriptomics showed that the *Ncx1*$^{-/-}$ HSC lineage was specified in an aberrant microenvironment enriched in hematopoiesis-inhibitory signals, and analysis of the EHT trajectory placed the block at the level of the HE to pro-HSC transition. Differential gene expression analysis implicated metabolic changes in the process. Interestingly, progression through EHT in the wild-type was associated with a downregulation of glycolysis, while *Ncx1*$^{-/-}$ cells showed a hypoxia-mediated upregulation of glycolysis genes. Through *in vivo* and *ex vivo* perturbation of glycolysis, we demonstrated that this metabolic switch is required for normal differentiation of intraembryonic hematopoietic progenitors. Our findings suggest that metabolic changes associated with the onset of circulation promote HSC development and bear relevance for the improvement of current protocols aimed at generating HSCs *in vitro*.

RESULTS

Phenotypic pro-HSCs emerge in the main arteries of *Ncx1*$^{-/-}$ embryos

To assess whether the HSC lineage is specified normally in the absence of circulation, we examined the emergence of HE and pro-HSCs in *Ncx1*$^{-/-}$ embryos. Runx1 expression marks HE and emerging HSPCs in the developing embryo (de Bruijn and Dzierzak, 2017; North et al., 1999; North et al., 2002). At E8.25 (4-7sp), the time point at which circulation commences (Ji et al., 2003; Lucitti et al., 2007; McGrath et al., 2003), Runx1 was detected in just a few cells of the wild-type posterior paired dorsal aortae (Figure 1A). A similar pattern of Runx1 expression was seen in *Ncx1*$^{-/-}$ embryos, which at this point show normal vascular morphology and expression of the arterial marker Dll4 (Figure 1A). By E9.5, Runx1 expression had extended along the dorsal aorta in the AGM region and in the proximal parts of the vitelline and umbilical arteries of both wild-type and *Ncx1*$^{-/-}$ embryos (Figure 1B). In spite of severe vascular defects in *Ncx1*$^{-/-}$ embryos (Hwa et al., 2017), Dll4 expression was still detected (Figures 1A and 1C), in line with previous reports that blood flow does not influence the expression of early arterial markers (Chong et al., 2011). To quantify HE, the *Ncx1*$^{-/-}$ line was crossed with the 23GFP transgenic reporter mouse line in which the *Runx1* +23 enhancer mediates expression of a GFP reporter to all cells undergoing EHT, including pre-HE and HE (Bee et al., 2010; Swiers et al., 2013). At E8.25, Ter119$^-$ CD45$^-$ VE-Cadherin$^+$ CD41$^-$ 23GFP$^+$ pre-HE was present at normal frequency in *Ncx1*$^{-/-}$ embryos (Figures 1D, 1E, and S1A). At E9.5, Ter119$^-$ VE-Cadherin$^+$ CD45$^-$ CD43$^-$ CD41$^-$ 23GFP$^+$ HE and Ter119$^-$ CD45$^-$ CD43$^-$ VE-Cadherin$^+$ CD41^{low} pro-HSCs (also expressing the 23GFP transgene) were present at a normal (HE) or increased (pro-HSC) frequency in the caudal part (CP) of *Ncx1*$^{-/-}$ embryos (Figures 1F and 1G) though in decreased numbers, in line with the overall reduced size of the mutant embryo at this developmental time (Figures S1B and S1C). These data indicate that phenotypic HE and pro-HSC are generated in the absence of circulation.

Ncx1$^{-/-}$ pro-HSCs lack markers of active hematopoietic commitment

Immunofluorescence confirmed the presence of cells with phenotypes consistent with HE and pro-HSCs in the dorsal aorta and vitelline artery of wild-type and *Ncx1*$^{-/-}$ embryos (Figure 2A). Transcript analysis of flow sorted pre-HE and nonhemogenic endothelium (Figure S2A) at the mini-bulk level (25 cells/sample) did not show widespread significant changes in endothelial and/or hematopoietic-associated genes in E8.25 *Ncx1*$^{-/-}$ versus wild-type samples (Figure S2C). By E9.5, *Ncx1*$^{-/-}$ HE showed decreased *Gata2* expression, which continued into the pro-HSC population where *Tal1* was also decreased and the endothelial marker *Kdr* was increased. A trend in decreased *Runx1, Gfi1*, and *Myb* expression was also seen (Figures S2B and S2C). Single-cell qRT-PCR on HE and Pro-HSCs isolated from E9.5 *Ncx1*$^{-/-}$ and wild-type CPs confirmed the decreased *Tal1, Lmo2* expression in *Ncx1*$^{-/-}$ HE, and the decreased expression of *Gata2* and *Runx1* in pro-HSCs, along with a reduction in the Runx1 downstream targets *Gfi1* and *Myb* (Figures 2B–2D), indicative of a failure to establish the transcriptional program underlying hematopoietic commitment (Swiers et al., 2013). In line with this, analyses of E10.5 *Ncx1*$^{-/-}$ embryos showed no signs of progression along the HSC path, with a further reduction in pro-HSCs, and no detectable pre-HSC type I or II (Figure S2D). Taken together, our data show that in the absence of circulation, the emergence of Runx1$^+$/23GFP$^+$ pre-HE in the E8.25 CP was relatively unaffected. In contrast, E9.5 HE and pro-HSCs, while phenotypically detectable, showed transcriptional changes culminating in a

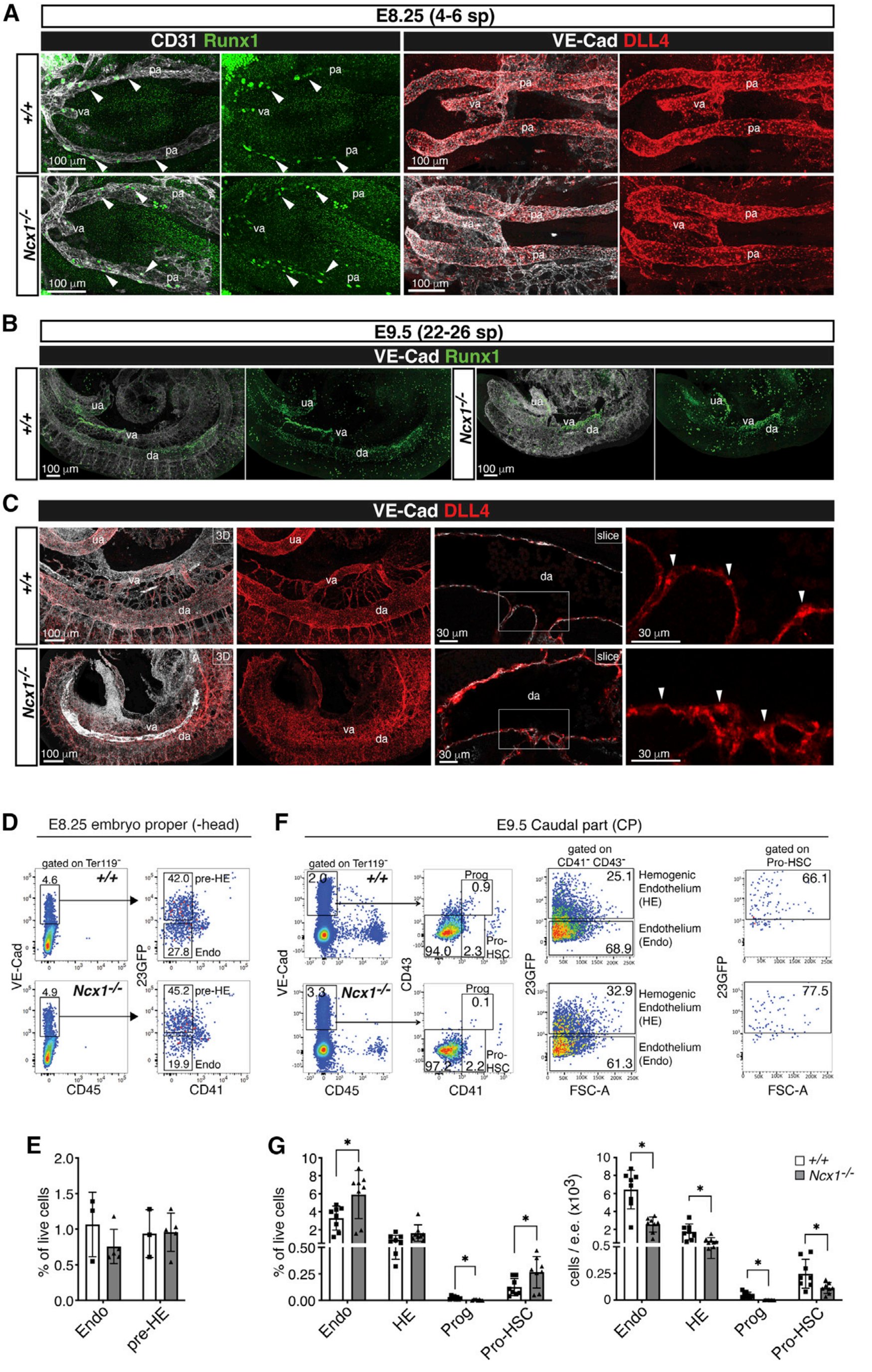

Figure 1. Phenotypically defined pro-HSCs are found in *Ncx1*$^{-/-}$ E9.5 embryos

(A) Confocal whole mount immunofluorescence (WM-IF) of E8.25 (4-6sp) wild-type (+/+) and *Ncx1*$^{-/-}$ embryos. Images show maximum intensity projections. Left panel: Arrowheads indicate VE-Cadherin^{+} Runx1^{+} cells in the paired aortae (pa) or vitelline artery (va). N = 6 (+/+), N = 7 (*Ncx1*$^{-/-}$) embryos analyzed. Right panel: N = 6 (+/+), N = 6 (*Ncx1*$^{-/-}$) embryos analyzed. Scale bars: 100 μm.

(B) Confocal WM-IF analysis of E9.5 (22-26sp) +/+ and *Ncx1*$^{-/-}$ embryos (maximum intensity projections). N = 6 (+/+), N = 8 (*Ncx1*$^{-/-}$) embryos analyzed. da: dorsal aorta; va: vitelline artery; ua:umbilical artery. Scale bars: 100 μm.

(C) Confocal WM-IF analysis of E9.5 (22-26sp) +/+ and *Ncx1*$^{-/-}$ embryos. Arrowheads indicate examples of Dll4-expressing aortic endothelial cells. N = 3 (+/+), N = 3 (*Ncx1*$^{-/-}$) embryos analyzed. Scale bars: 100 μm (3D), 30 μm (slice).

(D) Flow cytometric analysis of E8.25-E8.5 (3-11sp) +/+ and *Ncx1*$^{-/-}$ embryos. Embryos of the same genotype were pooled. Data are representative of 4 independent experiments of N = 3 (+/+), N = 5 (*Ncx1*$^{-/-}$) samples of a total of 11 (+/+), 14 (*Ncx1*$^{-/-}$) embryos. Endo: Ter119^{-} VE-Cad^{+} CD45^{-} CD41^{-} 23GFP^{-} endothelium; HE: Ter119^{-} VE-Cad^{+} CD45^{-} CD41^{-} 23GFP^{+}.

(E) Graph showing quantification of flow cytometric analysis in (D). Data are mean ± standard deviation (SD).

(F) Flow cytometric analysis of E9.5 (21-26sp) +/+ and *Ncx1*$^{-/-}$ embryos. Embryos of the same genotype were pooled. Data representative of 4 independent experiments with N = 8 (+/+), N = 8 (*Ncx1*$^{-/-}$) samples of a total of 26 (+/+), 24 (*Ncx1*$^{-/-}$) embryos. Endo: Ter119^{-} VE-Cad^{+} CD45^{-} CD43^{-} CD41^{-} 23GFP^{-} endothelium; HE: Ter119^{-} VE-Cad^{+} CD45^{-} CD43^{-} CD41^{-} 23GFP^{+}; Prog: Ter119^{-} VE-Cad^{+} CD45^{-} CD43^{+} CD41^{+} progenitor cells; Pro-HSC: Ter119^{-} VE-Cad^{+} CD45^{-} CD43^{-} CD41^{low}.

(G) Graphs showing quantification of flow cytometric analysis in (F). e.e.: embryo equivalent. Data are mean ± SD.

failure to initiate the hematopoietic program downstream *Gata2* and *Runx1* and did not mature into pre-HSCs *in vivo*.

Lack of circulation results in abnormal Jag1-mediated Notch signaling in HE and pro-HSC

The Notch pathway is known to play a critical role in HSC emergence (Kumano et al., 2003; Robert-Moreno et al., 2005; Robert-Moreno et al., 2008; Souilhol et al., 2016b) and to respond directly to changes in circulation (Fang et al., 2017; Mack et al., 2017). The genes coding for the Notch receptors Notch1 and Notch4 and the ligands Dll4, Jag1, and Jag2 were previously detected in the E9.5 aortic wall (Robert-Moreno et al., 2005). Using single-cell and mini-bulk qRT-PCR we consistently saw a reduced expression of *Jag1*, *Jag2* and *Hey1*, *Hey2* in sorted *Ncx1*$^{-/-}$ HE and pro-HSCs, while *Dll4* and *Hes1* were not decreased (Figures 2C, E2 and S3A). This was particularly evident in the cell clusters showing higher *Runx1* expression (boxes in Figures 2C and 2D). The downregulation of Jag1 ligand and relatively unperturbed expression of Dll4 was confirmed at the protein level (Figure 2F, 1A, and 1C). In line with the latter, Notch intracellular domain (NICD), a mark of active Notch signaling, was still detected in the nuclei of 23GFP^{+} EHT cells in the E9.5 *Ncx1*$^{-/-}$ aorta (Figure 2G). In summary, these data indicate that lack of circulation impairs

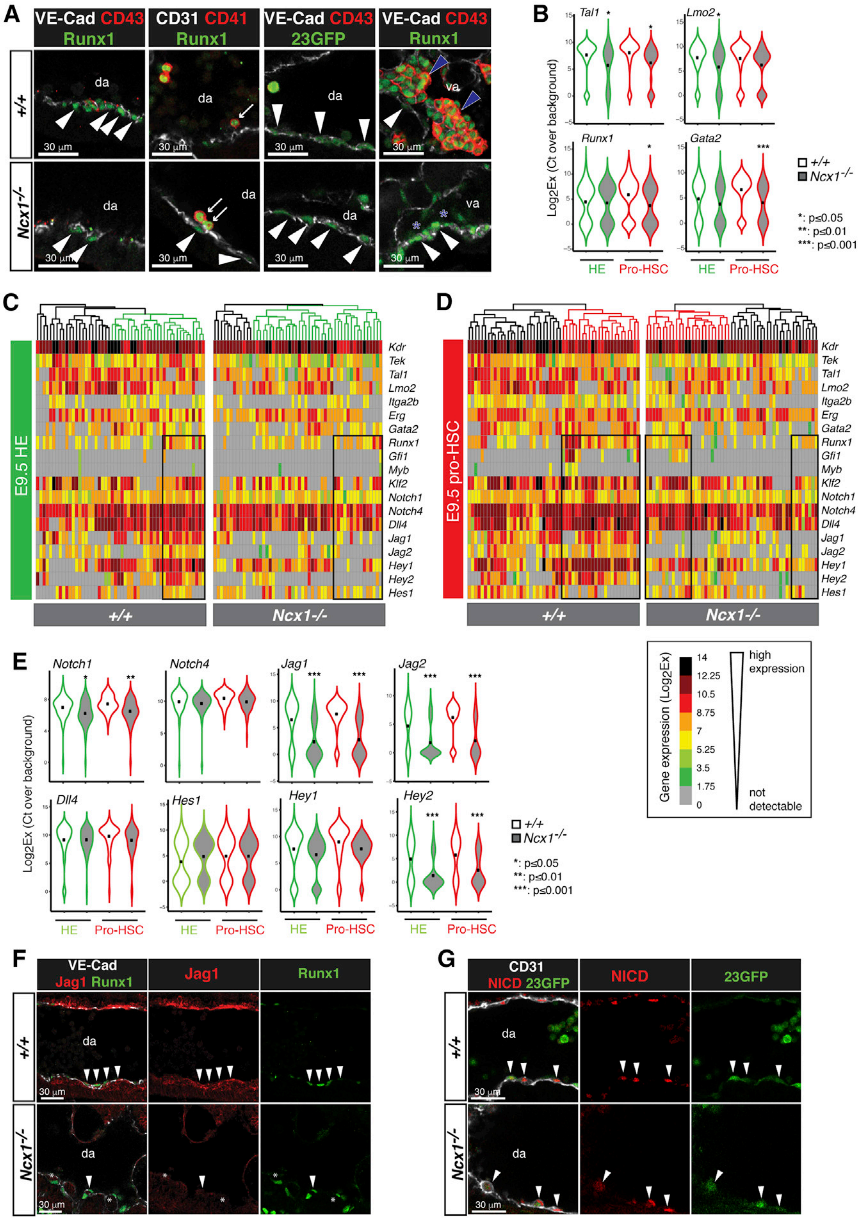

Figure 2. *Ncx1*$^{-/-}$ pro-HSCs downregulate key hematopoietic genes

(A) Confocal whole mount immunofluorescence analysis (WM-IF) of E9.5 (21-26sp) wild-type (+/+) and *Ncx1*$^{-/-}$ embryos. All panels show single 2.5 μm-thick optical slices representative of (left) N = 3 (+/+), N = 2 (*Ncx1*$^{-/-}$) embryos analyzed, (middle left) N = 2 (+/+), N = 2 (*Ncx1*$^{-/-}$) embryos, (middle right) N = 4 (+/+), N = 2 (*Ncx1*$^{-/-}$) embryos, and (right) N = 3 (+/+), N = 2 (*Ncx1*$^{-/-}$) embryos. Scale bars: 30 μm. Arrowheads indicate VE-Cad^{+} Runx1^{+}/23GFP^{+} CD43^{-} HE or CD31^{+} Runx1^{+} HE. Arrows indicate examples of CD41^{+} Runx1^{+} hematopoietic cells. Blue arrowheads indicate examples of VE-Cad^{+} CD43^{+} Runx1^{+} hematopoietic cells in wild-type embryos; blue asterisks highlight the absence of CD43^{+} hematopoietic cluster cells in *Ncx1*$^{-/-}$ embryos. da: dorsal aorta; va: vitelline artery.

(B) Multiplexed single cell qRT-PCR analysis of HE (Ter119^{-} VE-Cad^{+} CD45^{-} CD41^{-} CD43^{-} 23GFP^{+}) and pro-HSCs (Ter119^{-} VE-Cad^{+} CD45^{-} CD41^{low} CD43^{-} 23GFP^{+}), isolated from E9.5 embryos (22-26sp). Sort gates as in Figure S2B. Samples from two independent experiments (5 +/+, 10 *Ncx1*$^{-/-}$ embryos total) with 52 +/+ and *Ncx1*$^{-/-}$ HE and 53 +/+ and *Ncx1*$^{-/-}$ pro-HSCs analyzed. Violin plots represent the expression of selected genes; black dots indicate average values. *p ≤ 0.05; **p ≤ 0.01; ***p ≤ 0.001.

(C and D) Clustered heatmaps showing multiplex single cell qRT-PCR analysis of E9.5 +/+ or *Ncx1*$^{-/-}$ HE (C) and pro-HSC (D). Columns represent single cells; rows represent genes. Column dendrograms are ordered using hierarchical clustering. Black boxes highlight groups of cells with high *Runx1* expression.

(E) Multiplexed single cell qRT-PCR analysis showing expression of selected genes as in (B). *p ≤ 0.05; **p ≤ 0.01; ***p ≤ 0.001.

(F) Confocal WM-IF of E9.5 (21-24sp) +/+ and *Ncx1*$^{-/-}$ embryos. Single 2.5 μm-thick slices are shown. N = 3 (+/+), N = 3 (*Ncx1*$^{-/-}$) embryos analyzed. Arrowheads indicate examples of VE-Cad^{+} Runx1^{+} Jag1^{+} cells. Asterisks highlight lack of Jag1 expression in *Ncx1*$^{-/-}$ embryos. Scale bars: 30 μm.

(G) Confocal WM-IF of E9.5 (23-25sp) wild-type (+/+) and *Ncx1*$^{-/-}$ embryos. Single 2.5 μm-thick slices are shown. N = 4 (+/+), N = 4 (*Ncx1*$^{-/-}$) embryos analyzed. Arrowheads indicate examples of CD31^{+} Runx1^{+} NICD^{+} cells. da: dorsal aorta. Scale bars: 30 μm.

Jag1-, but not Dll4-mediated Notch signaling in E9.5 HE and pro-HSCs.

The hematopoietic microenvironment is perturbed in *Ncx1*$^{-/-}$ embryos

Based on the results described above it was unlikely that the defect in the HSC lineage was due to a disruption of the arterial program. Indeed, other known HSC-generative niche components may be affected by the lack of circulation. Peri-aortic smooth muscle cells (SMCs) have been implicated in AGM hematopoiesis, although their role remains debated (Mirshekar-Syahkal et al., 2013; Richard et al., 2013) (). Strikingly, in E9.5 *Ncx1*$^{-/-}$ embryos, α−SMA^{+} cells were lost around the dorsal aorta (Figure 3A) and there was a decrease in the thickness of the subaortic mesenchyme (Figures 3A and 3B), another potential niche component (Richard et al., 2013). Also macrophages, known to play a role in the later HSC niche (Mariani et al., 2019; Travnickova et al., 2015), were decreased in frequency in the E9.5 *Ncx1*$^{-/-}$ versus wild-type CP and were not seen underlying Runx1^{+} cells in the dorsal aorta (Figures 3C–3E). To begin to assess how changes in the cellular components of the hematopoietic niche might affect the signaling microenvironment, we performed

CellPress
OPEN ACCESS

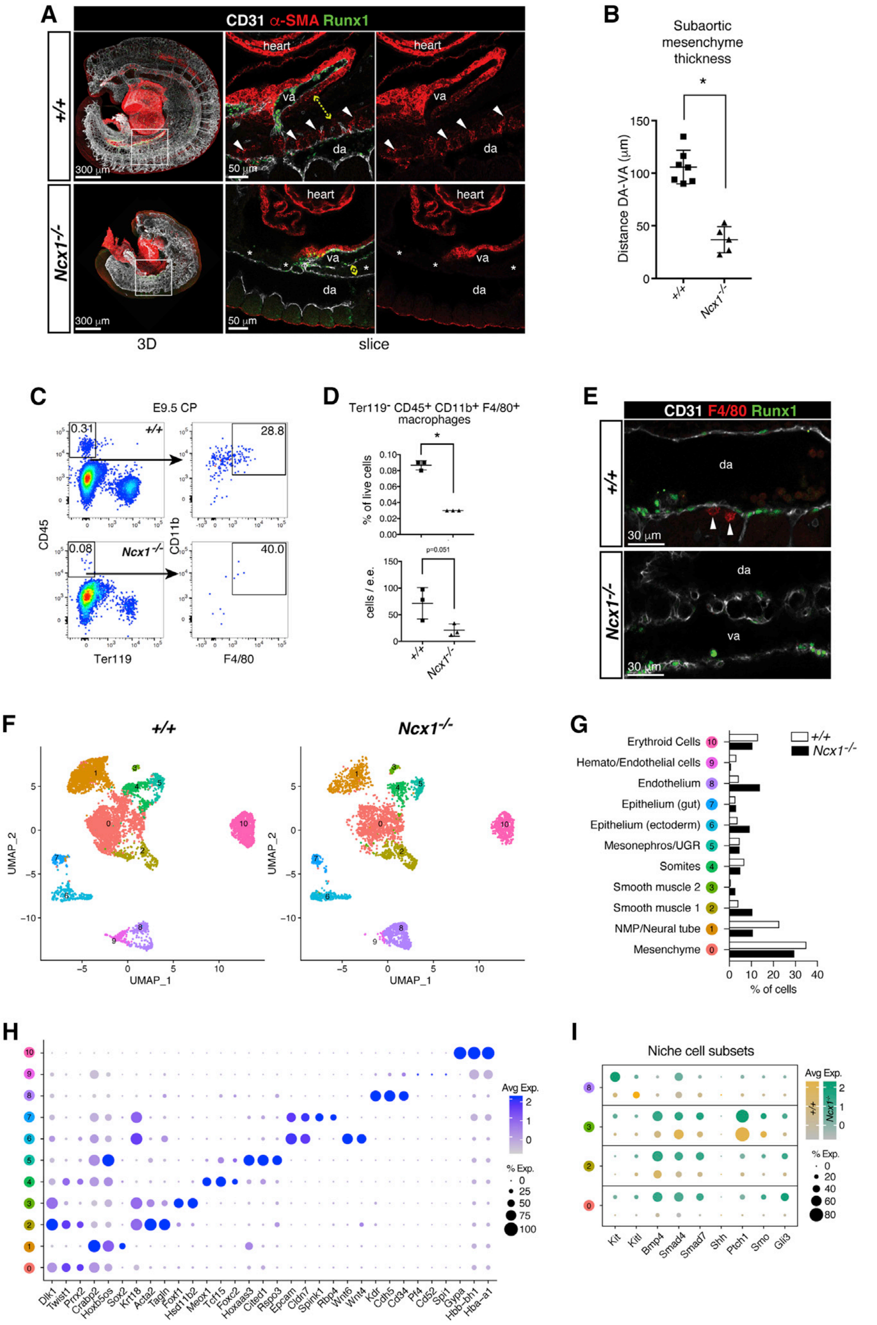

Figure 3. $Ncx1^{-/-}$ embryos display an aberrant peri-aortic microenvironment

(A) Confocal WM-IF analysis of E9.5 (22-25sp) +/+ and $Ncx1^{-/-}$ embryos. Left panels show maximum intensity projections. Boxed area is magnified in the middle and right panels (single 2.5 μm-thick slices). Arrowheads indicate α-SMA^{+} peri-aortic SMCs, absent from $Ncx1^{-/-}$ embryos (asterisks). Yellow dashed arrow: distance between dorsal aorta (da) and vitelline artery (va). N = 3 (+/+), N = 3 ($Ncx1^{-/-}$) embryos analyzed. Scale bars: 300 μm (3D), 50 μm (slice).

(B) Distance between dorsal aorta and vitelline artery as a measurement of the sub-aortic mesenchyme thickness. Measurements done on images from N = 7 (+/+), N = 5 ($Ncx1^{-/-}$) different embryos (1-4 images/embryo; 5 measurements / image; 16 (+/+), 17 ($Ncx1^{-/-}$) different images used. Data are mean ± SD.

(C) Flow cytometric analysis of macrophages (Ter119^{-} CD45^{+} F4/80^{+} CD11b^{+}) in E9.5 (21-25sp) +/+ and $Ncx1^{-/-}$ caudal part (CP). N = 3 (+/+), N = 3 ($Ncx1^{-/-}$) embryos were analyzed individually in 2 independent experiments.

(D) Quantification of flow cytometric analysis in (C). Data are mean ± SD.

(E) Confocal WM-IF of E9.5 (21-24sp) +/+ and $Ncx1^{-/-}$ embryos (single 2.5 μm-thick slice representative of N = 4 (+/+), N = 4 ($Ncx1^{-/-}$) embryos). Arrowheads: peri-aortic F4/80^{+} macrophages. Scale bars: 30 μm.

(F) Uniform Manifold Approximation and Projection (UMAP; (Becht et al., 2018)) of the E9.5 (20-23sp) +/+ and $Ncx1^{-/-}$ PAS scRNA-Seq dataset. Cells were isolated from 4 embryos for each genotype.

(G) Percentage of cells in each PAS scRNA-Seq cluster.

(H) Bubble plot showing marker genes for each PAS scRNA-Seq cluster. Dot size indicates the percentage of expressing cells; color intensity indicates expression level.

(I) Bubble plot showing expression of genes encoding for hematopoietic niche signals in niche cell subsets. Expression is shown separately for +/+ and $Ncx1^{-/-}$ cells.

single-cell RNA sequencing (scRNA-seq; 10x Genomics) on E9.5 wild-type and $Ncx1^{-/-}$ CP. Cells clustered in 11 populations, the identity of which was determined based on expression of marker genes (Figures 3F–3H). While the observed reduction in $Ncx1^{-/-}$ mesenchyme was reflected in the scRNA-seq dataset, the loss of aortic SMCs was not captured transcriptionally in the absence of spatial information. This is consistent with αSMA^{+} SMCs being present around the $Ncx1^{-/-}$ vitelline and umbilical arteries, and with the relative increase in endothelial cells (Figures 3A and 3G). Macrophages were not detected in the scRNA-seq dataset. Analysis of signals implicated in the HSC-generative niche showed that the pro-hematopoietic factor Kit ligand (Kitl) (Azzoni et al., 2018) was downregulated in $Ncx1^{-/-}$ endothelial cells. In contrast, BMP4, Smad4, and Smad7, members of the Tgfβ pathway exerting an inhibitory effect on EHT (Souilhol et al., 2016a; Vargel et al., 2016), were increased in $Ncx1^{-/-}$ mesenchyme and SMCs (Figure 3I). The Hedgehog pathway genes Ptch1, Smo, and Gli3, also involved in EHT (Crisan et al., 2016; Souilhol et al., 2016a), were upregulated in the mutant (Figure 3I). These results show that in the absence of circulation, the intricate balance of signals in the HSC-generative niche was perturbed. Establishing precisely which cellular components and signals are critical in this context awaits a more detailed analysis of the HSC-generative niche in general, as this is still poorly understood.

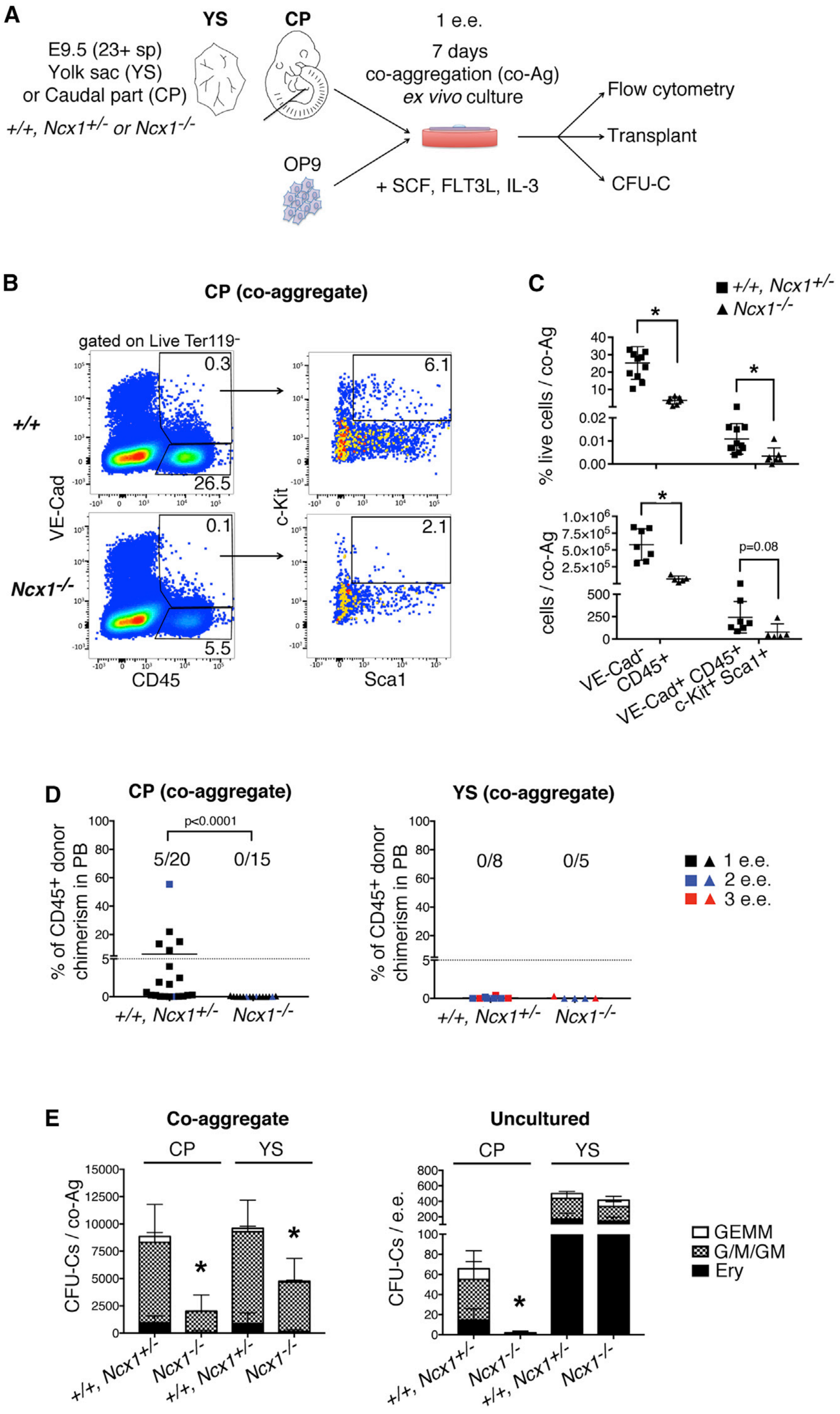

Figure 4. *Ncx1*$^{-/-}$ pro-HSCs fail to mature into functional HSCs *ex vivo*

(A) Schematic of OP9 co-aggregate culture experiments.

(B) Flow cytometric analysis of OP9 co-aggregates with CP of E9.5 (23-28sp) control (+/+ or *Ncx1*$^{+/-}$) or *Ncx1*$^{-/-}$ embryos. Co-aggregates were analyzed individually. N = 11 (+/+ or *Ncx1*$^{+/-}$), N = 7 (*Ncx1*$^{-/-}$); 4 independent experiments.

(C) Quantification of flow cytometric analysis in (B). Data are mean ± SD.

(D) Repopulation analysis of irradiated CD45.1 syngeneic mice transplanted with 1-3 e.e. of E9.5 (21-27sp) control (+/+ or *Ncx1*$^{+/-}$) or *Ncx1*$^{-/-}$ CD45.2$^+$ CP (left) or YS (right) cells after culture. Graphs shows peripheral blood (PB) chimerism represented as % donor cells (CD45.2$^+$) among total CD45$^+$ cells, 16 weeks after transplant. Data from 5 independent experiments. Lines show the mean.

(E) CFU-C per embryo equivalent (e.e.) of control (+/+ or *Ncx1*$^{+/-}$) or *Ncx1*$^{-/-}$ E9.5 (21-27sp) CP and YS, after culture (left) or uncultured (right). N = 5 (+/+ or *Ncx1*$^{+/-}$), N = 5 (*Ncx1*$^{-/-}$) from 3 independent experiments (co-aggregate). N = 6 (+/+ or *Ncx1*$^{+/-}$), N = 7 (*Ncx1*$^{-/-}$) CP; N = 6 (+/+ or *Ncx1*$^{+/-}$), N = 6 (*Ncx1*$^{-/-}$) YS from 2 independent experiments (uncultured). GEMM: granulocyte, erythroid, monocyte/macrophage, megakaryocyte; G/M/GM: granulocyte, monocyte/macrophage; Ery: erythroid. Data are mean ± SD.

Ncx1$^{-/-}$ pro-HSC do not develop into functional HSCs *ex vivo*

We next asked whether *Ncx1*$^{-/-}$ pro-HSCs mature into functional HSCs when placed in a conducive environment *ex vivo*. The OP9 co-aggregation culture system (Figure 4A) supports the maturation of pro-HSCs into definitive HSCs, even without endogenous cellular niche components (Batsivari et al., 2017; Rybtsov et al., 2014). Of note, OP9 stromal cells express Jag1 (Huang et al., 2013) (Figure S3B) and thus might compensate for the decreased Jag1 in *Ncx1*$^{-/-}$ cells. Similar to later stages (Souilhol et al., 2016b), Notch signaling was required for the maturation of HSCs, but not CFU-Cs, in E9.5 co-aggregate cultures (Figures S3C–S3G). Within *Ncx1*$^{-/-}$ CP co-aggregates, total hematopoietic output (VE-Cadherin$^+$ CD45$^+$) was decreased in frequency and numbers compared to wild-type, with phenotypic HSC/progenitor cells (Ter119$^-$ VE-Cadherin$^+$ CD45$^+$ c-Kit$^+$ Sca-1$^+$) significantly reduced in frequency (Figures 4B and 4C). This was corroborated at the functional level, with co-aggregates of E9.5 *Ncx1*$^{-/-}$ CPs lacking short- and long-term hematopoietic reconstitution potential upon transplantation into adult irradiated recipients (Figures 4D, S4A, S4B, S4C, S4D, and S4E). Interestingly, *Ncx1*$^{-/-}$ cultures did generate some hematopoietic progenitor cells, though notably no CFU-GEMM and very few CFU-E; virtually no CFU-C were detected in the freshly isolated CP (Figure 4E). Control CP co-aggregates did support the generation of HSCs with long-term multilineage

reconstitution and also generated robust numbers of CFU-C (Figures 4D, 4E, S4A, S4B, S4C, S4D, and S4E). Co-aggregates of *Ncx1*$^{-/-}$ YS showed near normal phenotypic hematopoietic output (Figures S4F–S4H), however, no functional HSC activity was detected in line with the absence of HSC potential in control YS co-aggregates (Figures S4A and 4D; and previous reports Ganuza et al., 2018; Rybtsov et al., 2014). CFU-C generation was decreased by half in *Ncx1*$^{-/-}$ YS co-aggregates compared to control. This was in contrast to the uncultured *Ncx1*$^{-/-}$ YS, which as described (Lux et al., 2008) had normal numbers of CFU-C (Figure 4E) and erythro-myeloid progenitors (EMP; Figure S1D), suggesting that circulation affects the self-renewal potential of YS CFU-C. In summary, our data demonstrate that phenotypic *Ncx1*$^{-/-}$ pro-HSCs are unable to mature into functional, transplantable HSCs when placed in a supportive environment *ex vivo*, suggesting they are intrinsically impaired. Moreover, the persistence of the defect despite the presence of an exogenous Jag1 signal in culture suggests that the Jag1 downregulation in *Ncx1*$^{-/-}$ HE and pro-HSC does not fully explain the phenotype, and that there are other factors at play.

Single cell transcriptomics identifies dysregulated metabolic pathways in HE and pro-HSC in the absence of circulation

To explore the defects in the HSC lineage in more detail we performed scRNA-seq (Smart-Seq2) on index-sorted E9.5 wild-type and *Ncx1*$^{-/-}$ 7-AAD$^-$ Ter119$^-$ VE-Cadherin$^+$ 23GFP$^+$ cells, consisting of a mix of pro-HSC, HE, and hematopoietic progenitors. To increase the number of pro-HSCs captured these were also sorted directly; non-hemogenic endothelial cells were isolated in parallel (Figure S5A). A total of 736 cells passed quality controls, evenly divided between the two genotypes and with an average of 8,045 genes detected per cell (Figures S5B and S5C). We identified six cell clusters in the full dataset (Figure 5A). Analysis of arterial (*Dll4*), venous (*Nr2f2*), and hematopoietic (*Runx1*) gene expression (Figure 5B), along with Ter119, VE-Cadherin, CD43, CD41, and CD45 surface marker expression (Figure S5D), showed that clusters 1 and 2 represent a mix of pro-HSCs, progenitors and arterial-associated HE from *Ncx1*$^{-/-}$ and wild-type embryos, respectively (Figure S5E). This was further corroborated by expression of CD44, recently reported as a marker for EHT (Oatley et al., 2020), in clusters 1 and 2 (Figure 5B). Clusters 3 and 4 contained both genotypes and showed high expression of the venous markers *Nr2f2* (Figure 5B) and *Aplnr* (Figure S5F) indicative of the presence of venous endothelium, most likely representing cells from the cardinal and vitelline veins and aberrant expression of venous markers in the mutant (Hwa et al., 2017). Cluster 6 contained mostly *Gata4*-positive *Ncx1*$^{-/-}$ cells (Figure S5F), likely representing heart cells included due to the disorganized vasculature. Clusters 1 and 2, representing the cells undergoing EHT, were selected for further analysis.

We identified 700 differentially expressed (DE) genes between *Ncx1*$^{-/-}$ and wild-type EHT cells (FDR < 0.1; 322 upregulated and 378 downregulated in cluster 1 versus 2; Table S1), including genes previously shown to respond to shear stress in hematopoietic precursor cells *in vitro* or *in vivo* (Diaz et al., 2015; Jing et al., 2015; Kim et al., 2015; North et al., 2009; Wang et al., 2011; Figure S5G). The top overrepresented Gene Ontology (GO) terms and KEGG pathways among genes upregulated in the *Ncx1*$^{-/-}$ cells were related to angiogenesis, glycolysis, and response to hypoxia/HIF-1 signaling (Figure 5C). Gene set enrichment analysis (GSEA) also identified hypoxia and glycolysis as the two most significant hallmarks in *Ncx1*$^{-/-}$ upregulated genes (Figure 5D). In addition, there was a downregulation of other metabolic pathways such as TCA and oxidative phosphorylation (OxPhos) in the *Ncx1*$^{-/-}$ (Figure S5H). A link between hypoxia and glycolysis has been well established in endothelial cells (reviewed in Li et al., 2019). A strong indicator of such a link in *Ncx1*$^{-/-}$ HE and pro-HSC was the overexpression of hypoxia-induced pyruvate dehydrogenase kinase 1 (*Pdk1*), as Pdk1 decreases OxPhos by limiting pyruvate entry into the TCA cycle (Kim et al., 2006; Figure S5H). Overall, these data point at metabolic changes driven by oxygen sensing taking place during EHT *in vivo*.

Pseudotime analysis shows an impaired HE to pro-HSC transition and a failure to downregulate glycolysis in *Ncx1*$^{-/-}$ cells undergoing EHT

We next analyzed the wild-type cells separately to infer the normal EHT trajectory. A diffusion map (Haghverdi et al., 2016) generated from the 2,000 most variable genes showed the wild-type trajectory, accompanied with increasing expression of arterial (*Dll4, Gja4, Vwf, Sox17*) and hemogenic/hematopoietic markers such as *CD44* (), *Foxc2* (Jang et al., 2015), *Runx1*, *Adgrg1* (Solaimani Kartalaei et al., 2015) and *Nupr1* (Zhu et al., 2020) (Figures 6A and S6A). Louvain clustering (Blondel et al., 2008) distinguished 13 clusters among the wild-type cells (Figure 6B), the identities of which were determined based on marker gene expression (Table S2). Clusters describing the EHT trajectory include the *Dll4*$^+$ *Cd44*^{lo} arterial endothelium (cluster 1), different stages of *Cd44*$^+$, *Foxc2*$^+$, *Sox17*$^+$, *Runx1*^{lo} HE (clusters 3, 7, 8, 13), and *Runx1*$^+$ pro-HSCs/progenitors (cluster 5). Next, we assessed where along the EHT trajectory the *Ncx1*$^{-/-}$ cells lie, by projecting them on the wild-type diffusion map using nearest neighbor regression. This showed that few *Ncx1*$^{-/-}$ cells completed the EHT trajectory, with most cells not reaching the *Cd44*$^+$, *Foxc2*$^+$, *Sox17*$^+$, *Runx1*^{lo} HE, and *Runx1*^{hi} *Adgrg1*$^+$ pro-HSC stages (compare Figures 6A and 6C).

To identify changes in gene expression associated with EHT, we fitted a pseudotime order to the Louvain clusters representing the wild-type EHT trajectory (clusters 1, 3, 13, 8, 7, 5) and the corresponding projected *Ncx1*$^{-/-}$ cells. The resulting diffusion pseudotime plot showed that *Ncx1*$^{-/-}$ cells accumulate in the middle part of the pseudotime trajectory (Figure 6D). *Cd44*, *Sox17*, *Itgb3*, and *Meis1* expression levels were comparable to wild-type, while only few *Ncx1*$^{-/-}$ cells expressed *Foxc2*, *Runx1, Nkx2-3*, and *Adgrg1*; *Gata2* expression was decreased over the entire *Ncx1*$^{-/-}$ trajectory, and *Vwf* toward the end (Figures 6E and S6B). DE genes were identified over two pseudotime intervals that, based on wild-type gene expression (Figure S6B) represent arterial endothelium [0, 0.4] and HE transiting to pro-HSCs [0.4, 0.8]. The top DE genes upregulated in the corresponding *Ncx1*$^{-/-}$ intervals were enriched for hypoxia-induced genes, including glycolysis, with focal adhesion enriched in the HE interval only (Figures S6C and S6D; Table S3). Interestingly, the hypoxia-induced gene *Adm* (Lanner et al., 2013) showed a

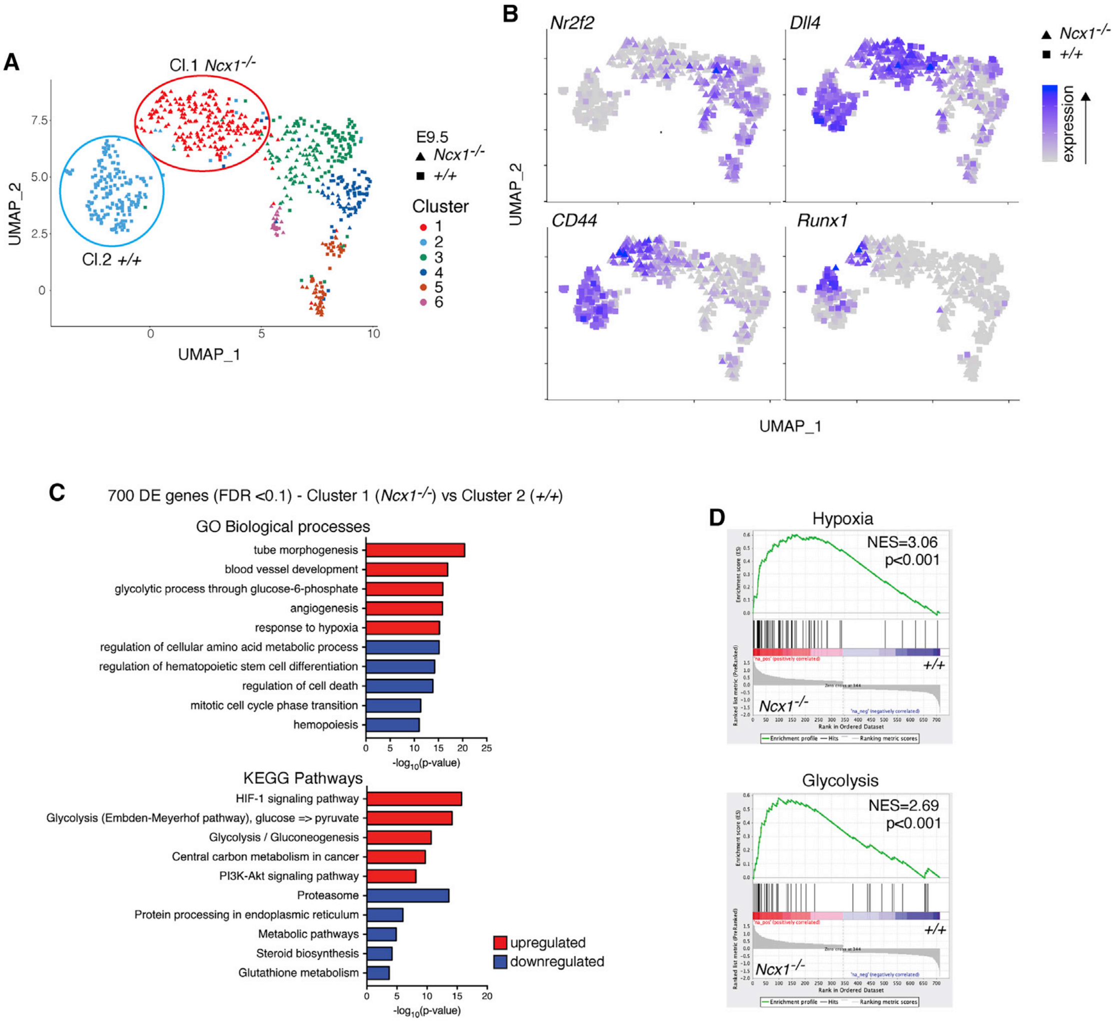

Figure 5. Single cell RNA-seq of wild-type and *Ncx1*$^{-/-}$ cells undergoing EHT

(A) UMAP of 736 EHT cells (362 wild-type and 374 *Ncx1*$^{-/-}$) analyzed by Smart-Seq2 scRNA-seq. Cl: cluster

(B) Expression of the venous marker *Nr2f2* (COUP-TFII), the arterial marker *Dll4,* the HE marker *CD44* and the master hematopoietic transcription factor *Runx1*, super-imposed on the UMAP.

(C) Gene Ontology (GO) biological processes and KEGG pathways enriched in upregulated (red bars) or downregulated (blue bars) DE genes between cluster 1 (*Ncx1*$^{-/-}$) and 2 (+/+).

(D) Gene set enrichment analysis (GSEA) of DE genes (FDR < 0.1) upregulated in cluster 1 compared to cluster 2. The top two gene sets are shown. NES: normalized enrichment score.

sharp downregulation over wild-type pseudotime, while it was strongly upregulated in the *Ncx1*$^{-/-}$ (Figure 6E). Along with expression of the genes coding for the Adm receptors, *Ramp2* and *Calcrl*, this could provide an autoregulatory loop that protects the *Ncx1*$^{-/-}$ hypoxic HE and pro-HSCs from apoptosis, as reported for other cell types (Oehler et al., 2001). As changes in metabolism have been associated with cell fate changes, we assessed metabolic gene expression over the EHT trajectory. Critical regulators of glycolysis such as *Pfkl*, *Pfkp*, *Pkm*, and others were significantly downregulated over pseudotime in wild-type, but not *Ncx1*$^{-/-}$ cells (Figures 6F, and S6E; Table S3). In contrast, the expression of several OxPhos genes increased along the wild-type EHT trajectory, but not in the *Ncx1*$^{-/-}$ (Table S3). Taken together, our data show that wild-type EHT is accompanied by a switch from glycolysis to OxPhos, while in the *Ncx1*$^{-/-}$ embryo, the observed upregulation of glycolysis is likely due to the severe hypoxia associated with a lack of circulation.

Experimental induction of glycolysis results in reduced hematopoietic output from intraembryonic EHT

To assess whether a glycolysis to OxPhos metabolic switch is required for hematopoietic cell generation, we employed *in vivo* and *ex vivo* perturbation approaches. Pregnant females were treated at E9.25 with dofetilide, a drug that induces bradycardia in embryos resulting in a transient block in circulation (Ritchie et al., 2015). Six hours after treatment we observed activation of hypoxia and glycolysis genes and the downregulation of

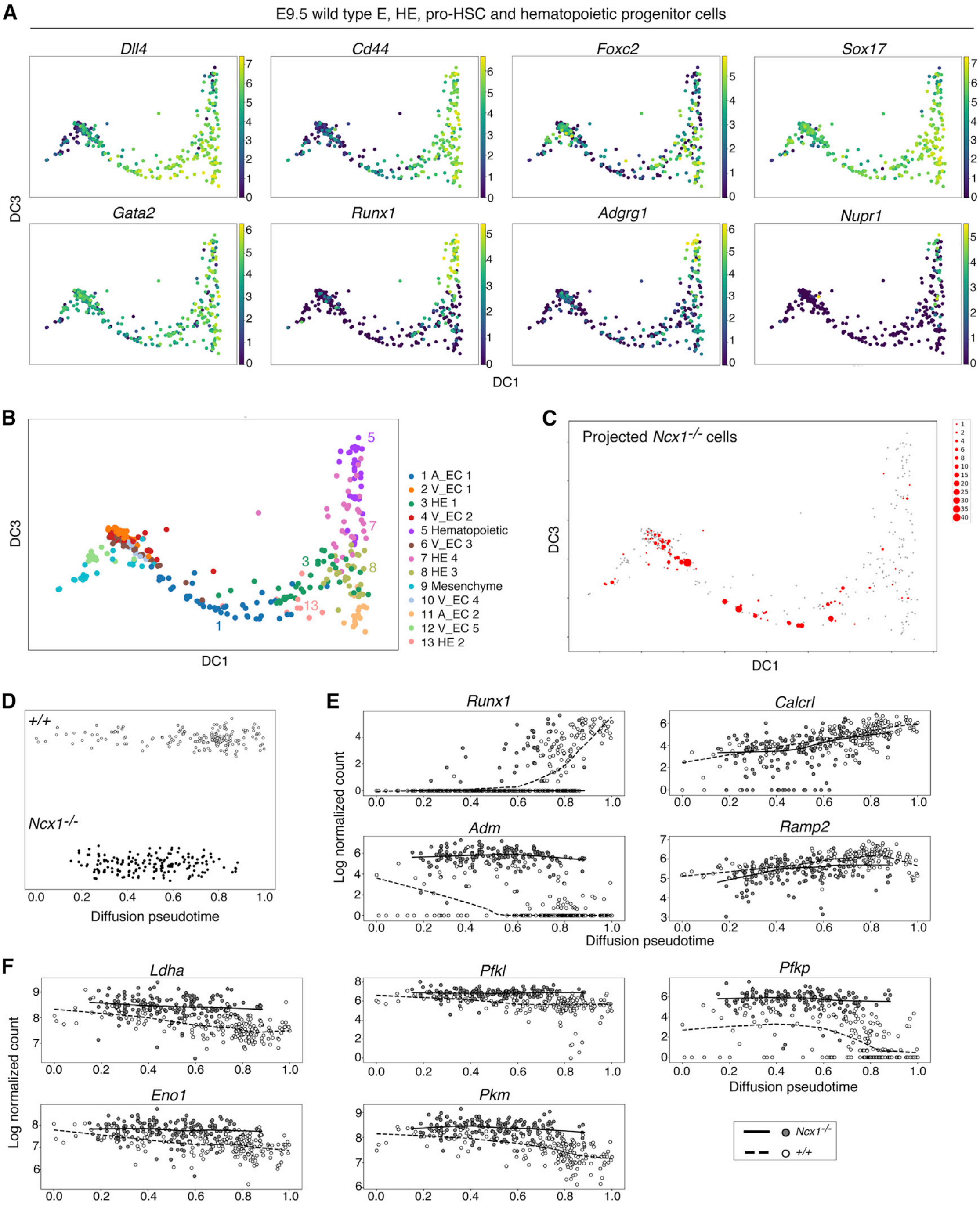

Figure 6. Mapping of *Ncx1*$^{-/-}$ cells on wild-type EHT diffusion trajectories shows their failure to develop past the *Cd44*$^{+}$ *Foxc2*$^{+}$ *Sox17*$^{+}$ *Runx1*^{lo} stage and to switch from glycolysis to OxPhos

(A) scRNA-seq diffusion maps of wild-type cells, showing expression of selected genes. Each dot represents an individual cell. Gene expression levels are shown as Log (normalized counts). DC: diffusion component.

(B) Diffusion map of wild-type cells indicating 13 Louvain clusters. Clusters were assigned to cell types based on gene expression (Table S2). Clusters 1, 3, 13, 8, 7, 5 were used to compute EHT trajectory. A: arterial; V: venous; EC: endothelial cells; HE: hemogenic endothelium.

(C) Projection of *Ncx1*$^{-/-}$ cells on the wild-type diffusion map, computed using the Nearest Neighbors regression algorithm. The size of the red dots shows the number of *Ncx1*$^{-/-}$ cells localizing to a particular point of the diffusion map.

(D) Beeswarm plots of wild-type and *Ncx1*$^{-/-}$ cells along the EHT differentiation trajectory, ordered by diffusion pseudotime. Each dot represents an individual cell.

(E and F). Scatterplots showing expression of selected genes along diffusion pseudotime. Genes in (E) are *Runx1*, the hypoxia responsive gene *Adm* and its receptors; Genes in (F) are genes involved in glycolysis. Gene expression is shown in the y axis as Log (normalized counts). Lines fitting the expression of genes over pseudotime were obtained by locally weighted linear regression.

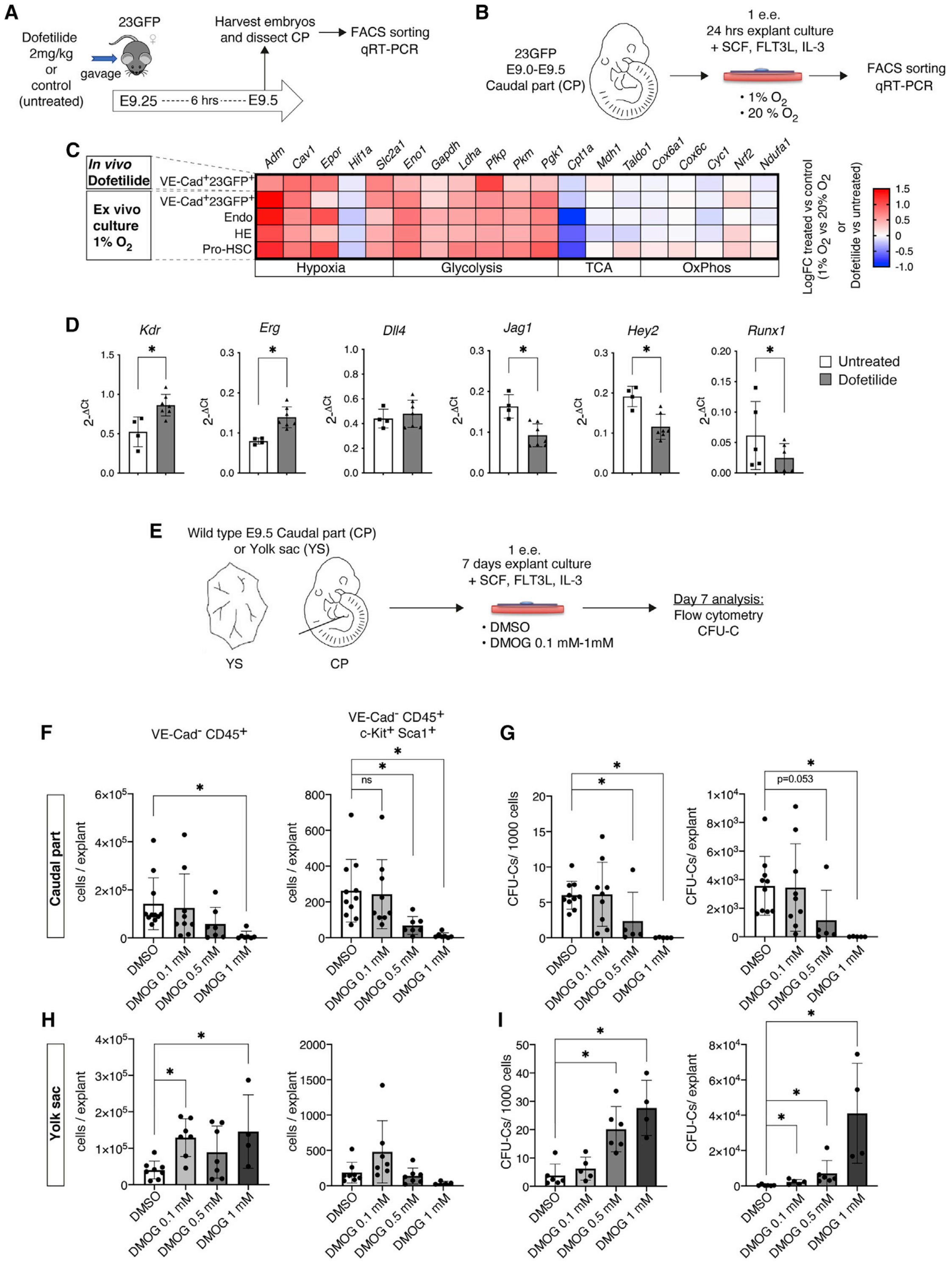

Figure 7. ***In vivo*** **and** ***in vitro*** **activation of hypoxia and glycolysis during EHT reduce the hematopoietic output**

(A) Schematic of dofetilide experiments.

(B) Schematic of explant cultures under hypoxia (1% O_2) or normoxia (20% O_2).

(C) Multiplexed mini-bulk qRT-PCR analysis on pools of 25 cells. Top row: data from Ter119$^-$ VE-Cad$^+$ 23GFP$^+$ cells isolated from E9.5 (21-27sp) embryos harvested from control and dofetilide-treated females. N = 5 (control), N = 6 (dofetilide) from 2 independent experiments. Bottom row: data from Ter119$^-$ VE-Cad$^+$ 23GFP$^+$ cells isolated from 13-24sp CP explants cultured for 24h under hypoxia (1% O_2). N = 5 (20% O_2), N = 5 (1% O_2), 4 independent experiments; samples from

(legend continued on next page)

OxPhos/TCA genes in VE-Cad+ 23GFP+ CP cells undergoing EHT (Figure 7A and 7C). These changes were comparable to the metabolic response seen after 24h explant culture of E9.0-E9.5 CPs under low oxygen (1% O_2) (Figures 7B and 7C), further supporting the efficacy of dofetilide treatment. Interestingly, the metabolic changes were accompanied by an upregulation of endothelial genes and downregulation of *Jag1*, *Hey2*, and *Runx1* (Figure 7D), similar to what was seen in *Ncx1*$^{-/-}$ embryos (Figures 2B, 2E, and S3A). Although no changes in VE-Cad+ 23GFP+ cell numbers were observed (Figure S7A), the transcriptional response indicates impaired activation of the hematopoietic program. As the transient effect of dofetilide and the extensive cell death observed in 1% O_2 cultures (Figure S7B) hampered functional analyses, we next performed E9.5 CP explant cultures in the presence of the pro-drug dimethyloxalylglycine (DMOG) (Figure 7E). The hydrolysis product of DMOG inhibits prolyl hydroxylases thereby stabilizing HIFs (Fraisl et al., 2009; Jaakkola et al., 2001), thus mimicking the transcriptional response to hypoxia. Explant cultures with increasing concentrations of DMOG showed a dose-dependent decrease of phenotypic and functional hematopoietic progenitor generation from CPs, with a slight reduction in total cell numbers seen at the highest DMOG concentration (Figures 7F, 7G, S7C, and S7D). Strikingly, the effect of DMOG was reversed in YS cultures, in which we observed an increase in cell numbers, CFU-C and VE-Cad+ CD45+ cells, though c-Kit+ Sca1+ hematopoietic progenitors were also reduced in YS (Figures 7F, 7G, and S7D). Overall, these results suggest that the modulation of metabolic pathways in intraembryonic, but not YS, cells undergoing EHT, likely driven by the onset of circulation, is required to achieve a normal hematopoietic output.

DISCUSSION

The intraembryonic development of the HSC lineage coincides with the establishment of blood circulation. While several studies reported a role for blood flow in embryonic hematopoietic development, its effect on the emerging mammalian HSC lineage was not directly assessed (Adamo et al., 2009; Diaz et al., 2015; Jing et al., 2015; Kim et al., 2015; Lundin et al., 2020; North et al., 2009; Wang et al., 2011). Here, using *in vivo* and *ex vivo* functional assays and single cell transcriptomics, we directly examined the earliest stages of HSC development, the HE and pro-HSC, in *Ncx1*$^{-/-}$ mouse embryos which lack a heartbeat and functional circulation. Despite normal initiation of Runx1 expression in *Ncx1*$^{-/-}$ HE, pro-HSC showed a decrease in *Runx1* and its downstream hematopoietic program, borne out in the inability of the pro-HSCs to develop into pre-HSC *in vivo* or fully functional HSCs *ex vivo*. Consistently, single-cell transcriptomics analyses showed delayed progression of *Ncx1*$^{-/-}$ HE and pro-HSCs along the EHT trajectory, with few cells progressing to *Cd44*+, *Foxc2*+, *Sox17*+, *Runx1*^{lo} HE, and *Runx1*^{hi} *Adgrg1*+ pro-HSC stages. Therefore, our work identifies the HE to pro-HSCs transition as the stage sensitive to circulation-induced effects along the path to HSC emergence.

In contrast to HSCs, the generation of YS primitive erythroblasts (Lux et al., 2008), EMPs (Frame et al., 2016), B1 B and T cell-restricted progenitors (Yoshimoto et al., 2011; Yoshimoto et al., 2012), and placental multi-lineage progenitors (Rhodes et al., 2008) was shown to be independent of circulation. The presence of lymphoid potential in the absence of blood flow has at times been interpreted to indicate that HSCs can emerge *de novo* in these tissues and may, similar to EMPs (Lux et al., 2008), colonize the embryo by circulation. The lack of functional HSCs in our *ex vivo* cultures of *Ncx1*$^{-/-}$ YS argues against a YS origin of HSCs, in line with previous reports (Rybtsov et al., 2014) (Cumano et al., 2001; Ganuza et al., 2018). Interestingly, a recent study reported two separate populations of CD45+ HSPCs in E10.5 aortic cell clusters, reflecting an initial wave of lympho-myeloid-biased progenitors, followed by precursors of hematopoietic stem cells (pre-HSCs), reinforcing that lymphoid potential is not indicative of the presence of definitive HSCs (Zhu et al., 2020).

We observed a strong downregulation of *Jag1* mRNA in *Ncx1*$^{-/-}$ HE and pro-HSC, while Dll4 expression was unaffected. This changed Jag1/Dll4 ratio may be due to a lack of laminar shear stress, as described for endothelial cells *in vitro* (Driessen et al., 2018). In HSC development Jag1 acts antagonistic to Dll4 and elicits a low-strength Notch signal that induces the hematopoietic program; in the absence of Jag1, endothelial cells are subject to a higher strength Dll4 signal which specifies the arterial program and acts as a negative regulator of HSC formation (Gama-Norton et al., 2015; Porcheri et al., 2020; Robert-Moreno et al., 2008). Nevertheless, the inability of exogenous Jag1 to rescue *Ncx1*$^{-/-}$ co-aggregate cultures suggests that, in addition to Notch, circulation acts through other pathways to induce HSC formation. This is in agreement with various pathways we and others found affected in *Ncx1*$^{-/-}$ EHT and the multitude of mechanosensory pathways known for endothelial cells (Baratchi et al., 2017; Chatterjee, 2018). Moreover, the pro-hematopoietic action of circulation is likely a result of both cell-intrinsic and extrinsic effects, as indicated by the alterations in the

the same experimental groups were pooled for analysis. Color code indicates LogFC (fold change) of dofetilide treated versus control (untreated) or 1% O_2 versus 20% O_2.

(D) Expression of endothelial and hematopoietic genes in Ter119- VE-Cad+ 23GFP+ cells sorted from control and dofetilide-treated E9.5 (21-27sp) embryos, analyzed by multiplexed mini-bulk qRT-PCR on replicates of 25 cells. Data (mean ± SD) from the same 2 independent experiments as in (C).

(E) Schematic of explant cultures of wild-type E9.5 (19-26sp) CPs and YSs cultured in presence of DMOG. Explants were analyzed individually.

(F) Flow cytometry analysis of DMOG CP explant cultures. N = 11 (DMSO); N = 9 (DMOG 0.1 mM); N = 7 (DMOG 0.5 mM); N = 7 (DMOG 1mM). Data from 4 independent experiments. Data are mean ± SD.

(G) Analysis of CFU-C in CP DMOG explant cultures. N = 14 (DMSO); N = 12 (DMOG 0.1 mM); N = 7 (DMOG 0.5 mM); N = 10 (DMOG 1mM), 5 independent experiments. Data are mean ± SD.

(H) Flow cytometry analysis of YS DMOG explant cultures. YS: N = 8 (DMSO); N = 7 (DMOG 0.1 mM); N = 7 (DMOG 0.5 mM); N = 4 (DMOG 1mM). Data from 4 independent experiments. Data are mean ± SD.

(I) Analysis of CFU-C in YS DMOG explant cultures. Replicates are the same as (H). Data are mean ± SD.

microenvironment shown here. Interestingly, Jag1 may play a role in this too, as conditional deletion of Jag1 in Tie2-expressing cells resulted in a loss of peri-aortic SMCs (High et al., 2008), a phenotype similar to that seen in *Ncx1*$^{-/-}$ embryos. Other niche factors that may contribute to the *Ncx1*$^{-/-}$ phenotype are endothelial Kit ligand, involved in pre-HSC maturation *in vivo* (Azzoni et al., 2018), and BMP and Hedgehog, known to affect this process *ex vivo* (Souilhol et al., 2016a). In addition, our single cell transcriptomics analysis suggests an important role for hypoxia-induced changes in HSC development.

Under 'physiologic hypoxia' of the early embryo, the hypoxia response pathway was shown to control HSPC emergence (Adelman et al., 1999; Gerri et al., 2018; Harris et al., 2013; Imanirad et al., 2014; Lim et al., 2017) alongside promoting endothelial development that results in increased oxygen levels in the embryo (Michiels et al., 2000; Wong et al., 2017). In the absence of circulation these processes are perturbed, and oxygen levels cannot rise. Indeed, transcriptomics analysis identified a clear hypoxic signature in *Ncx1*$^{-/-}$ cells, including a strong upregulation of the hypoxia-induced gene *Adm*, encoding a vasodilatory peptide likely involved in the vasodilation seen in the absence of blood flow. Adm also increases cAMP production, which plays a role in HSC emergence (Diaz et al., 2015; Jing et al., 2015; Kim et al., 2015). In addition, Adm itself and its receptor Ramp2 were recently implicated in HSPC emergence. Adm inhibition decreased CFU-C in the E9.5 mouse aorta, but much less at E10.5, implying an early role for this pathway (Yvernogeau et al., 2020). Such an early role is supported by the sharp decrease in *Adm* expression we observed at the E9.5 HE stage of the wild-type pseudotime trajectory. Interestingly, *Adm* was shown to induce *Dll4* expression but not other Notch ligands (Lanner et al., 2013). Our results suggest that the Adm-Ramp2 pathway plays an important role in sensing circulation-related changes in embryonic endothelium and HSC precursors and its downregulation may be key for EHT to proceed normally.

In the adult, HSC metabolism is subject to tight regulation and is linked to HSC function (Chandel et al., 2016; Ito et al., 2019). Our single-cell transcriptomics suggests that under normal conditions cells undergoing EHT switch from glycolysis to OxPhos as their main source of energy. This is likely to be part of the overall decrease in glycolysis and increase in OxPhos that occurs in the mouse embryo between E8.5 to E10.5 (Bulusu et al., 2017; Oginuma et al., 2017), concomitant with the onset of circulation. A recent study also reported a decrease of glycolytic activity in mouse HE (Oatley et al., 2020) and glucose metabolism was shown to impact HSC development in zebrafish (Harris et al., 2013). In the absence of circulation, the glycolysis to OxPhos metabolic switch does not occur. To assess whether such a metabolic switch plays a causal role in hematopoietic development, separately from other effects of circulation, we experimentally induced a hypoxic response in cells undergoing EHT. Interestingly, we observed that induction of glycolysis at the expense of OxPhos was associated with a decrease in hematopoietic progeny in cultures of intraembryonic, but not YS-derived, cells. Thus, it will be of interest to further explore the metabolic control of EHT as these pathways could be promising targets to improve the generation of specific populations of HSPC from pluripotent cells *in vitro*.

In summary, our work provides insight into the events associated with the onset of circulation that promote embryonic HSC development. We identified the relevant cellular targets, showed that circulation shapes the hematopoietic microenvironment, and suggest a functional role for the response to hypoxia and metabolic pathways. Our transcriptomics data highlight the complexity of the *in vivo* response to the onset of circulation. Further work aimed at characterizing individual pathways and their crosstalk, with the goal of obtaining a comprehensive understanding of embryonic HSC development, will guide the future development of effective strategies to improve *in vitro* production of HSPC for regenerative medicine.

Limitations of the study

While the *Ncx1*$^{-/-}$ mouse model enables analysis of the broad effects of a lack of circulation on EHT, it does not discriminate between the roles of specific biomechanical forces associated with blood flow (i.e., wall shear stress, cyclic stretch, pulsation) or the role of nutrients disseminated via the blood stream. Different experimental models are needed to explore the functions of each of these factors. Another layer of complexity in the interpretation of the phenotype of *Ncx1*$^{-/-}$ embryos is linked to the defects in the hematopoietic microenvironment that may add to the severity of the phenotype. Single cell transcriptomics comparing wild-type and *Ncx1*$^{-/-}$ microenvironment—as well as the HE/pro-HSCs—identified, in addition to the hypoxia/metabolic signature reported here, other molecular pathways potentially involved in HSC generation. Future experiments are required to establish the individual roles of each of these in the EHT process.

STAR★METHODS

Detailed methods are provided in the online version of this paper and include the following:

- KEY RESOURCES TABLE
- RESOURCE AVAILABILITY
  - Lead contact
  - Materials availability
  - Data and code availability
- EXPERIMENTAL MODEL AND SUBJECT DETAILS
  - Mice and embryos
- METHOD DETAILS
  - Immunofluorescence analysis and imaging
  - Flow cytometry and cell sorting
  - Co-aggregation cultures
  - Explant cultures
  - Treatment of pregnant females with dofetilide
  - Multiplex Quantitative Real-Time PCR (qRT-PCR)
  - CFU-C (Colony-forming unit-culture) assays
  - Repopulation assays of co-aggregate cultures
  - Single-cell RNA sequencing of E9.5 CP
  - Generation of single-cell cDNA libraries using modified Smart-Seq2 protocol
  - Illumina library preparation and sequencing
  - scRNA-Seq analysis
- QUANTIFICATION AND STATISTICAL ANALYSIS

CellPress
OPEN ACCESS

SUPPLEMENTAL INFORMATION

Supplemental information can be found online at https://doi.org/10.1016/j.celrep.2021.110103.

ACKNOWLEDGMENTS

The authors would like to thank Kevin Clark and Paul Sopp at the WIMM Flow Cytometry Facility for help with cell sorting and flow analysis; Christoffer Lagerholm at the Wolfson Imaging Centre Oxford for technical expertise and assistance with imaging; Biomedical Services staff for animal care. We also like to thank past and present members of the de Bruijn lab, in particular Dominic Owens and Yurim Park for helpful discussion and practical advice. This work was supported in Oxford by programmes in the MRC Molecular Hematology Unit Core award [MC_UU_12009/2 to M.d.B. and MC_UU_12009/5 to S.E.W.J.], by an MRC Computational Biology Fellowship [MC_UU_12025, MRC Strategic Alliance Funding; MRC Weatherall Institute of Molecular Medicine to E.M.] and a studentship from the Oxford-MRC Doctoral Training Partnership [to A.S.D.]. The WIMM Flow Cytometry facility is supported by the MRC HIU, MRC MHU [MC_UU_12009], NIHR Oxford BRC and John Fell Fund [131/030 and 101/517], EPA fund [CF182 and CF170], WIMM Strategic Alliance awards [G0902418 and MC_UU_12025]. The Wolfson Imaging Centre Oxford is supported by the Medical Research Council via the WIMM Strategic Alliance (G0902418), the Molecular Haematology Unit (MC_UU_12009), the Human Immunology Unit (MC_UU_12010), the Wolfson Foundation (Grant 18272), and by an MRC/BBSRC/EPSRC grant (MR/K015777X/1) to M.I.C.A.—NanoscopyOxford (NanO): Novel Super-resolution Imaging Applied to Biomedical Sciences, Micron (107457/Z/15Z). The facility was supported by WIMM Strategic Alliance awards G0902418 and MC_UU_12025. EA is currently supported by a Fondazione Cariplo grant (2018-0102). The Enver lab is supported by a specialist programme grant from Blood Cancer UK and from Children with Cancer UK. Single cell analysis at the Cancer Institute is supported by a Clinical Infrastructure Grant from the MRC to TE. The Jacobsen lab is supported by grants from the Swedish Research Council, StratRegen KI and the Knut and Alice Wallenberg Foundation. D.B.S. is supported by a BHF Senior Basic Science Research Fellowship (FS/17/55/33100).

AUTHOR CONTRIBUTIONS

E.A. designed and led the study, performed experiments, analyzed and interpreted data, wrote the manuscript; V.F., C.R., E.D., J.B. performed experiments, analyzed and interpreted data; G.A. performed experiments, analyzed and interpreted data and performed bioinformatic analysis; C.B., M.N., A.P. performed experiments; C.J., A.S.D. and J.H. performed bioinformatic analysis; S.J.C. generated the Ncx1 null transgenic mouse line; E.M. provided computational expertise and interpreted data; S.E.W.J., D.B.S., A.L.H. and T.E. provided reagents, interpreted data and edited the manuscript; M..F.T.R.d.B. designed, supervised and led the study, analyzed and interpreted data and wrote the manuscript.

DECLARATION OF INTERESTS

The authors declare no competing interests.

Received: June 24, 2020
Revised: October 11, 2021
Accepted: November 15, 2021
Published: December 14, 2021

REFERENCES

Adamo, L., Naveiras, O., Wenzel, P.L., McKinney-Freeman, S., Mack, P.J., Gracia-Sancho, J., Suchy-Dicey, A., Yoshimoto, M., Lensch, M.W., Yoder, M.C., et al. (2009). Biomechanical forces promote embryonic haematopoiesis. Nature *459*, 1131–1135.

Adelman, D.M., Maltepe, E., and Simon, M.C. (1999). Multilineage embryonic hematopoiesis requires hypoxic ARNT activity. Genes Dev. *13*, 2478–2483.

Azzoni, E., Frontera, V., McGrath, K.E., Harman, J., Carrelha, J., Nerlov, C., Palis, J., Jacobsen, S.E.W., and de Bruijn, M.F. (2018). Kit ligand has a critical role in mouse yolk sac and aorta-gonad-mesonephros hematopoiesis. EMBO Rep. *19*, e45477.

Baratchi, S., Khoshmanesh, K., Woodman, O.L., Potocnik, S., Peter, K., and McIntyre, P. (2017). Molecular sensors of blood flow in endothelial cells. Trends in Molecular Medicine *23*, 850–868, 868.

Batsivari, A., Rybtsov, S., Souilhol, C., Binagui-Casas, A., Hills, D., Zhao, S., Travers, P., and Medvinsky, A. (2017). Understanding hematopoietic stem cell development through functional correlation of their proliferative status with the intra-aortic cluster architecture. Stem Cell Reports *8*, 1549–1562.

Becht, E., McInnes, L., Healy, J., Dutertre, C.A., Kwok, I.W.H., Ng, L.G., Ginhoux, F., and Newell, E.W. (2018). Dimensionality reduction for visualizing single-cell data using UMAP. Nat. Biotechnol. *37*, 38–44.

Bee, T., Swiers, G., Muroi, S., Pozner, A., Nottingham, W., Santos, A.C., Li, P.S., Taniuchi, I., and de Bruijn, M.F. (2010). Nonredundant roles for Runx1 alternative promoters reflect their activity at discrete stages of developmental hematopoiesis. Blood *115*, 3042–3050.

Bertrand, J.Y., Chi, N.C., Santoso, B., Teng, S., Stainier, D.Y., and Traver, D. (2010). Haematopoietic stem cells derive directly from aortic endothelium during development. Nature *464*, 108–111.

Blondel, V.D., Guillaume, J.-L., Lambiotte, R., and Lefebvre, E. (2008). Fast unfolding of communities in large networks. J. Stat. Mech, Published online October 9, 2008. https://doi.org/10.1088/1742-5468/2008/10/P10008.

Boisset, J.C., van Cappellen, W., Andrieu-Soler, C., Galjart, N., Dzierzak, E., and Robin, C. (2010). In vivo imaging of haematopoietic cells emerging from the mouse aortic endothelium. Nature *464*, 116–120.

Bulusu, V., Prior, N., Snaebjornsson, M.T., Kuehne, A., Sonnen, K.F., Kress, J., Stein, F., Schultz, C., Sauer, U., and Aulehla, A. (2017). Spatiotemporal analysis of a gycolytic activity gradient linked to mouse embryo mesoderm development. Dev. Cell *40*, 331–341.e4.

Butler, A., Hoffman, P., Smibert, P., Papalexi, E., and Satija, R. (2018). Integrating single-cell transcriptomic data across different conditions, technologies, and species. Nat. Biotechnol. *36*, 411–420.

Chandel, N.S., Jasper, H., Ho, T.T., and Passegué, E. (2016). Metabolic regulation of stem cell function in tissue homeostasis and organismal ageing. Nat. Cell Biol. *18*, 823–832.

Chatterjee, S. (2018). Endothelial mechanotransduction, redox signaling and the regulation of vascular inflammatory pathways. Front. Physiol. *9*, 524.

Chen, J., Bardes, E.E., Aronow, B.J., and Jegga, A.G. (2009). ToppGene Suite for gene list enrichment analysis and candidate gene prioritization. Nucleic Acids Res. *37*, W305-11.

Chong, D.C., Koo, Y., Xu, K., Fu, S., and Cleaver, O. (2011). Stepwise arteriovenous fate acquisition during mammalian vasculogenesis. Developmental Dynamics. *240*, 2153–2165.

Coifman, R.R., Lafon, S., Lee, A.B., Maggioni, M., Nadler, B., Warner, F., and Zucker, S.W. (2005). Geometric diffusions as a tool for harmonic analysis and structure definition of data: diffusion maps. Proc. Natl. Acad. Sci. USA *102*, 7426–7431.

Crisan, M., Solaimani Kartalaei, P., Neagu, A., Karkanpouna, S., Yamada-Inagawa, T., Purini, C., Vink, C.S., van der Linden, R., van Ijcken, W., Chuva de Sousa Lopes, S.M., et al. (2016). BMP and hedgehog regulate distinct AGM hematopoietic stem cells *ex vivo*. Stem Cell Reports *6*, 383–395.

Cumano, A., Ferraz, J.C., Klaine, M., Di Santo, J.P., and Godin, I. (2001). Intraembryonic, but not yolk sac hematopoietic precursors, isolated before circulation, provide long-term multilineage reconstitution. Immunity *15*, 477–485.

de Bruijn, M., and Dzierzak, E. (2017). Runx transcription factors in the development and function of the definitive hematopoietic system. Blood *129*, 2061–2069.

de Bruijn, M.F., Speck, N.A., Peeters, M.C., and Dzierzak, E. (2000). Definitive hematopoietic stem cells first develop within the major arterial regions of the mouse embryo. EMBO J. *19*, 2465–2474.

Diaz, M.F., Li, N., Lee, H.J., Adamo, L., Evans, S.M., Willey, H.E., Arora, N., Torisawa, Y.-s., Vickers, D.A., Morris, S.A., et al. (2015). Biomechanical forces promote blood development through prostaglandin E2 and the cAMP–PKA signaling axis. The Journal of Experimental Medicine. *212*, 665–680.

Dobin, A., Davis, C.A., Schlesinger, F., Drenkow, J., Zaleski, C., Jha, S., Batut, P., Chaisson, M., and Gingeras, T.R. (2013). STAR: Ultrafast universal RNA-seq aligner. Bioinformatics *29*, 15–21.

Driessen, R.C.H., Stassen, O.M.J.A., Sjöqvist, M., Suarez Rodriguez, F., Grolleman, J., Bouten, C.V.C., and Sahlgren, C.M. (2018). Shear stress induces expression, intracellular reorganization and enhanced Notch activation potential of Jagged1. Integr. Biol. *10*, 719–726.

Fang, J.S., Coon, B.G., Gillis, N., Chen, Z., Qiu, J., Chittenden, T.W., Burt, J.M., Schwartz, M.A., and Hirschi, K.K. (2017). Shear-induced Notch-Cx37-p27 axis arrests endothelial cell cycle to enable arterial specification. Nat. Commun. *8*, 2149.

Fraisl, P., Aragonés, J., and Carmeliet, P. (2009). Inhibition of oxygen sensors as a therapeutic strategy for ischaemic and inflammatory disease. Nat. Rev. Drug Discov. *8*, 139–152.

Frame, J.M., Fegan, K.H., Conway, S.J., McGrath, K.E., and Palis, J. (2016). Definitive hematopoiesis in the yolk sac emerges from Wnt-responsive hemogenic endothelium independently of circulation and arterial identity. Stem Cells. *34*, 431–444.

Gama-Norton, L., Ferrando, E., Ruiz-Herguido, C., Liu, Z., Guiu, J., Islam, A.B.M.M.K., Lee, S.-U., Yan, M., Guidos, C.J., López-Bigas, N., et al. (2015). Notch signal strength controls cell fate in the haemogenic endothelium. Nat. Commun. *6*, 8510.

Ganuza, M., Chabot, A., Tang, X., Bi, W., Natarajan, S., Carter, R., Gawad, C., Kang, G., Cheng, Y., and McKinney-Freeman, S. (2018). Murine hematopoietic stem cell activity is derived from pre-circulation embryos but not yolk sacs. Nat. Commun. *9*, 5405.

Gerri, C., Marass, M., Rossi, A., and Stainier, D.Y.R. (2018). Hif-1α and Hif-2α regulate hemogenic endothelium and hematopoietic stem cell formation in zebrafish. Blood *131*, 963–973.

Hafemeister, C., and Satija, R. (2019). Normalization and variance stabilization of single-cell RNA-seq data using regularized negative binomial regression. Genome Biol. *20*, 296.

Haghverdi, L., Büttner, M., Wolf, F.A., Buettner, F., and Theis, F.J. (2016). Diffusion pseudotime robustly reconstructs lineage branching. Nat. Methods *13*, 845–848.

Hao, Y., Hao, S., Andersen-Nissen, E., Mauck, W.M., 3rd, Zheng, S., Butler, A., Lee, M.J., Wilk, A.J., Darby, C., Zager, M., et al. (2021). Integrated analysis of multimodal single-cell data. Cell *184*, 3573–3587.e29.

Harris, J.M., Esain, V., Frechette, G.M., Harris, L.J., Cox, A.G., Cortes, M., Garnaas, M.K., Carroll, K.J., Cutting, C.C., Khan, T., et al. (2013). Glucose metabolism impacts the spatiotemporal onset and magnitude of HSC induction in vivo. Blood *121*, 2483–2493.

Hartley, S.W., and Mullikin, J.C. (2015). QoRTs: a comprehensive toolset for quality control and data processing of RNA-Seq experiments. BMC Bioinformatics *16*, 224.

High, F.A., Lu, M.M., Pear, W.S., Loomes, K.M., Kaestner, K.H., and Epstein, J.A. (2008). Endothelial expression of the Notch ligand Jagged1 is required for vascular smooth muscle development. Proceedings of the National Academy of Sciences. *105*, 1955–1959.

Huang, C., Jackson, M., Samuel, K., Taylor, A.H., Lowell, S., and Forrester, L.M. (2013). Haematopoietic differentiation is inhibited when Notch activity is enhanced in FLK1(+) mesoderm progenitors. Stem Cell Res. (Amst.) *11*, 1273–1287.

Hwa, J.J., Beckouche, N., Huang, L., Kram, Y., Lindskog, H., and Wang, R.A. (2017). Abnormal arterial-venous fusions and fate specification in mouse embryos lacking blood flow. Sci. Rep. *7*, 11965.

Imanirad, P., Solaimani Kartalaei, P., Crisan, M., Vink, C., Yamada-Inagawa, T., de Pater, E., Kurek, D., Kaimakis, P., van der Linden, R., Speck, N., and Dzierzak, E. (2014). HIF1α is a regulator of hematopoietic progenitor and stem cell development in hypoxic sites of the mouse embryo. Stem Cell Res. (Amst.) *12*, 24–35.

Ito, K., Bonora, M., and Ito, K. (2019). Metabolism as master of hematopoietic stem cell fate. Int. J. Hematol. *109*, 18–27.

Ivanovs, A., Rybtsov, S., Welch, L., Anderson, R.A., Turner, M.L., and Medvinsky, A. (2011). Highly potent human hematopoietic stem cells first emerge in the intraembryonic aorta-gonad-mesonephros region. J. Exp. Med. *208*, 2417–2427.

Jaakkola, P., Mole, D.R., Tian, Y.M., Wilson, M.I., Gielbert, J., Gaskell, S.J., von Kriegsheim, A., Hebestreit, H.F., Mukherji, M., Schofield, C.J., et al. (2001). Targeting of HIF-alpha to the von Hippel-Lindau ubiquitylation complex by O2-regulated prolyl hydroxylation. Science *292*, 468–472.

Jang, I.H., Lu, Y.-F., Zhao, L., Wenzel, P.L., Kume, T., Datta, S.M., Arora, N., Guiu, J., Lagha, M., Kim, P.G., et al. (2015). Notch1 acts via Foxc2 to promote definitive hematopoiesis via effects on hemogenic endothelium. Blood. *125*, 1418–1426.

Ji, R.P., Phoon, C.K., Aristizábal, O., McGrath, K.E., Palis, J., and Turnbull, D.H. (2003). Onset of cardiac function during early mouse embryogenesis coincides with entry of primitive erythroblasts into the embryo proper. Circ. Res. *92*, 133–135.

Jing, L., Tamplin, O.J., Chen, M.J., Deng, Q., Patterson, S., Kim, P.G., Durand, E.M., McNeil, A., Green, J.M., Matsuura, S., et al. (2015). Adenosine signaling promotes hematopoietic stem and progenitor cell emergence. The Journal of Experimental Medicine. *212*, 649–663.

Jones, E.A., Yuan, L., Breant, C., Watts, R.J., and Eichmann, A. (2008). Separating genetic and hemodynamic defects in neuropilin 1 knockout embryos. Development *135*, 2479–2488.

Kanehisa, M., Furumichi, M., Tanabe, M., Sato, Y., and Morishima, K. (2017). KEGG: New perspectives on genomes, pathways, diseases and drugs. Nucleic Acids Res. *45* (*D1*), D353–D361.

Kim, J.W., Tchernyshyov, I., Semenza, G.L., and Dang, C.V. (2006). HIF-1-mediated expression of pyruvate dehydrogenase kinase: a metabolic switch required for cellular adaptation to hypoxia. Cell Metab. *3*, 177–185.

Kim, P.G., Nakano, H., Das, P.P., Chen, M.J., Rowe, R.G., Chou, S.S., Ross, S.J., Sakamoto, K.M., Zon, L.I., Schlaeger, T.M., et al. (2015). Flow-induced protein kinase A–CREB pathway acts via BMP signaling to promote HSC emergence. The Journal of Experimental Medicine. *212*, 633–648.

Kissa, K., and Herbomel, P. (2010). Blood stem cells emerge from aortic endothelium by a novel type of cell transition. Nature *464*, 112–115.

Koushik, S.V., Wang, J., Rogers, R., Moskoidis, D., Lambert, N.A., Creazzo, T.L., and Conway, S.J. (2001). Targeted inactivation of the sodium-calcium exchanger (Ncx1) results in the lack of a heartbeat and abnormal myofibrillar organization. The FASEB Journal *15*, 1209–1211.

Kumano, K., Chiba, S., Kunisato, A., Sata, M., Saito, T., Nakagami-Yamaguchi, E., Yamaguchi, T., Masuda, S., Shimizu, K., Takahashi, T., et al. (2003). Notch1 but not Notch2 is essential for generating hematopoietic stem cells from endothelial cells. Immunity *18*, 699–711.

Lanner, F., Lee, K.L., Ortega, G.C., Sohl, M., Li, X., Jin, S., Hansson, E.M., Claesson-Welsh, L., Poellinger, L., Lendahl, U., and Farnebo, F. (2013). Hypoxia-induced arterial differentiation requires adrenomedullin and Notch signaling. Stem Cells and Development *22*, 1360–1369.

Li, X., Sun, X., and Carmeliet, P. (2019). Hallmarks of endothelial cell metabolism in health and disease. Cell Metab. *30*, 414–433.

Lim, S.E., Esain, V., Kwan, W., Theodore, L.N., Cortes, M., Frost, I.M., Liu, S.Y., and North, T.E. (2017). HIF1α-induced PDGFRβ signaling promotes developmental HSC production via IL-6 activation. Exp. Hematol. *46*, 83–95.e6.

Livak, K.J., Wills, Q.F., Tipping, A.J., Datta, K., Mittal, R., Goldson, A.J., Sexton, D.W., and Holmes, C.C. (2013). Methods for qPCR gene expression profiling applied to 1440 lymphoblastoid single cells. Methods *59*, 71–79.

Lucitti, J.L., Jones, E.A.V., Huang, C., Chen, J., Fraser, S.E., and Dickinson, M.E. (2007). Vascular remodeling of the mouse yolk sac requires hemodynamic force. Development. *134*, 3317–3326.

Lundin, V., Sugden, W.W., Theodore, L.N., Sousa, P.M., Han, A., Chou, S., Wrighton, P.J., Cox, A.G., Ingber, D.E., Goessling, W., et al. (2020). YAP regulates hematopoietic stem cell formation in response to the biomechanical forces of blood flow. Dev. Cell *52*, 446–460.e5.

Lux, C.T., Yoshimoto, M., McGrath, K., Conway, S.J., Palis, J., and Yoder, M.C. (2008). All primitive and definitive hematopoietic progenitor cells emerging before E10 in the mouse embryo are products of the yolk sac. Blood. *111*, 3435–3438.

Mack, J.J., Mosqueiro, T.S., Archer, B.J., Jones, W.M., Sunshine, H., Faas, G.C., Briot, A., Aragón, R.L., Su, T., Romay, M.C., et al. (2017). NOTCH1 is a mechanosensor in adult arteries. Nat. Commun. *8*, 1620.

Mariani, S.A., Li, Z., Rice, S., Krieg, C., Fragkogianni, S., Robinson, M., Vink, C.S., Pollard, J.W., and Dzierzak, E. (2019). Pro-inflammatory aorta-associated macrophages are involved in embryonic development of hematopoietic stem cells. Immunity *50*, 1439–1452.e5.

Martin, M. (2011). Cutadapt removes adapter sequences from high-throughput sequencing reads. EMBnet *17*, Published online May 2, 2011. https://doi.org/10.14806/ej.17.1.200.

McGinnis, C.S., Murrow, L.M., and Gartner, Z.J. (2019). DoubletFinder: Doublet detection in sngle-cell RNA sequencing data using artificial nearest neighbors. Cell Syst. *8*, 329–337.e4.

McGrath, K.E., Koniski, A.D., Malik, J., and Palis, J. (2003).). Circulation is established in a stepwise pattern in the mammalian embryo. Blood. *101*, 1669–1676.

Medvinsky, A., and Dzierzak, E. (1996). Definitive hematopoiesis is autonomously initiated by the AGM region. Cell *86*, 897–906.

Michiels, C., Arnould, T., and Remacle, J. (2000). Endothelial cell responses to hypoxia: initiation of a cascade of cellular interactions. Biochim. Biophys. Acta *1497*, 1–10.

Mirshekar-Syahkal, B., Haak, E., Kimber, G.M., van Leusden, K., Harvey, K., O'Rourke, J., Laborda, J., Bauer, S.R., de Bruijn, M.F., Ferguson-Smith, A.C., et al. (2013). Dlk1 is a negative regulator of emerging hematopoietic stem and progenitor cells. Haematologica *98*, 163–171.

North, T., Gu, T.L., Stacy, T., Wang, Q., Howard, L., Binder, M., Marín-Padilla, M., and Speck, N.A. (1999). Cbfa2 is required for the formation of intra-aortic hematopoietic clusters. Development *126*, 2563–2575.

North, T.E., de Bruijn, M.F., Stacy, T., Talebian, L., Lind, E., Robin, C., Binder, M., Dzierzak, E., and Speck, N.A. (2002). Runx1 expression marks long-term repopulating hematopoietic stem cells in the midgestation mouse embryo. Immunity *16*, 661–672.

North, T.E., Goessling, W., Peeters, M., Li, P., Ceol, C., Lord, A.M., Weber, G.J., Harris, J., Cutting, C.C., Huang, P., et al. (2009). Hematopoietic stem cell development is dependent on blood flow. Cell. *137*, 736–748.

Oatley, M., Bölükbası, O.V., Svensson, V., Shvartsman, M., Ganter, K., Zirngibl, K., Pavlovich, P.V., Milchevskaya, V., Foteva, V., Natarajan, K.N., et al. (2020). Single-cell transcriptomics identifies CD44 as a marker and regulator of endothelial to haematopoietic transition. Nat. Commun. *11*, 586.

Oehler, M.K., Norbury, C., Hague, S., Rees, M.C., and Bicknell, R. (2001). Adrenomedullin inhibits hypoxic cell death by upregulation of Bcl-2 in endometrial cancer cells: a possible promotion mechanism for tumour growth. Oncogene *20*, 2937–2945.

Oginuma, M., Moncuquet, P., Xiong, F., Karoly, E., Chal, J., Guevorkian, K., and Pourquié, O. (2017). A gradient of glycolytic activity coordinates FGF and Wnt signaling during elongation of the body axis in amniote embryos. Dev. Cell *40*, 342–353.e10.

Picelli, S., Faridani, O.R., Björklund, A.K., Winberg, G., Sagasser, S., and Sandberg, R. (2014). Full-length RNA-seq from single cells using Smart-seq2. Nat. Protoc. *9*, 171–181.

Pijuan-Sala, B., Griffiths, J.A., Guibentif, C., Hiscock, T.W., Jawaid, W., Calero-Nieto, F.J., Mulas, C., Ibarra-Soria, X., Tyser, R.C.V., Ho, D.L.L., et al. (2019). A single-cell molecular map of mouse gastrulation and early organogenesis. Nature *566*, 490–495.

Porcheri, C., Golan, O., Calero-Nieto, F.J., Thambyrajah, R., Ruiz-Herguido, C., Wang, X., Catto, F., Guillén, Y., Sinha, R., González, J., et al. (2020). Notch ligand Dll4 impairs cell recruitment to aortic clusters and limits blood stem cell generation. EMBO J. *39*, e104270.

Reimand, J., Kull, M., Peterson, H., Hansen, J., and Vilo, J. (2007). g:Profiler–a web-based toolset for functional profiling of gene lists from large-scale experiments. Nucleic Acids Res. *35*, W193-200.

Rhodes, K.E., Gekas, C., Wang, Y., Lux, C.T., Francis, C.S., Chan, D.N., Conway, S., Orkin, S.H., Yoder, M.C., and Mikkola, H.K.A. (2008). The emergence of hematopoietic stem cells is initiated in the placental vasculature in the absence of circulation. Cell Stem Cell. *2*, 252–263.

Richard, C., Drevon, C., Canto, P.Y., Villain, G., Bollérot, K., Lempereur, A., Teillet, M.A., Vincent, C., Rosselló Castillo, C., Torres, M., et al. (2013). Endothelio-mesenchymal interaction controls runx1 expression and modulates the notch pathway to initiate aortic hematopoiesis. Dev. Cell *24*, 600–611.

Ritchie, H., Oakes, D., Hung, T.T., Hegedus, E., Sood, S., and Webster, W. (2015). The effect of dofetilide on the heart rate of GD11 and GD13 rat embryos, in vivo, using ultrasound. Birth Defects Res. B Dev. Reprod. Toxicol. *104*, 196–203.

Robert-Moreno, A., Espinosa, L., de la Pompa, J.L., and Bigas, A. (2005). RBPjkappa-dependent Notch function regulates Gata2 and is essential for the formation of intra-embryonic hematopoietic cells. Development *132*, 1117–1126.

Robert-Moreno, A., Guiu, J., Ruiz-Herguido, C., López, M.E., Inglés-Esteve, J., Riera, L., Tipping, A., Enver, T., Dzierzak, E., Gridley, T., et al. (2008). Impaired embryonic haematopoiesis yet normal arterial development in the absence of the Notch ligand Jagged1. EMBO J. *27*, 1886–1895.

Rybtsov, S., Sobiesiak, M., Taoudi, S., Souilhol, C., Senserrich, J., Liakhovitskaia, A., Ivanovs, A., Frampton, J., Zhao, S., and Medvinsky, A. (2011). Hierarchical organization and early hematopoietic specification of the developing HSC lineage in the AGM region. J. Exp. Med. *208*, 1305–1315.

Rybtsov, S., Batsivari, A., Bilotkach, K., Paruzina, D., Senserrich, J., Nerushev, O., and Medvinsky, A. (2014). Tracing the origin of the HSC hierarchy reveals an SCF-dependent, IL-3-independent CD43(-) embryonic precursor. Stem Cell Reports. *3*, 489–501.

Satija, R., Farrell, J.A., Gennert, D., Schier, A.F., and Regev, A. (2015). Spatial reconstruction of single-cell gene expression data. Nat. Biotechnol. *33*, 495–502.

Solaimani Kartalaei, P., Yamada-Inagawa, T., Vink, C.S., de Pater, E., van der Linden, R., Marks-Bluth, J., van der Sloot, A., van den Hout, M., Yokomizo, T., van Schaick-Solernó, M.L., et al. (2015). Whole-transcriptome analysis of endothelial to hematopoietic stem cell transition reveals a requirement for Gpr56 in HSC generation. J. Exp. Med. *212*, 93–106.

Souilhol, C., Gonneau, C., Lendinez, J.G., Batsivari, A., Rybtsov, S., Wilson, H., Morgado-Palacin, L., Hills, D., Taoudi, S., Antonchuk, J., et al. (2016a). Inductive interactions mediated by interplay of asymmetric signalling underlie development of adult haematopoietic stem cells. Nat. Commun. *7*, 10784.

Souilhol, C., Lendinez, J.G., Rybtsov, S., Murphy, F., Wilson, H., Hills, D., Batsivari, A., Binagui-Casas, A., McGarvey, A.C., MacDonald, H.R., et al. (2016b). Developing HSCs become Notch independent by the end of maturation in the AGM region. Blood. *128*, 1567–1577.

Stuart, T., Butler, A., Hoffman, P., Hafemeister, C., Papalexi, E., Mauck, W.M., 3rd, Hao, Y., Stoeckius, M., Smibert, P., and Satija, R. (2019). Comprehensive integration of single-cell data. Cell. *177*, 1888–1902.e21.

Subramanian, A., Tamayo, P., Mootha, V.K., Mukherjee, S., Ebert, B.L., Gillette, M.A., Paulovich, A., Pomeroy, S.L., Golub, T.R., Lander, E.S., and Mesirov, J.P. (2005). Gene set enrichment analysis: a knowledge-based approach for interpreting genome-wide expression profiles. Proc. Natl. Acad. Sci. USA *102*, 15545–15550.

Swiers, G., Baumann, C., O'Rourke, J., Giannoulatou, E., Taylor, S., Joshi, A., Moignard, V., Pina, C., Bee, T., Kokkaliaris, K.D., et al. (2013). Early dynamic

fate changes in haemogenic endothelium characterized at the single-cell level. Nat. Commun. *4*, 2924.

Taoudi, S., Gonneau, C., Moore, K., Sheridan, J.M., Blackburn, C.C., Taylor, E., and Medvinsky, A. (2008). Extensive hematopoietic stem cell generation in the AGM region via maturation of VE-cadherin+CD45+ pre-definitive HSCs. Cell Stem Cell *3*, 99–108.

Travnickova, J., Tran Chau, V., Julien, E., Mateos-Langerak, J., Gonzalez, C., Lelièvre, E., Lutfalla, G., Tavian, M., and Kissa, K. (2015). Primitive macrophages control HSPC mobilization and definitive haematopoiesis. Nat. Commun. *6*, 6227.

Van de Walle, I., De Smet, G., Gärtner, M., et al. (2011). Jagged2 acts as a Delta-like Notch ligand during early hematopoietic cell fate decisions. Blood. https://doi.org/10.1182/blood-2010-06-290049.

Vargel, Ö., Zhang, Y., Kosim, K., Ganter, K., Foehr, S., Mardenborough, Y., Shvartsman, M., Enright, A.J., Krijgsveld, J., and Lancrin, C. (2016). Activation of the TGFβ pathway impairs endothelial to haematopoietic transition. Sci. Rep. *6*, 21518.

Wakimoto, K., Kobayashi, K., Kuro-o, M., Yao, A., Iwamoto, T., Yanaka, N., Kita, S., Nishida, A., Azuma, S., Toyoda, Y., et al. (2000). Targeted disruption of Na+/Ca2+ exchanger gene leads to cardiomyocyte apoptosis and defects in heartbeat. Journal of Biological Chemistry. *275*, 36991–36998.

Wakimoto, K., Kuro-o, M., Yanaka, N., Komuro, I., Nabeshima, Y.-i., and Imai, Y. (2001). Expression of Na+/Ca2+ exchanger (NCX1) gene in the developmental mouse embryo and adult mouse brain. Comparative Biochemistry and Physiology Part B: Biochemistry and Molecular Biology. *130*, 191–198.

Wang, L., Zhang, P., Wei, Y., Gao, Y., Patient, R., and Liu, F. (2011). A blood flow–dependent klf2a-NO signaling cascade is required for stabilization of hematopoietic stem cell programming in zebrafish embryos. Blood. *118*, 4102–4110.

Wingett, S.W., and Andrews, S. (2018). FastQ Screen: A tool for multi-genome mapping and quality control. F1000Res. *7*, 1338.

Wolf, F.A., Angerer, P., and Theis, F.J. (2018). SCANPY: Large-scale single-cell gene expression data analysis. Genome Biol. *19*, 15.

Wong, B.W., Marsch, E., Treps, L., Baes, M., and Carmeliet, P. (2017). Endothelial cell metabolism in health and disease: impact of hypoxia. EMBO J. *36*, 2187–2203.

Xu, K., Sacharidou, A., Fu, S., Chong, D.C., Skaug, B., Chen, Z.J., Davis, G.E., and Cleaver, O. (2011). Blood vessel tubulogenesis requires Rasip1 regulation of GTPase signaling. Dev. Cell *20*, 526–539.

Yokomizo, T., Yamada-Inagawa, T., Yzaguirre, A.D., Chen, M.J., Speck, N.A., and Dzierzak, E. (2012). Whole-mount three-dimensional imaging of internally localized immunostained cells within mouse embryos. Nat. Protoc. *7*, 421–431.

Yoshimoto, M., Montecino-Rodriguez, E., Ferkowicz, M.J., Porayette, P., Shelley, W.C., Conway, S.J., Dorshkind, K., and Yoder, M.C. (2011). Embryonic day 9 yolk sac and intra-embryonic hemogenic endothelium independently generate a B-1 and marginal zone progenitor lacking B-2 potential. Proceedings of the National Academy of Sciences. *108*, 1468–1473.

Yoshimoto, M., Porayette, P., Glosson, N.L., Conway, S.J., Carlesso, N., Cardoso, A.A., Kaplan, M.H., and Yoder, M.C. (2012). Autonomous murine T-cell progenitor production in the extra-embryonic yolk sac before HSC emergence. Blood. *119*, 5706–5714.

Yvernogeau, L., Klaus, A., Maas, J., Morin-Poulard, I., Weijts, B., Schulte-Merker, S., Berezikov, E., Junker, J.P., and Robin, C. (2020). Multispecies RNA tomography reveals regulators of hematopoietic stem cell birth in the embryonic aorta. Blood *136*, 831–844.

Zhu, Q., Gao, P., Tober, J., Bennett, L., Chen, C., Uzun, Y., Li, Y., Howell, E.D., Mumau, M., Yu, W., et al. (2020). Developmental trajectory of prehematopoietic stem cell formation from endothelium. Blood *136*, 845–856.

STAR★METHODS

KEY RESOURCES TABLE

Reagent or resource	Source	Identifier
Antibodies		
Mouse anti-mouse/rat/human α-SMA (Cy3 conjugate), clone 1A4	Merck	Cat# C6198; RRID:AB_476856
Rat anti-mouse/pig c-Kit, clone 2B8	eBioscience	Cat# 14-1171-85; RRID:AB_467434
Goat anti-mouse CD31, polyclonal	R&D Systems	Cat# AF3628; RRID:AB_2161028
Rat anti-mouse CD41, clone MwReg30	BD Biosciences	Cat# 553847; RRID:AB_395084
Goat anti-mouse CD43 (M-19), polyclonal	Santa Cruz	Cat# sc-7054; RRID:AB_2194195
Goat anti-mouse DLL4, polyclonal	R&D Systems	Cat# AF1389; RRID:AB_354770
Rat anti-mouse F4/80, clone CI:A3-1	Bio-Rad	Cat# MCA497GA; RRID:AB_323806
Goat anti-mouse/rat Jagged-1, polyclonal	R&D Systems	Cat# AF599; RRID:AB_2128257
Rabbit anti-GFP	ThermoFisher	Cat# A-11122; RRID:AB_221569
Rabbit anti-human/rat/mouse NICD - Cleaved Notch1 (Val1744), clone D3B8	Cell Signaling Technology	Cat# 4147; RRID:AB_2153348
Rabbit anti-mouse/rat/human Runx1, clone EPR3099	Abcam	Cat# ab92336; RRID:AB_2049267
Rat anti-mouse VE-Cadherin, clone eBioBV13	eBioscience	Cat# 15287227; RRID:AB_842767
Donkey anti-rabbit IgG (H+L) Alexa Fluor 488	ThermoFisher	Cat# R37118; RRID:AB_2556546)
Donkey anti-rat IgG (H+L) Alexa Fluor 488	ThermoFisher	Cat# A-21208; RRID:AB_2535794
Donkey anti-goat IgG (H+L) Alexa Fluor 555	ThermoFisher	Cat# A-21432; RRID:AB_2535853
Donkey anti-rat IgG (H+L) Alexa Fluor 555	Abcam	Cat# ab150154; RRID:AB_2813834
Donkey anti-goat IgG (H+L) Alexa Fluor 647	ThermoFisher	Cat# A-21447; RRID:AB_2535864
Donkey anti-rat IgG (H+L) Alexa Fluor 647	ThermoFisher	Cat# A48272; RRID:AB_2893138
Donkey anti-rabbit IgG (H+L) Alexa Fluor 647	ThermoFisher	Cat# A-31573; RRID:AB_2536183
Rat anti-mouse/human CD45R/B220 PE-Cy5, clone RA3-6B2	BD Biosciences	Cat# 553091; RRID:AB_394621
Rat anti-mouse/human CD45R/B220 PE-Cy7, clone RA3-6B2	BD Biosciences	Cat# 552772; RRID:AB_394458
Rat anti-mouse/human CD11b BV421, clone M1/70	BD Biosciences	Cat# 562605; RRID:AB_11152949
Rat anti-mouse/human CD11b PE-Cy5, clone M1/70	BioLegend	Cat# 101210; RRID:AB_312793
Rat anti-mouse/human CD11b PE-Cy7, clone M1/70	BD Biosciences	Cat# 552850; RRID:AB_394491
Rat anti-mouse CD3e PE, clone145-2C11	BD Biosciences	Cat# 553064; RRID:AB_394597
Rat anti-mouse CD4 PE, clone RM4-5	BD Biosciences	Cat# 553048; RRID:AB_394584
Rat anti-mouse CD16/CD32 antibody (Fc Block), clone 2.4G2	BD Biosciences	Cat# 553142; RRID:AB_394657
Rat anti-mouse CD41 ef450, clone MWReg30	eBioscience	Cat# 48-0411-82; RRID:AB_1582238
Rat anti-mouse CD41 PE, clone MWReg30	BD Biosciences	Cat# 558040; RRID:AB_397004
Rat anti-mouse CD41 PE-Cy7, clone MWReg30	eBioscience	Cat# 25-0411-80; RRID:AB_1234972
Rat anti-mouse CD43 Biotin, clone eBioR2/60	eBioscience	Cat# 13-0431-82; RRID:AB_466439)
Rat anti-mouse CD43 PE, clone eBioR2/60	eBioscience	Cat# 12-0431-82; RRID:AB_465659
Rat anti-mouse CD45 APC-Cy7, Clone 30-F11	BD Biosciences	Cat# 557659; RRID:AB_396774
Rat anti-mouse CD45 ef450, Clone 30-F11	BD Biosciences	Cat# 48-0451-82; RRID:AB_1518806
Rat anti-mouse CD45 PE-CF594, Clone 30-F11	BD Biosciences	Cat# 562420; RRID:AB_11154401
Rat anti-mouse CD45 PE, Clone 30-F11	BD Biosciences	Cat# 553081; RRID:AB_394611
Mouse anti-mouse CD45.1 APC, Clone A20	BD Biosciences	Cat# 558701; RRID:AB_1645214
Mouse anti-mouse CD45.2 FITC, Clone 104	eBioscience	Cat# 11-0454-82; RRID:AB_465061
Mouse anti-mouse CD45.2 bv421, Clone 104	BD Biosciences	Cat# 562895; RRID:AB_2737873
Rat anti-mouse CD8a PE-Cy7, Clone 53-6.7	eBioscience	Cat# 25-0081-81; RRID:AB_469583
Rat anti-mouse F4/80 PE, Clone T45-2342	BD Biosciences	Cat# 565410; RRID:AB_2687527

(Continued on next page)

Continued		
Reagent or resource	Source	Identifier
Rat anti- Gr1 (Ly6G) APC-Cy7, Clone RB6-8C5	BD Biosciences	Cat# 557661; RRID:AB_396775
Rat anti-mouse c-Kit PE, Clone 2B8	BD Biosciences	Cat# 553355; RRID:AB_394806
Rat anti-mouse c-Kit PE-Cy7, Clone 2B8	BD Biosciences	Cat# 558163; RRID:AB_647250
Rat anti-mouse Ly-6A/E (Sca1) PE-Cy7, Clone D7	eBioscience	Cat# 25-5981-82; RRID:AB_469669
Rat anti-mouse Ly-6A/E (Sca1) FITC, Clone E13-161.7	BioLegend	Cat# 122505; RRID:AB_756190
Streptavidin PE	Thermo Fisher	Cat# 12-4317-87
Rat anti-mouse Ter119 APC-ef780, Clone TER-119	eBioscience	Cat# 47-5921-80; RRID:AB_1548794
Rat anti-mouse Ter119 PE-Cy7, Clone TER-119	BD Biosciences	Cat# 557853; RRID:AB_396898
Rat anti-mouse VE-Cadherin ef660, Clone eBioBV13	eBioscience	Cat# 50-1441-82; RRID:AB_11219483
Bacterial and virus strains		
Biological samples		
Chemicals, peptides, and recombinant proteins		
7-aminoactinomycin D (7-AAD)	ThermoFisher Scientific	Cat# A1310
eBioscience fixable viability dye eFluor 780	eBioscience	Cat# 65-0865-14
Hoechst 33258	Merck	Cat# 861405
Benzyl alcohol	Merck	Cat# 108006
Benzyl benzoate	Merck	Cat# B6630
Mouse SCF	PeproTech	Cat# 250-03-100ug
Mouse FLT3L	PeproTech	Cat#250-39L-100ug
Mouse IL-3	PeproTech	Cat#213-13-100ug
Hyclone FCS	Fisher Scientific	Cat# SH30070.03HI
Collagenase I	Merck	Cat# C0130
Hot StartTaq Master Mix	QIAGEN	Cat# 203445
Iscove's Modified Dulbecco's Medium (IMDM)	ThermoFisher Scientific	Cat# 12440053
Dimethyloxalylglycine (DMOG)	Merck	Cat# D3695
DAPT	Merck	Cat# 565770
Dofetilide	Merck	Cat# PZ0016
RNaseOUT Recombinant Ribonuclease Inhibitor	ThermoFisher Scientific	Cat# 10777019
SUPERase●In RNase Inhibitor (20 U/μL)	ThermoFisher Scientific	Cat # AM2694
BD Pharmlyse	BD Biosciences	RRID:AB_2869057
ERCC RNA Spike-In Mix	ThermoFisher Scientific	Cat #4456740
SMARTScribe Reverse Transcriptase	Takara	Cat #639538
SeqAmp DNA Polymerase	Takara	Cat #638509
AMPure XP	Beckman Coulter	Cat # A63881
dNTP set (100 mM)	ThermoFisher Scientific	Cat# 10297018
Magnesium chloride solution	Merck	Cat# M1028
Critical commercial assays		
Taqman assay: Atp5a1	ThermoFisher Scientific	Mm00431960_m1
Taqman assay: Dll4	ThermoFisher Scientific	Mm00444619_m1

(*Continued on next page*)

Continued

Reagent or resource	Source	Identifier
Taqman assay: Erg	ThermoFisher Scientific	Mm01214246_m1
Taqman assay: Etv2	ThermoFisher Scientific	Mm00468389_m1
Taqman assay: Gata2	ThermoFisher Scientific	Mm00492300_m1
Taqman assay: Gfi1	ThermoFisher Scientific	Mm00515855_m1
Taqman assay: Gfi1b	ThermoFisher Scientific	Mm00492318_m1
Taqman assay: Hes1	ThermoFisher Scientific	Mm01342805_m1
Taqman assay: Hey1	ThermoFisher Scientific	Mm00468865_m1
Taqman assay: Hey2	ThermoFisher Scientific	Mm00469280_m1
Taqman assay: Hprt1	ThermoFisher Scientific	Mm01545399_m1
Taqman assay: Itga2b	ThermoFisher Scientific	Mm00439768_m1
Taqman assay: Jag1	ThermoFisher Scientific	Mm00496902_m1
Taqman assay: Jag2	ThermoFisher Scientific	Mm01325629_m1
Taqman assay: Kdr	ThermoFisher Scientific	Mm01222421_m1
Taqman assay: Klf2	ThermoFisher Scientific	Mm00500486_g1
Taqman assay: Lyl1	ThermoFisher Scientific	Mm00493219_m1
Taqman assay: Lmo2	ThermoFisher Scientific	Mm01281680_m1
Taqman assay: Meis1	ThermoFisher Scientific	Mm00487664_m1
Taqman assay: Myb	ThermoFisher Scientific	Mm00501741_m1
Taqman assay: Pu.1	ThermoFisher Scientific	Mm00488142_m1
Taqman assay: Notch1	ThermoFisher Scientific	Mm00435249_m1
Taqman assay: Notch4	ThermoFisher Scientific	Mm00440525_m1
Taqman assay: Runx1	ThermoFisher Scientific	Mm01213404_m1
Taqman assay: Tal1	ThermoFisher Scientific	Mm01187033_m1
Taqman assay: Tek	ThermoFisher Scientific	Mm00443243_m1
Taqman assay: Ubc	ThermoFisher Scientific	Mm01201237_m1
Taqman assay: Pfkp	ThermoFisher Scientific	Mm00444792_m1
Taqman assay: Pkm	ThermoFisher Scientific	Mm00834102_gH
Taqman assay: Gapdh	ThermoFisher Scientific	Mm99999915_g1
Taqman assay: Pgk1	ThermoFisher Scientific	Mm00435617_m1
Taqman assay: Ldha	ThermoFisher Scientific	Mm01612132_g1
Taqman assay: Eno1	ThermoFisher Scientific	Mm01619597_g1
Taqman assay: b-Actin	ThermoFisher Scientific	Mm02619580_g1
Taqman assay: Adm	ThermoFisher Scientific	Mm01280689_g1
Taqman assay: Epor	ThermoFisher Scientific	Mm00833882_m1
Taqman assay: Slc2a1 (Glut1)	ThermoFisher Scientific	Mm00441480_m1
Taqman assay: HIF-1a	ThermoFisher Scientific	Mm00468869_m1
Taqman assay: Cav1	ThermoFisher Scientific	Mm00483057_m1
Taqman assay: Ndufa1	ThermoFisher Scientific	Mm00444593_m1
Taqman assay: Cox6c	ThermoFisher Scientific	Mm00835813_g1
Taqman assay: Cyc1	ThermoFisher Scientific	Mm00470540_m1
Taqman assay: Nrf2 (Nfe2l2)	ThermoFisher Scientific	Mm00477784_m1
Taqman assay: Cox6a1	ThermoFisher Scientific	Mm01612194_m1
Taqman assay: Cpt1a	ThermoFisher Scientific	Mm01231183_m1
Taqman assay: Pparg	ThermoFisher Scientific	Mm00440940_m1
Taqman assay: Ldlr	ThermoFisher Scientific	Mm01177349_m1
Taqman assay: Mdh1	ThermoFisher Scientific	Mm00485106_m1
Taqman assay: Taldo1	ThermoFisher Scientific	Mm00807080_g1
MethoCult GF M3434	STEMCELL Technologies	Cat# 03434

(*Continued on next page*)

Continued

Reagent or resource	Source	Identifier
Dead Cell Removal Kit	Miltenyi Biotec	Cat# 130-090-101
Chromium Next GEM Single Cell 3′ v3.1 Kit	10x Genomics	Cat# 1000269
NextSeq® 500/550 High Output Kit v2 (150 cycles)	Illumina	Cat# FC-404-2002
Nextera XT DNA Library Preparation Kit (96 samples)	Illumina	Cat# FC-131-1096
Nextera XT Index Kit v2 Set A (96 indexes, 384 samples)	Illumina	Cat # FC-131-2001
Nextera XT Index Kit v2 Set D (96 indexes, 384 samples)	Illumina	Cat # FC-131-2004
CellsDirect One-Step qRT-PCR Kit	ThermoFisher Scientific	Cat# 11753100
TaqMan Universal PCR Master Mix	ThermoFisher Scientific	Cat # 4304437
48.48 Dynamic Array IFC for Gene Expression	Fluidigm	SKU BMK-M-48.48
Control Line Fluid Kit—48.48	Fluidigm	Cat# 89000020
20X GE Sample Loading Reagent	Fluidigm	Cat #100-7610
2X Assay Loading Reagent	Fluidigm	Cat # 100-7611
**Deposited data**		
Smart-Seq2 single cell RNA sequencing data of cells undergoing EHT in wild type and Ncx1$^{-/-}$ mutant embryos	This paper	ArrayExpress:E-MTAB-8362
10x single cell RNA sequencing data of whole PAS in wild type and Ncx1$^{-/-}$ mutant embryos	This paper	ArrayExpress:E-MTAB-10945
**Experimental models: Cell lines**		
OP9	Gift from A. Medvinsky	N/A
OP9-Jag1	Gift from Ana Bigas	(Van de Walle et al., 2011)
**Experimental models: Organisms/strains**		
Mouse: Ncx1 knockout	Gift from Simon J. Conway	(Koushik et al., 2001)
Mouse: 23GFP	Our laboratory	(Bee et al., 2010; Swiers et al., 2013)
**Oligonucleotides**		
LacZL: GAC GTC TCG TTG CTG CAT AA	IDT	(Koushik et al., 2001)
LacZR: CAG CAG CAG ACC ATT TTC AA	IDT	(Koushik et al., 2001)
NCXSense: TGA TGA CCG GAG CTG GCA AC	IDT	(Koushik et al., 2001)
NCXAntisense:AGA TCA CAG TCC CTT CCG TG	IDT	(Koushik et al., 2001)
NeoInsert: CAG CGC ATC GCC TTC TAT CG	IDT	(Koushik et al., 2001)
GFP1: GAC GTG AAC GGC CAC AAG TTC A	IDT	(Bee et al., 2010)
GFP2: GTG GCG GAT CTT GAA GTT CAC C	IDT	(Bee et al., 2010)
Oligo-dt$_{30}$VN (Custom made)	IDT	N/A
**Recombinant DNA**		
**Software and algorithms**		
Imaris	Bitplane	RRID:SCR_007370
Microsoft Excel	Microsoft	RRID:SCR_016137
GraphPad Prism	GraphPad Software	RRID:SCR_002798
FlowJo	BD	RRID:SCR_008520
Photoshop	Adobe	RRID:SCR_014199

(*Continued on next page*)

Continued		
Reagent or resource	Source	Identifier
Illustrator	Adobe	RRID:SCR_010279
Zeiss Zen	Zeiss	https://www.zeiss.com/microscopy/int/products/microscope-software/zen.html
R (R-3.2.3 – R-3.6.2)	The R Foundation	https://www.r-project.org
FastQC	(Wingett and Andrews, 2018)	N/A
Cutadapt	(Martin, 2011)	N/A
STAR	(Dobin et al., 2013)	N/A
Seurat	(Butler et al., 2018; Hao et al., 2021; Satija et al., 2015)	N/A
ToppGene	(Chen et al., 2009)	https://toppgene.cchmc.org/
GSEA (v 4.0.3)	(Subramanian et al., 2005)	https://www.gsea-msigdb.org/gsea/index.jsp
Louvain	(Blondel et al., 2008)	N/A
Python	https://www.python.org/	RRID:SCR_008394
SCANPY	(Wolf et al., 2018)	N/A
G:Profiler	(Reimand et al., 2007)	N/A
SINGuLAR	Fluidigm	https://www.fluidigm.com/products-services/software/singular-analysis-toolset
QoRTs	(Hartley and Mullikin, 2015)	N/A
KEGG	(Kanehisa et al., 2017)	N/A
SCTransform	(Hafemeister and Satija, 2019)	N/A
Canonical Correlation Analysis (CCA)	(Stuart et al., 2019)	N/A
CellRanger (v5.0.0)	10x Genomics	https://support.10xgenomics.com
DoubletFinder	(McGinnis et al., 2019)	N/A
Other		
Echo 525 Acoustic Liquid Handler	Beckman Coulter	https://www.beckman.com/liquid-handlers/echo-525
Membrane Filter, Pores 0.8 μm, 25 mm Diameter	Merck	Cat# AAWP02500

RESOURCE AVAILABILITY

Lead contact

Further information and requests for resources and reagents should be directed to and will be fulfilled by the Lead Contact, Marella F.T.R. de Bruijn (marella.debruijn@imm.ox.ac.uk).

Materials availability

This study did not generate any new unique reagents.

Data and code availability

- The 10x single cell RNA sequencing (scRNA-seq) data of E9.5 wild-type and *Ncx1*$^{-/-}$ caudal parts (CPs) have been deposited in the EMBL-EBI data repository with the accession number ArrayExpress: E-MTAB-10945. The Smart-Seq2 scRNA-seq data of wild-type and *Ncx1*$^{-/-}$ cells undergoing EHT have been deposited in the EMBL-EBI data repository with the accession number ArrayExpress: E-MTAB-8362.
- This paper does not report original code.
- Any additional information required to reanalyze the data reported in this paper is available from the lead contact upon request.

EXPERIMENTAL MODEL AND SUBJECT DETAILS

Mice and embryos

All procedures involving mice were in compliance with UK Home Office regulations and the Oxford University Clinical Medicine Ethical Review Committee. Mice were housed in individually ventilated cages with free access to food and water and maintained in a 12-hour light-dark cycle. Ncx1 knockout (Koushik et al., 2001) and 23GFP mice (Bee et al., 2010; Swiers et al., 2013) were maintained on a CD45.2 C57BL/6 genetic background. Primers used for genotyping are listed in the Key Resources Table. Embryos were collected from timed pregnancies and dissected as previously described (Swiers et al., 2013). $Ncx1^{-/-}$ and wild-type embryos are siblings obtained from crosses of $Ncx1^{+/-}$ mice. Embryos were staged by counting of somite pairs.

METHOD DETAILS

Immunofluorescence analysis and imaging

Embryos were processed for whole-mount immunofluorescence analysis as previously described (Yokomizo et al., 2012). Following antibody labeling, yolk sacs (YS) were cleared in a 50% solution of glycerol in PBS at 4 degrees overnight and flat-mounted in the same solution. Image acquisition was performed at room temperature, using a Zeiss AXIO Examiner.Z1 upright microscope equipped with a Zeiss LSM-780 confocal system, with a 25x NA:0.8 DIC Imm Kor UV VIS-IR objective or a Zeiss AXIO Observer.Z1 inverted microscope equipped with a Zeiss LSM-880 confocal system using an 25x LDLCI PlnApo NA:0.8 DI or a 40x C Apo 1.1W DICIII objective. Image processing was carried out using IMARIS software (Bitplane), Zeiss Zen and Adobe Photoshop. 3D reconstructions are shown as maximum intensity projections. The Key Resources Table lists primary and secondary antibodies used for immunofluorescence analysis.

Flow cytometry and cell sorting

Single cell suspensions of embryos or YS were generated and processed for flow cytometry analysis as previously described (Azzoni et al., 2018; Swiers et al., 2013). Automatic compensation was initially set in the Diva software (BD) using CompBeads (BD) and single stained controls, and subsequently manually checked and adjusted accordingly. Gates were set using unstained, single stained and fluorescence-minus-one (FMO) controls. Dead cells were excluded based on Hoechst 33258 (Sigma) or 7-AAD (Sigma) incorporation. Data acquisition was carried out on BD LSR Fortessa, BD LSR Fortessa X-20 or BD LSR II analyzers. Cell sorting was performed on a BD FACSAria Fusion sorter using a 100 μm nozzle. Flow cytometry data was analyzed using FlowJo software (BD). The Key Resources Table lists antibodies, conjugates and viability dyes used for flow cytometry.

Co-aggregation cultures

Ex vivo co-aggregation cultures of E9.5 CP and YS were performed as described (Rybtsov et al., 2014). Briefly, CPs and YSs dissected from E9.5 (23-27sp) concepti were processed into single cells as in (Swiers et al., 2013), and co-aggregated with 100,000 OP9 cells by centrifugation in 200 μL pipette tips sealed with parafilm. Co-aggregates were cultured for 7 days on top of 0.8 μm pore size mixed cellulose membranes (Millipore) at the gas-liquid interface, in Iscove's modified Dulbecco's medium (IMDM) (GIBCO) supplemented with 20% HyClone FCS (Fisher Scientific) and in presence of SCF, FLT3L and IL-3 (100 ng/ml, PeproTech). The culture medium was changed once after 24 hours from the beginning of the experiment. For the experiments with the gamma secretase inhibitor DAPT, 50 μM DAPT or DMSO were added to the culture media at the beginning of the experiment and after 24 hours with the media change.

Explant cultures

CP and YS dissected from E9.0-E9.5 concepti were cultured for 1 to 7 days on top of 0.8 μm pore size mixed cellulose membranes (Millipore) at the gas-liquid interface, in Iscove's modified Dulbecco's medium (IMDM) (GIBCO) supplemented with 20% HyClone FCS (Fisher Scientific) and in presence of SCF, FLT3L and IL-3 (100 ng/ml, PeproTech). Explants cultured in a low oxygen environment were placed in special incubators with reduced oxygen concentration (1% O_2). For the experiments with dimethyloxalylglycine (DMOG), a range of concentrations of the inhibitor (0.1 mM, 0.5 mM, 1 mM) or DMSO were added to the culture media at the beginning of the experiment.

Treatment of pregnant females with dofetilide

Dofetilide is an anti-arrhythmia drug that is able to induce embryonic bradycardia in animal studies (Ritchie et al., 2015). In previous studies, the biggest effect of the drug on heart rate and rhythm was seen at 4h and 6h post administration (Ritchie et al., 2015). To induce a temporary reduction in embryonic circulation, pregnant 23GFP transgenic females were administered dofetilide (2mg/kg) by gavage, and embryos harvested 6 hours later.

Multiplex Quantitative Real-Time PCR (qRT-PCR)

Multiplex qRT-PCR was performed as previously described (Swiers et al., 2013). Single cells or pools of 25 cells were sorted directly into –RT/preamplification mix and analyzed using 48.48 integrated fluidic circuits on a Biomark platform (Fluidigm). For bulk qRT-

PCR, data was normalized according to expression of *Atp5a1*, *Hprt* and *Ubc* housekeeping genes and is shown as $2^{-\Delta Ct}$. For data shown in Figure 7, data was normalized according to expression of *b-Actin*, *Hprt* and *Ubc.* –RT samples were used as controls. Single cell qRT-PCR data was analyzed with SINGuLAR analysis toolkit, a dedicated R software package (Fluidigm). The default recommended value of 24 for limit of detection (LoD) was used, as determined in (Livak et al., 2013). Data is expressed as Log2(LoD – Ct) (i.e log2 of the Ct's above the limit of detection), indicated as Log_2Ex. Taqman probes used for qRT-PCR are listed in the Key Resources Table. Statistical significance was calculated using ANOVA (*$p \leq 0.05$; **$p \leq 0.01$; ***$p \leq 0.001$).

CFU-C (Colony-forming unit-culture) assays

CFU-C assays were performed using Methocult M3434 (Stem Cell Technologies). Cells were plated in duplicate dishes and cultured at 37°C, 5% CO_2 in a humidified chamber. Colonies were scored after 7 days.

Repopulation assays of co-aggregate cultures

Co-aggregates were collected after 7 days of culture and dissociated to single cells with collagenase 0.125%. Single cell suspensions containing CD45.2$^+$ CP- or YS-derived cells were injected into CD45.1$^+$ 8-week old adult 9Gy-conditioned recipients (split dose; ^{137}Cs) along with 200,000 CD45.1$^+$ spleen carrier cells. Donor-derived chimerism was determined by flow cytometry in peripheral blood (PB) at 6 and 16 weeks post transplantation. PB was treated with BD Pharmlyse (BD) prior to antibody labeling. Long-term multi-lineage reconstitution levels in PB, bone marrow, spleen and thymus were determined at 16 weeks; antibodies used in repopulation analysis are listed in the Key Resources Table. Recipients showing $\geq$ 5% donor-derived cells were considered reconstituted.

Single-cell RNA sequencing of E9.5 CP

CPs from E9 (20-23 sp) *Ncx1*$^{-/-}$ and wild-type (+/+) littermate controls (4 embryos/group) were dissected and processed into single cell suspensions as described above. Dead cells were removed by magnetic separation using Dead Cell Removal Kit (Miltenyi Biotec) as per manufacturer's instructions. Resulting cell suspension from *Ncx1*$^{-/-}$ and control embryos (93% and 95% viability, respectively) were counted and loaded onto independent channels of a Chromium chip before single cell partitioning and barcoding on Chromium Controller (10x Genomics). Single-cell cDNA synthesis and sequencing libraries were generated using a Chromium Next GEM Single Cell 3′ v3.1 Kit (10x Genomics) as per manufacturer's protocol and sequenced on Illumina NovaSeq 6000. Raw sequencing data were processed with Cell Ranger Software Suite (version 5.0.0, 10x Genomics) using the GRCm38 mouse reference genome. Further analysis was performed using Seurat package (version 4.0.4) (Hao et al., 2021) in R (version 3.6.1). Cells were filtered out based on the number of detected genes (< 500 and > 7,500), UMI (< 500 and > 100,000) and the percent of expressed mitochondrial genes (< 5%). As a result, 4,256 cells (2,616 median genes/cell) and 6,795 cells (1,682 median genes/cell) were included in downstream analysis for *Ncx1*$^{-/-}$ and wild-type samples, respectively. Data normalization (including cell cycle-associated genes and mitochondrial content regression), feature scaling and variable gene detection was perform using SCTransform (Hafemeister and Satija, 2019). DoubletFinder (McGinnis et al., 2019) was used to identify and exclude doublets from each sample independently (510 and 319 cells were removed from *Ncx1*$^{+/+}$ and *Ncx1*$^{-/-}$, respectively). The two samples were integrated using Canonical Correlation Analysis (CCA) (Stuart et al., 2019) and cluster cell identity was assigned by manual annotation based on known markers (Pijuan-Sala et al., 2019) combined with DEG analysis using the FindAllMarkers function (default settings).

Generation of single-cell cDNA libraries using modified Smart-Seq2 protocol

Smart-Seq2 single-cell cDNA libraries were prepared as previously described (Picelli et al., 2014). Briefly, single cells were FACS sorted into 96 well plates containing 4 µL lysis buffer (0.4% Triton, 1U/µl RNaseOUT inhibitor, 2.5 mM dNTPs mix, 2.5 µM oligo-dT30VN and ERCC spike-in at 5x10^7 final dilution). Plates were stored at −80°C until further use. Reverse transcription and PCR amplification steps were performed following a modified Smart-Seq2 protocol using SmartScribe RT (Takara) and SeqAmp Polymerase (Takara), respectively. 24 cycles were used for PCR amplification. cDNA libraries were purified twice with AMPure XP (Beckman) magnetic beads (1st round of purification with 0.8 to 1 beads to cDNA ratio, followed by a 2nd round using 0.6 to 1 beads to cDNA ratio) following manufacturer's instructions. Each sample was eluted in 15 µL EB buffer (QIAGEN) and stored at −20°C. Concentration and quality of the cDNA libraries were assessed using Bioanalyzer (Agilent) and Fragment Analyzer (Agilent).

Illumina library preparation and sequencing

cDNA samples were batch-processed in 384-well plates (Labcyte and Biorad) and Nextera XT DNA Preparation Kit (Illumina) was used for library preparation following a modified version of the manufacturer's protocol with 20-fold volume reduction (final volume 2.5ul). Miniaturised reactions were pipetted using the Echo 525 liquid handler (Labcyte). 50pg of cDNA per sample were used for the tagmentation followed by 13 cycles of amplification. 384 libraries were pooled using Echo 525 liquid handler and then purified with AMPure XP magnetic beads using 0.8 beads to 1 pool ratio. Pool was eluted in 25 µL EB buffer. Concentration was determined using Qubit Fluorimeter (ThermoFisher Scientific) and quality of the pooled library was assessed on Bioanalyzer (Agilent). Pooled library was diluted to a final concentration of 2pM and sequenced on the Illumina NextSeq 500 platform using NextSeq500/550 High Output Kit v2.5 (2x76 paired end cycles).

scRNA-Seq analysis

Fastq files from paired RNA sequencing reads were run through a quality control and adaptor trimming protocol using Trim Galore, a wrapper script running FastQC (Wingett and Andrews, 2018) and cutadapt (Martin, 2011); the trimmed reads where then aligned to reference indices generated from GRCm38 (“Genome Reference Consortium,” n.d.) using STAR (Dobin et al., 2013). Aligned files were run through quality control and counts for reads aligning to known genes were determined using QoRTs (Hartley and Mullikin, 2015). Post alignment analyses were run using Seurat (Butler et al., 2018; Satija et al., 2015). Gene list enrichment analyses were performed using the ToppGene suite (Chen et al., 2009). GSEA (v 4.0.3) analyses (Subramanian et al., 2005) were performed using the GseaPreranked option with genes ranked by logFC.

For diffusion pseudotime analysis, we performed PCA and computed diffusion maps (Coifman et al., 2005; Haghverdi et al., 2016) on the top 2000 highly variable genes from the wild-type dataset (identified using Seurat (Butler et al., 2018; Satija et al., 2015)). We performed clustering of the wild-type cells using the Louvain algorithm (Blondel et al., 2008) and identified the wild-type EHT trajectory based on cluster identity. We projected *Ncx1*$^{-/-}$ cells onto this dataset using Nearest Neighbors regression (with 3 neighbors) in the space of the 2000 selected genes, with wild-type diffusion pseudotime as the dependent variable. Differentially expressed genes between wild-type and *Ncx1*$^{-/-}$ over pseudotime were found by dividing the pseudotime interval into two bins and compare logarithmized normalized gene counts between genotypes using a Wilcoxon rank-sum test with FDR of 0.01 and identified KEGG pathways (Kanehisa et al., 2017). Analyses were carried out in Python, using publicly available packages *scanpy* (Wolf et al., 2018) for single-cell gene expression analysis and *g:Profiler* (Reimand et al., 2007) for functional enrichment.

QUANTIFICATION AND STATISTICAL ANALYSIS

To determine the level of significance, unpaired two-tailed Student t test assuming equal variance was used for most experiments. For single cell qRT-PCR analysis, one-way ANOVA was used. Repopulation levels in reconstituted mice were analyzed using Mann-Whitney’s U test. $p < 0.05$ was considered statistically significant (indicated by an asterisk).

Cell Reports, Volume *37*

Supplemental information

The onset of circulation triggers a metabolic switch required for endothelial to hematopoietic transition

Emanuele Azzoni, Vincent Frontera, Giorgio Anselmi, Christina Rode, Chela James, Elitza M. Deltcheva, Atanasiu S. Demian, John Brown, Cristiana Barone, Arianna Patelli, Joe R. Harman, Matthew Nicholls, Simon J. Conway, Edward Morrissey, Sten Eirik W. Jacobsen, Duncan B. Sparrow, Adrian L. Harris, Tariq Enver, and Marella F.T.R. de Bruijn

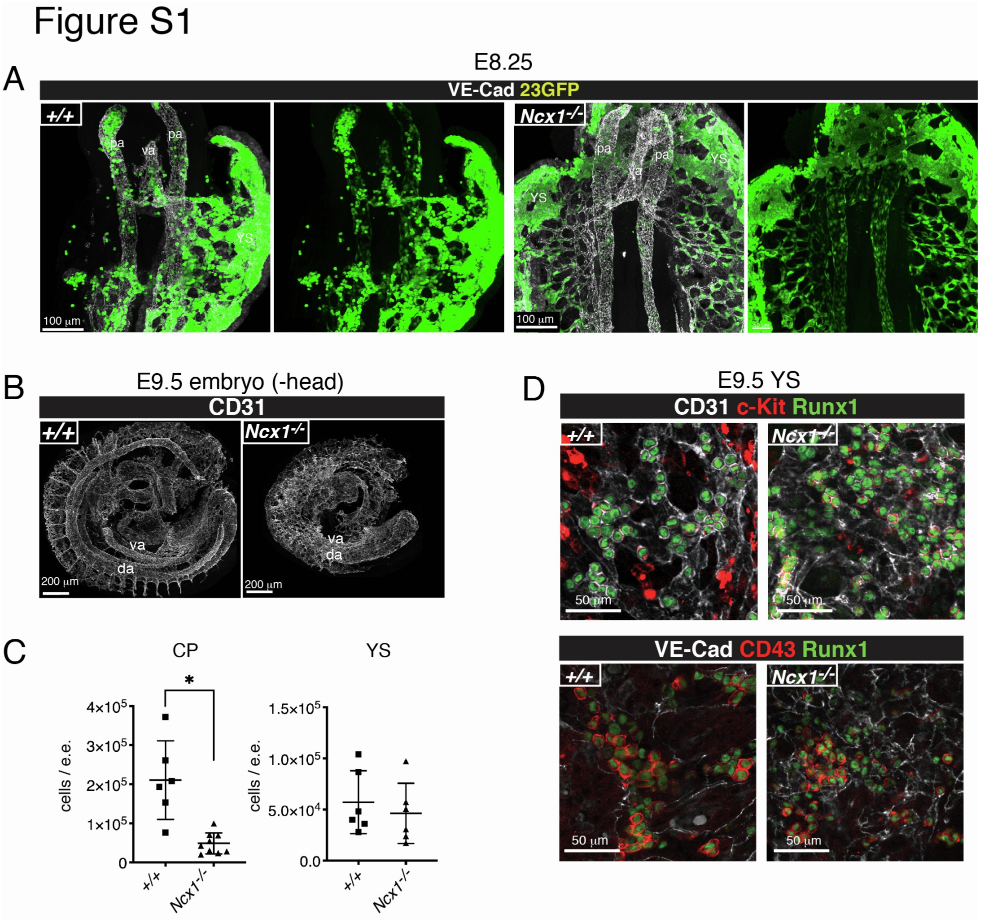

Figure S1. Additional phenotypic analysis of E9.5 *Ncx1*$^{-/-}$ embryos and yolk sacs, Related to Figure 1

A. Confocal whole mount immunofluorescence (WM-IF) analysis of E8.25 (4-8sp) wild type (+/+) and *Ncx1*$^{-/-}$ concepti (embryo and yolk sac). Paired aortae (pa); vitelline artery (va). Abundant 23GFP expression can be seen in the yolk sac (YS) of both genotypes, reflecting expression in primitive erythroid cells and a subset of endothelium; in the paired aortae 23GFP marks pre-HE (Swiers et al., 2013). Note the absence of 23GFP^{+} primitive erythrocytes in the paired aortae of the *Ncx1*$^{-/-}$ embryo. N=2 (+/+), N=2 (*Ncx1*$^{-/-}$) embryos analyzed. Scale bars: 100µm.

B. Confocal WM-IF analysis of E9.5 (23-24sp) wild type (+/+) and *Ncx1*$^{-/-}$ embryos. 3D maximum intensity projection is shown. va: vitelline artery; da: dorsal aorta. Scale bars: 200□m. N≥8 (+/+), N≥8 (*Ncx1*$^{-/-}$).

C. Total live cell numbers of wild type (+/+) and *Ncx1*$^{-/-}$ E9.5 (21-27sp) caudal parts (CP) and yolk sac (YS). N=6 (+/+), N=9 (*Ncx1*$^{-/-}$) CP; N=6 (+/+), N=6 (*Ncx1*$^{-/-}$) YS. Data are represented as mean±SD.

D. Confocal WM-IF analysis of E9.5 (21-26sp) wild type (+/+) and *Ncx1*$^{-/-}$ YS, showing a single 2.5µm-thick optical slice. N=4 (+/+), N=3 (*Ncx1*$^{-/-}$) (top); N=2 (+/+), N=2 (*Ncx1*$^{-/-}$) (bottom) embryos analyzed. Scale bars: 50µm.

Figure S2

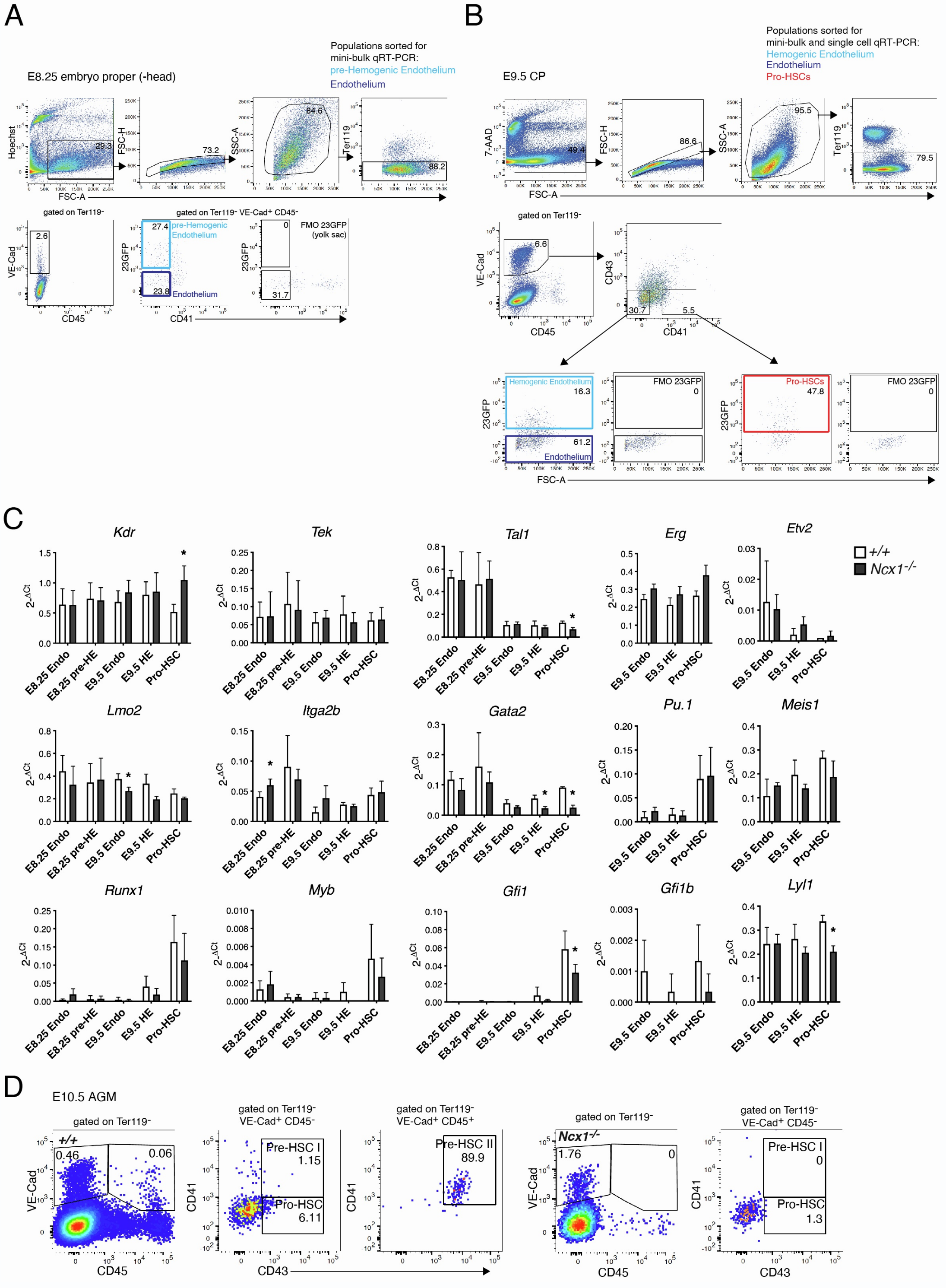
A
E8.25 embryo proper (-head)
Populations sorted for
mini-bulk qRT-PCR:
pre-Hemogenic Endothelium
Endothelium
Hoechst
FSC-A
FSC-H
SSC-A
Ter119
29.3
73.2
84.6
88.2
gated on Ter119-
gated on Ter119- VE-Cad+ CD45-
VE-Cad
CD45
2.6
23GFP
CD41
27.4
pre-Hemogenic Endothelium
23.8
Endothelium
FMO 23GFP
(yolk sac)
0
31.7
B
E9.5 CP
Populations sorted for
mini-bulk and single cell qRT-PCR:
Hemogenic Endothelium
Endothelium
Pro-HSCs
7-AAD
49.4
86.6
95.5
79.5
gated on Ter119-
6.6
CD43
30.7
5.5
Hemogenic Endothelium
16.3
61.2
FMO 23GFP
Pro-HSCs
47.8
C
+/+
Ncx1-/-
Kdr
Tek
Tal1
Erg
Etv2
Lmo2
Itga2b
Gata2
Pu.1
Meis1
Runx1
Myb
Gfi1
Gfi1b
Lyl1
2-ΔCt
E8.25 Endo
E8.25 pre-HE
E9.5 Endo
E9.5 HE
Pro-HSC
D
E10.5 AGM
gated on Ter119-
+/+
0.46
0.06
gated on Ter119-
VE-Cad+ CD45-
Pre-HSC I
1.15
Pro-HSC
6.11
gated on Ter119-
VE-Cad+ CD45+
Pre-HSC II
89.9
Ncx1-/-
1.76
0
Pre-HSC I
0
Pro-HSC
1.3
CD43

Figure S2. Gating strategy and additional qRT-PCR and flow cytometry analysis of wild type and *Ncx1*$^{-/-}$ embryos, Related to Figure 2

A. Example of FACS gating strategy used for isolation of cells for downstream mini-bulk qRT-PCR from E8.25 embryos. A *Ncx1*$^{+/+}$ 23GFP-transgenic sample is shown. FMO 23GFP is a full staining performed on wild type or *Ncx1*$^{+/-}$ samples not carrying the 23GFP transgene. Endothelium (Ter119^{-} VE-Cad^{+} CD45^{-} CD41^{-} 23GFP^{-}); pre-hemogenic endothelium (Ter119^{-} VE-Cad^{+} CD45^{-} CD41^{-} 23GFP^{+}).

B. Example of FACS gating strategy used for isolation of cells for downstream single cell or mini-bulk bulk qRT-PCR from E9.5 embryos. Endothelium (Ter119^{-} VE-Cad^{+} CD45^{-} CD41^{-} CD43^{-} 23GFP^{-}), Hemogenic endothelium (Ter119^{-} VE-Cad^{+} CD45^{-} CD41^{-} CD43^{-} 23GFP^{+}), Pro-HSCs (Ter119^{-} VE-Cad^{+} CD45^{-} CD41^{low} CD43^{-} 23GFP^{+}). A *Ncx1*$^{+/+}$ 23GFP-transgenic sample is shown. FMO 23GFP is a full staining performed on wild type or *Ncx1*$^{+/-}$ samples not carrying the 23GFP transgene. Refer to individual experiments for replicate and embryo numbers.

C. Multiplexed mini-bulk qRT-PCR analysis performed on pools of 25 cells. E8.25 pre-hemogenic endothelium (pre-HE; Ter119^{-} VE-Cad^{+} CD45^{-} CD41^{-} 23GFP^{+}); E8.25 endothelium (Endo; Ter119^{-} VE-Cad^{+} CD45^{-} CD41^{-} 23GFP^{-}); E9.5 endothelium (Endo; Ter119^{-} VE-Cad^{+} CD45^{-} CD41^{-} CD43^{-} 23GFP^{-}); E9.5 hemogenic endothelium (HE; Ter119^{-} VE-Cad^{+} CD45^{-} CD41^{-} CD43^{-} 23GFP^{+}); E9.5 pro-HSCs (Ter119^{-} VE-Cad^{+} CD45^{-} CD41^{low} CD43^{-} 23GFP^{+}). Number of biological replicates and embryos as follows:

E8.25: N=3 (+/+, 3-10sp), N=4 (*Ncx1*$^{-/-}$, 6-11sp) biological replicates over 3 independent sorting experiments. 2 to 5 embryos of the same genotype were pooled. Total number of embryos used: 11 (+/+), 12 (*Ncx1*$^{-/-}$);

E9.5 except *Runx1*: N=3 (+/+, 21-26sp), N=3 (*Ncx1*$^{-/-}$, 22-25sp) biological replicates over 2 independent sorting experiments. 1 to 3 embryos of the same genotype were pooled. Total number of embryos used: 7 (+/+), 5 (*Ncx1*$^{-/-}$);

E9.5 *Runx1*: N=7 (+/+, 21-26sp), N=7 (*Ncx1*$^{-/-}$, 22-25sp) biological replicates over 5 independent sorting experiments. 1 to 3 embryos of the same genotype were pooled. Total number of embryos used: 13 (+/+), 15 (*Ncx1*$^{-/-}$). Data are shown as mean ±SD.

D. Flow cytometric analysis of E10.5 control (*Ncx1*$^{+/+}$ or *Ncx1*$^{+/-}$) or *Ncx1*$^{-/-}$ AGM. Wild type embryos had 35-39sp. It was not possible to count somite pairs in littermate E10.5 *Ncx1*$^{-/-}$ embryos as they were not visible. N=7 (+/+ or *Ncx1*$^{+/-}$), N=2 (*Ncx1*$^{-/-}$) over 2 independent experiments.

Figure S3

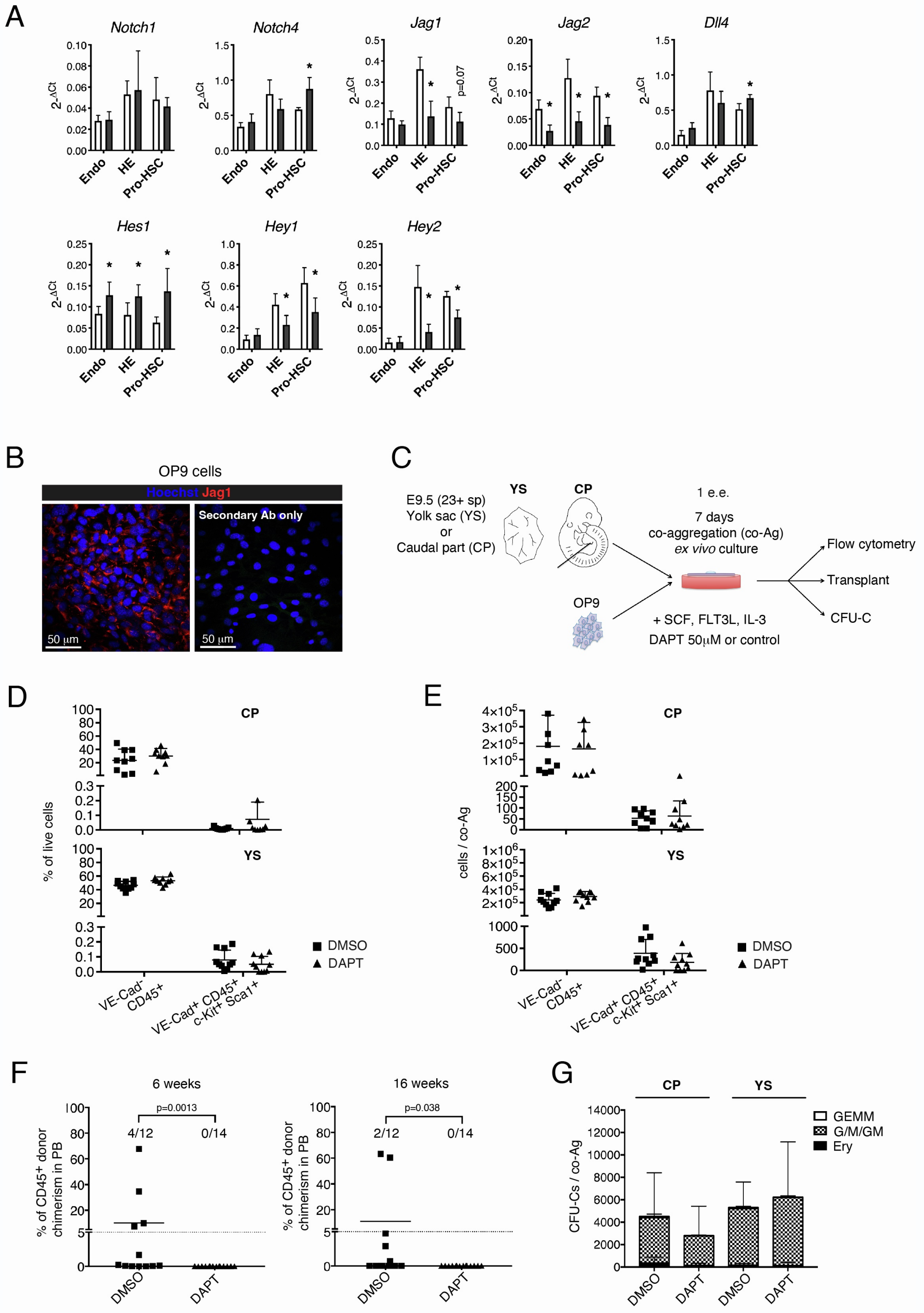
A
Notch1
Notch4
Jag1
Jag2
Dll4
Hes1
Hey1
Hey2
2-ΔCt
Endo
HE
Pro-HSC
p=0.07
B
OP9 cells
Hoechst Jag1
Secondary Ab only
50 μm
C
E9.5 (23+ sp)
Yolk sac (YS)
or
Caudal part (CP)
YS
CP
OP9
1 e.e.
7 days
co-aggregation (co-Ag)
ex vivo culture
+ SCF, FLT3L, IL-3
DAPT 50μM or control
Flow cytometry
Transplant
CFU-C
D
% of live cells
CP
YS
DMSO
DAPT
VE-Cad- CD45+
VE-Cad+ CD45+ c-Kit+ Sca1+
E
cells / co-Ag
CP
YS
DMSO
DAPT
VE-Cad- CD45+
VE-Cad+ CD45+ c-Kit+ Sca1+
F
6 weeks
16 weeks
% of CD45+ donor chimerism in PB
p=0.0013
p=0.038
4/12
0/14
2/12
0/14
DMSO
DAPT
G
CP
YS
CFU-Cs / co-Ag
GEMM
G/M/GM
Ery
DMSO
DAPT

Figure S3. The Notch pathway is required along the pro-HSC to HSC maturation route, but it is dispensable for hematopoietic progenitors, Related to Figure 2.

A. Multiplexed qRT-PCR analysis performed on pools of 25 cells. E9.5 endothelium (Endo; $Ter119^-$ $VE\text{-}Cad^+$ $CD45^-$ $CD41^-$ $CD43^-$ $23GFP^-$); E9.5 hemogenic endothelium (HE; $Ter119^-$ $VE\text{-}Cad^+$ $CD45^-$ $CD41^-$ $CD43^-$ $23GFP^+$); E9.5 pro-HSCs ($Ter119^-$ $VE\text{-}Cad^+$ $CD45^-$ $CD41^{low}$ $CD43^-$ $23GFP^+$). Sort gates as in Figure S2. N=4 (+/+, 22-26sp), N=4 (*Ncx1*$^{-/-}$, 22-25sp) biological replicates over 3 independent sorting experiments. 1 to 3 embryos of the same genotype were pooled. Total number of embryos used: 6 (+/+), 10 (*Ncx1*$^{-/-}$). Data are shown as mean±SD.

B. Immunofluorescence analysis of OP9 cells using a Jag1-specific antibody. A secondary only control (donkey anti-goat AF555) is shown. Scale bar: 50μm.

C. Schematic of co-aggregate cultures in wild type embryos with or without addition of DAPT.

D-E. Flow cytometric analysis of OP9 co-aggregates from caudal part (CP) and yolk sac (YS) of E9.5 (23-26sp) wild type embryos, treated with 50μM DAPT or DMSO. Cells were gated as $7AAD^-$ $Ter119^-$; the gating strategy was the same as in Figure 4B. Graphs show percentages of live cells (D) or absolute numbers (E) of selected populations. OP9 co-aggregates were made with 1 e.e. of caudal part or YS cells and were analyzed individually. N=9 (DMSO), N=9 (DAPT) for CP, N=10 (DMSO), N=10 (DAPT) for YS over 3 independent experiments. Statistical tests showed no significant differences between control and DAPT-treated samples. Error bars represent mean±SD.

F. Repopulation analysis of irradiated CD45.1 syngeneic mice transplanted with 1 embryo equivalent (e.e.) of E9.5 (23-28sp) wild type $CD45.2^+$ caudal part cells after co-aggregation culture, treated with 50μM DAPT or DMSO. Graphs shows peripheral blood (PB) chimerism represented as the percentage of donor $CD45.2^+$ cells among total $CD45^+$ cells, 6 (left) and 16 weeks (right) after transplant. Numbers of transplanted mice as shown. Data from 3 independent transplant experiments. Lines represent the mean.

G. CFU-C obtained from OP9 co-aggregates with caudal part and yolk sac of E9.5 (23-26sp) wild type embryos, treated with 50μM DAPT or DMSO. N=9 (DMSO), N=9 (DAPT) for CP, N=10 (DMSO), N=10 (DAPT) for YS over 3 independent experiments. GEMM: granulocyte, erythroid, monocyte/macrophage, megakaryocyte; G/M/GM: granulocyte, monocyte/macrophage; Ery: erythroid. Statistical tests showed no significant differences between control and DAPT-treated samples. Data are represented as mean± SD.

Figure S4

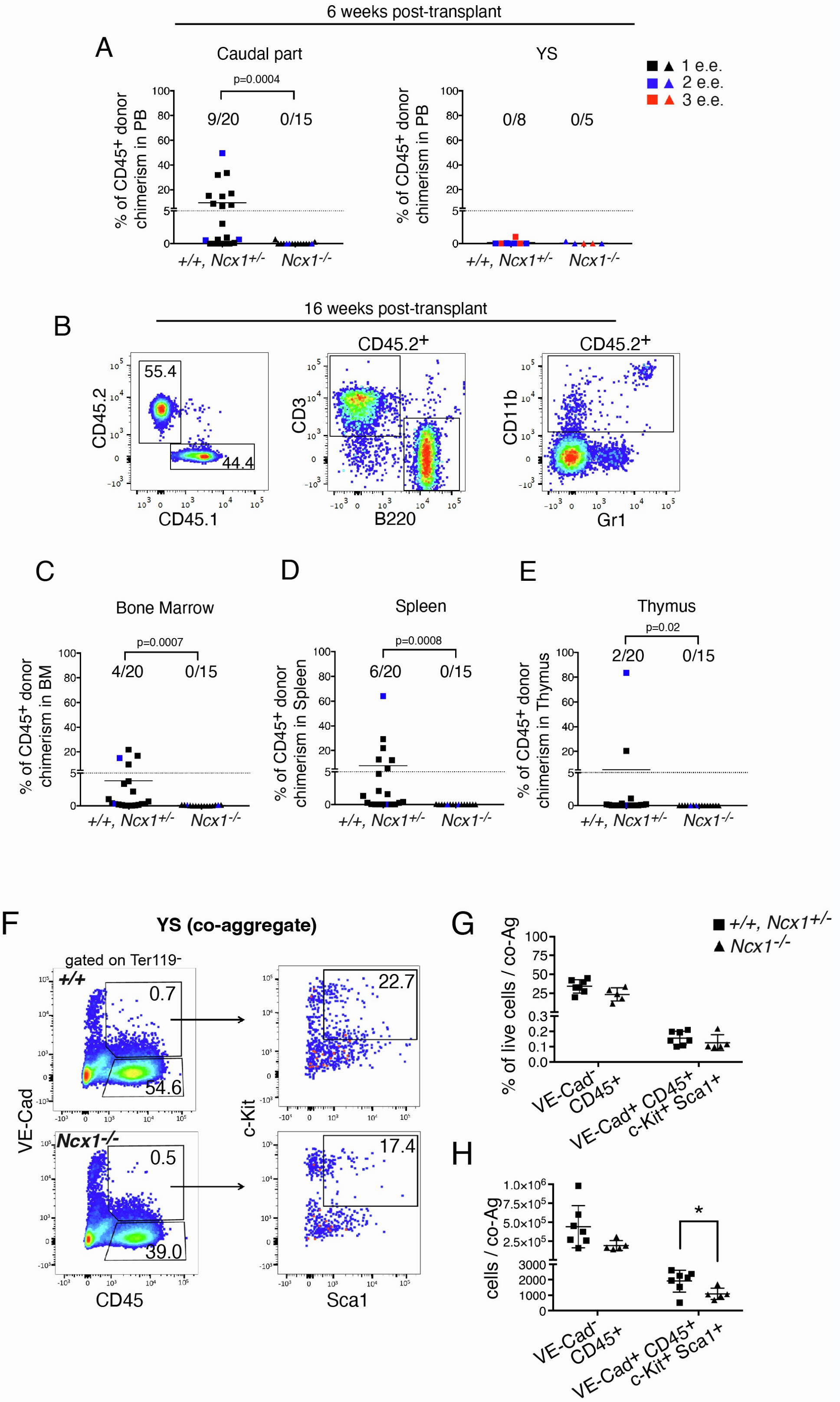
6 weeks post-transplant
A
Caudal part
YS
p=0.0004
9/20
0/15
0/8
0/5
1 e.e.
2 e.e.
3 e.e.
% of CD45+ donor chimerism in PB
+/+, Ncx1+/-
Ncx1-/-
16 weeks post-transplant
B
55.4
44.4
CD45.2
CD45.1
CD45.2+
CD3
B220
CD11b
Gr1
C
D
E
Bone Marrow
Spleen
Thymus
p=0.0007
p=0.0008
p=0.02
4/20
0/15
6/20
2/20
% of CD45+ donor chimerism in BM
% of CD45+ donor chimerism in Spleen
% of CD45+ donor chimerism in Thymus
F
YS (co-aggregate)
gated on Ter119-
+/+
Ncx1-/-
0.7
54.6
22.7
0.5
39.0
17.4
VE-Cad
CD45
c-Kit
Sca1
G
% of live cells / co-Ag
■ +/+, Ncx1+/-
▲ Ncx1-/-
VE-Cad- CD45+
VE-Cad+ CD45+ c-Kit+ Sca1+
H
cells / co-Ag
*

Figure S4. Additional analysis of co-aggregates, Related to Figure 4

A. Repopulation analysis of irradiated CD45.1 syngeneic mice transplanted with 1-3 e.e. of E9.5 (21-27sp) control (*Ncx1*$^{+/+}$ or *Ncx1*$^{+/-}$) or *Ncx1*$^{-/-}$ CD45.2^{+} caudal part or yolk sac cells after co-aggregate culture. Graphs shows peripheral blood (PB) chimerism represented as the percentage of donor CD45.2^{+} cells on total CD45^{+} cells, 6 weeks after transplant. Number of transplanted mice as shown. Lines represent the mean.

B. Flow cytometry analysis of peripheral blood at 16 weeks post-transplant showing a representative example of a recipient reconstituted with wild type (+/+) cells.

C-E. Repopulation analysis as in (A), showing percentage of chimerism in bone marrow (C), spleen (D) and thymus (E), 16 weeks after transplant. Black squares or triangles: recipient transplanted with 1 e.e. of donor cells; Blue squares or triangles: recipient transplanted with 2 e.e. of donor cells. Lines represent the mean.

F. Flow cytometric analysis of OP9 co-aggregates from E9.5 (24-28sp) control (*Ncx1*$^{+/+}$ or *Ncx1*$^{+/-}$) and *Ncx1*$^{-/-}$ yolk sacs. Percentages of individual gates are shown in dot plots. Cells were gated as 7AAD^{-} Ter119^{-}. OP9 co-aggregates were made with 1 e.e. YS cells and were analyzed individually. N=7 (+/+ or *Ncx1*$^{+/-}$), N=5 (*Ncx1*$^{-/-}$) over 3 independent experiments.

G-H. Graphs of flow cytometric analysis in (F) showing percentage within live cells (G) and absolute numbers (H) of VE-Cad^{-} CD45^{+} and VE-Cad^{+} CD45^{+} c-Kit^{+} Sca1^{+} cells. Error bars in the graphs represent mean±SD.

Figure S5

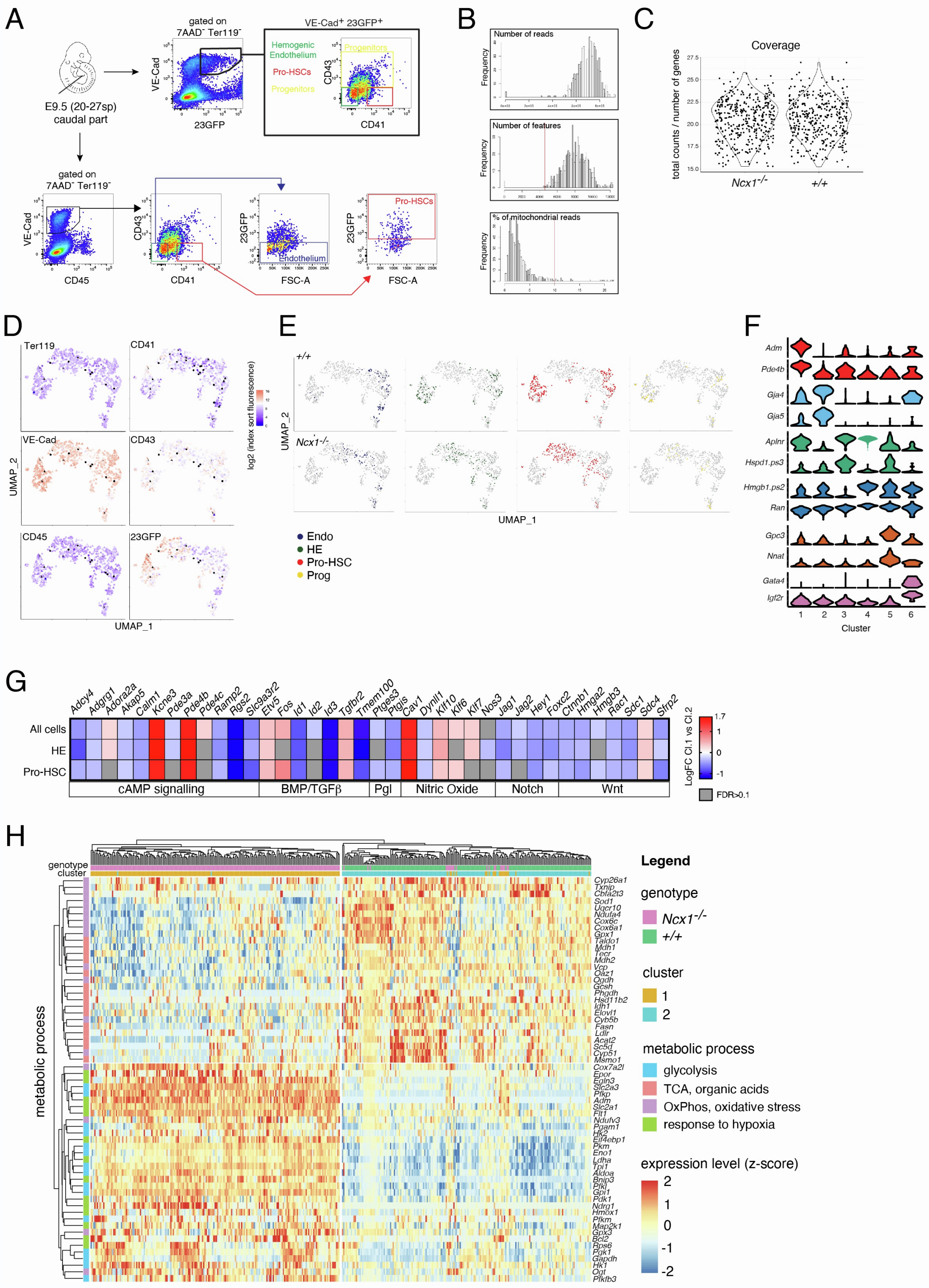
A
E9.5 (20-27sp) caudal part
gated on 7AAD- Ter119-
VE-Cad+ 23GFP+
Hemogenic Endothelium
Pro-HSCs
Progenitors
Endothelium
B
Number of reads
Number of features
% of mitochondrial reads
Frequency
C
Coverage
total counts / number of genes
Ncx1-/-
+/+
D
Ter119
CD41
VE-Cad
CD43
CD45
23GFP
UMAP_1
UMAP_2
log2 (index sort fluorescence)
E
Endo
HE
Pro-HSC
Prog
F
Adm
Pde4b
Gja4
Gja5
Aplnr
Hspd1.ps3
Hmgb1.ps2
Ran
Gpc3
Nnat
Gata4
Igf2r
Cluster
G
All cells
HE
Pro-HSC
cAMP signalling
BMP/TGFβ
Pgl
Nitric Oxide
Notch
Wnt
LogFC Cl.1 vs Cl.2
FDR>0.1
H
genotype
cluster
metabolic process
Legend
glycolysis
TCA, organic acids
OxPhos, oxidative stress
response to hypoxia
expression level (z-score)

Figure S5. Sort gates and additional Smart-Seq2 scRNA-seq analysis, Related to Figure 5

A. Schematic of the gates used for single cell FACS sorting. Endothelium (7-AAD$^-$ Ter119$^-$ VE-Cadherin$^+$ CD45$^-$ CD43$^-$ CD41$^-$ 23GFP$^-$) and pro-HSCs (7-AAD$^-$ Ter119$^-$ VE-Cadherin$^+$ CD45$^-$ CD43$^-$ CD41^{low} 23GFP$^+$) were sorted directly. 7-AAD$^-$ Ter119$^-$ VE-Cad$^+$ 23GFP$^+$ were index sorted and assigned to HE (7-AAD$^-$ Ter119$^-$ VE-Cadherin$^+$ CD45$^-$ CD43$^-$ CD41$^-$ 23GFP$^+$), pro-HSC (7-AAD$^-$ Ter119$^-$ VE-Cadherin$^+$ CD45$^-$ CD43$^-$ CD41^{low} 23GFP$^+$) and progenitor (7-AAD$^-$ Ter119$^-$ VE-Cadherin$^+$ CD45$^-$ CD43$^+$ CD41$^{+/-}$ 23GFP$^+$) gates. 26 E9.5 wild type (+/+*;* 21-27sp) and 19 mutant (*Ncx1*$^{-/-}$; 20-25sp) embryos were used. Cells were collected in 6 different sorting experiments.

B. Histograms showing number of reads, feature counts and percentage of mitochondrial reads across the Smart-Seq2 scRNA-seq dataset. Cells with less than 5.2×10^5 reads or 4600 features or more than 10% of mitochondrial reads (red line in the histograms) were excluded from the rest of the analysis.

C. Violin plot showing representative coverage values in the two genotypes (+/+ and *Ncx1*$^{-/-}$). For each cell, coverage is calculated as the total number of counts divided by the number of genes detected in that cell. This value represents how many counts are assigned to an individual gene on average.

D. UMAP projections of surface marker values obtained by index sorting, color coded on each cell. Black dots indicate 15 cells with missing index sort data (6 cells directly sorted as endothelial cells and 9 directly sorted as pro-HSCs).

E. UMAP projections highlighting the four phenotypically identified cell populations (Endo, HE, Pro-HSC and progenitors) with different colors, as indicated. Each of the four populations is shown separately for the two genotypes.

F. Violin plots showing expression levels of the top two genes driving clustering, across the six clusters. Each row of violin plots is color coded as in Figure 5A. Colors indicate the cluster in which each gene is enriched.

G. Heatmap representing selected genes differentially expressed (FDR<0.1) between cluster 1 (*Ncx1*$^{-/-}$) and 2 (+/+), divided in groups of genes involved in signalling pathways as indicated. cAMP genes were downregulated in *Ncx1*$^{-/-}$ cells, except for *Kcne3* and *Pde4b*, which are known to be induced by hypoxia in several cell types (Copple et al., 2011; Millen et al., 2006). Nitric oxide genes showed a mixed response, with *Nos3* (endothelial nitric oxide synthase) downregulated only in *Ncx1*$^{-/-}$ HE. Id genes were downregulated in *Ncx1*$^{-/-}$ cells, as well as the BMP target *Tmem100*, an arterial gene that when deleted showed vascular remodeling defects (Somekawa et al., 2012) similar to those seen in *Ncx1*$^{-/-}$ embryos. The prostaglandin and Wnt genes were downregulated in the *Ncx1*$^{-/-}$ cells, except for the Wnt negative regulator *Sdc4,* which was upregulated (Astudillo et al., 2014). Values are shown as logFC (fold change) and are calculated taking into account all cells in cluster 1 and 2, or only HE or pro-HSC in the same clusters. Grey cells indicate genes that are not differentially expressed (FDR>0.1) in that population.

H. Heatmap of selected genes differentially expressed between cluster 1 and cluster 2 (FDR<0.1). Rows represent genes; columns represent single cells. The color coding indicates genotypes (+/+ and *Ncx1*$^{-/-}$), clusters (1 or 2), and groups of metabolic processes (glycolysis, TCA and organic acids, OxPhos and oxidative stress, and response to hypoxia). Expression levels are shown as z-scores. TCA: tricarboxylic acid cycle; OxPhos: oxidative phosphorylation.

Figure S6

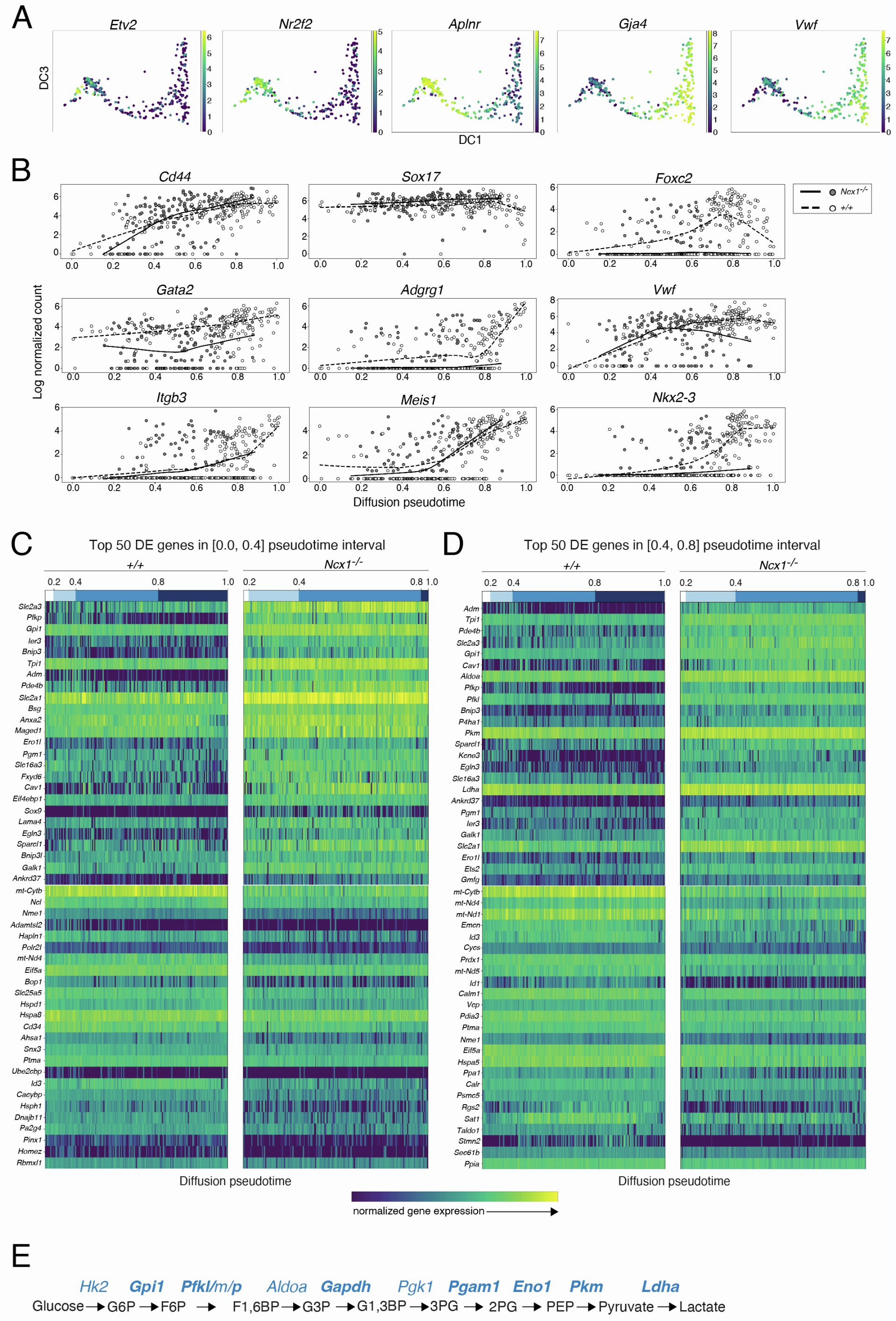
A
Etv2
Nr2f2
Aplnr
Gja4
Vwf
DC3
DC1
B
Cd44
Sox17
Foxc2
Ncx1-/-
+/+
Gata2
Adgrg1
Vwf
Itgb3
Meis1
Nkx2-3
Log normalized count
Diffusion pseudotime
C
Top 50 DE genes in [0.0, 0.4] pseudotime interval
D
Top 50 DE genes in [0.4, 0.8] pseudotime interval
Diffusion pseudotime
normalized gene expression
E
Hk2 Gpi1 Pfkl/m/p Aldoa Gapdh Pgk1 Pgam1 Eno1 Pkm Ldha
Glucose → G6P → F6P → F1,6BP → G3P → G1,3BP → 3PG → 2PG → PEP → Pyruvate → Lactate

Figure S6. Additional pseudotime analysis of Smart-seq2 scRNA-seq data, Related to Figure 6

A. scRNA-seq diffusion maps of wild type cells, in which expression of selected genes is superimposed. Each dot represents an individual cell. Gene expression levels are color coded and shown as Log (normalized counts). DC: diffusion component.

B. Scatter plots showing expression of selected genes involved in hemogenic endothelium and hematopoietic development along diffusion pseudotime. Each dot represents an individual cell. Gene expression is shown in the y-axis as Log (normalized counts). Lines fitting the expression of genes over the pseudotime are obtained by locally weighted linear regression.

C-D. Heatmaps showing the top 50 genes differentially expressed between wild type and *Ncx1*$^{-/-}$ cells in the EHT trajectory (25 upregulated and 25 downregulated in *Ncx1*$^{-/-}$ versus wild type), determined separately for two regions of pseudotime: 0.0 to 0.4 (B) and 0.4 to 0.8 (C). Gene expression is color coded and shown as Log (normalized counts). Values were rescaled on a scale of 0 to 1 for visualization purpose.

E. Schematic of the glycolysis pathway, in which the genes in bold are significantly downregulated in wild type cells in the second pseudotime region (0.4 to 1.0) compared to the first (0.0 to 0.4) – see table S3.

Figure S7

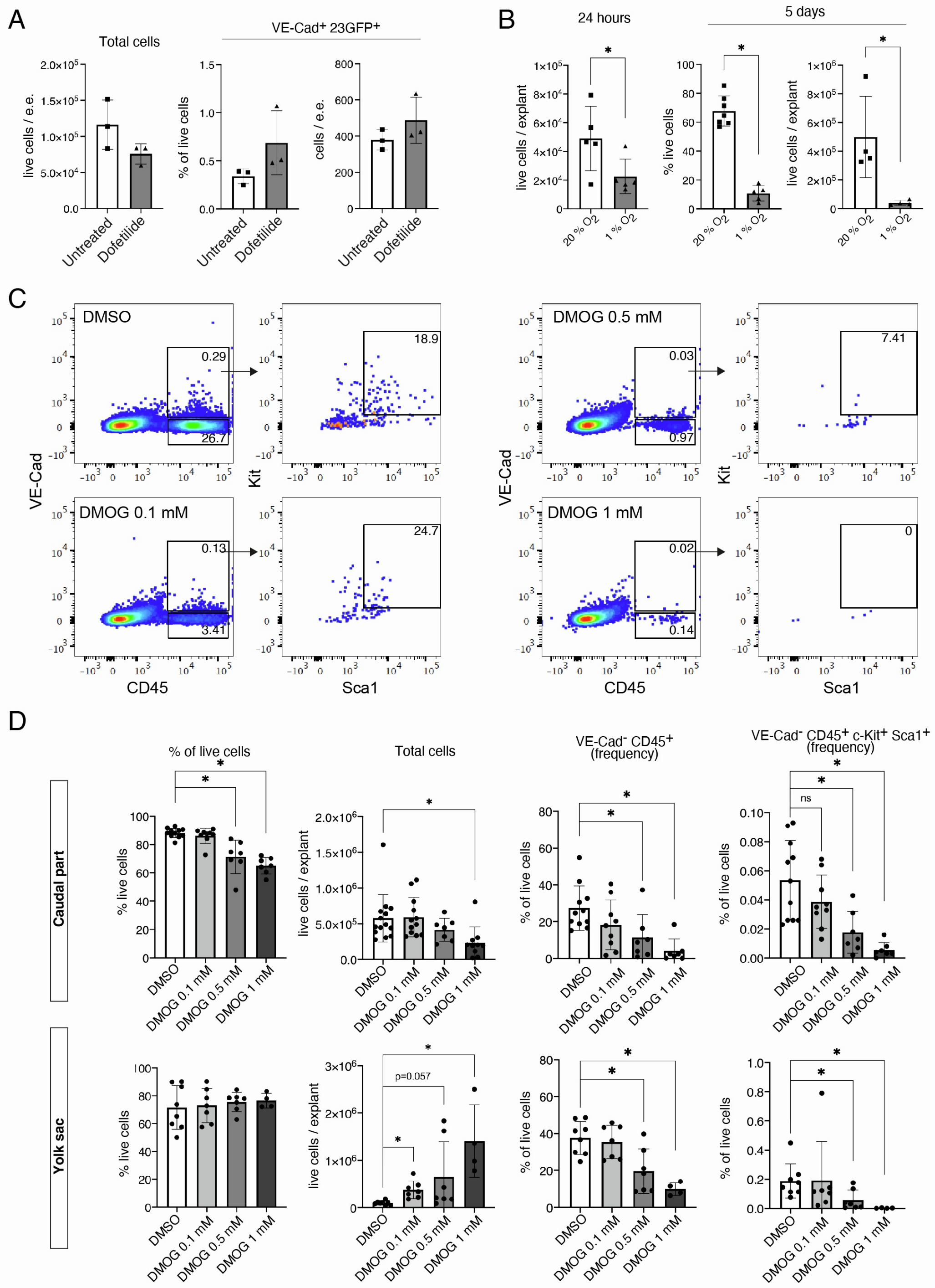

A
Total cells
VE-Cad+ 23GFP+
live cells / e.e.
% of live cells
cells / e.e.
Untreated
Dofetilide
B
24 hours
5 days
live cells / explant
% live cells
20 % O2
1 % O2
C
DMSO
DMOG 0.5 mM
DMOG 0.1 mM
DMOG 1 mM
VE-Cad
Kit
CD45
Sca1
D
% of live cells
Total cells
VE-Cad- CD45+ (frequency)
VE-Cad- CD45+ c-Kit+ Sca1+ (frequency)
Caudal part
Yolk sac
% live cells
live cells / explant
% of live cells
p=0.057
ns
DMSO
DMOG 0.1 mM
DMOG 0.5 mM
DMOG 1 mM

Figure S7. Additional analyses of dofetilide-treated embryos and hypoxia and DMOG-treated explant cultures, Related to Figure 7.

A. Cell counts and flow cytometric analysis of freshly isolated E9.5 (21-27sp) embryos from control and dofetilide-treated females. Cells were gated as live Ter119$^-$ VE-Cad$^+$ 23GFP$^+$. Numbers of replicates as shown. Data from 2 independent experiments. Statistical tests showed no significant differences between control and treated samples. Data are mean±SD.

B. Live cell counts of hypoxia explant cultures. N=5 (20% O_2), N=5 (1% O_2), 4 independent experiments for 24-hour cultures. Number of replicates as shown, 4 independent experiments for 5-day cultures. Data are mean±SD.

C. Representative dot plots of explant cultures of wild type E9.5 (19-26sp) embryos cultured in presence of a range of concentrations of DMOG. Cells were gated as Live Ter119$^-$. All dot plots are taken from the same experiment.

D. Graphs summarizing cell counts and flow cytometry analysis of DMOG explant cultures, 7 days after culture. Numbers of replicates as shown. Data from 4 independent experiments (CP and YS). Data are mean±SD.
